# Independent origins of spicules reconcile paleontological and molecular evidence of sponge evolutionary history

**DOI:** 10.1126/sciadv.adx1754

**Published:** 2026-01-07

**Authors:** Maria Eleonora Rossi, Joseph N. Keating, Nathan J. Kenny, Mattia Giacomelli, Sandra Álvarez-Carretero, Astrid Schuster, Paco Cárdenas, Sergi Taboada, Vasiliki Koutsouveli, Philip C. J. Donoghue, Ana Riesgo, Davide Pisani

**Affiliations:** ^1^ Bristol Palaeobiology Group, School of Earth Sciences, University of Bristol, Life Sciences Building, Tyndall Avenue, Bristol BS8 1TH, UK.; ^2^ Centre for Chromosome Biology, School of Biological and Chemical Sciences, University of Galway, Galway H91 W2TY, Ireland.; ^3^ Life Sciences Department, The Natural History Museum, London SW7 5BD, UK.; ^4^ Biochemistry Department, School of Biomedical Sciences, University of Otago, Dunedin, Aotearoa, New Zealand.; ^5^ Departament de Genètica, Microbiologia i Estadística, Facultat de Biologia, Universitat de Barcelona, 08028 Barcelona, Spain.; ^6^ Department of Biology, University of Southern Denmark, Nordcee, Campusvej 55, Odense M 5230, Denmark.; ^7^ Pharmacognosy, Department of Medicinal Chemistry, Uppsala University, Husargatan 3, Uppsala 751 23, Sweden.; ^8^ Museum of Evolution, Uppsala University, Norbyvägen 16, Uppsala 752 36, Sweden.; ^9^ Department of Biodiversity and Evolutionary Biology, National Museum of Natural Sciences (CSIC), c/José Gutiérrez Abascal 2, 28006 Madrid, Spain.; ^10^ Departamento de Biodiversidad, Ecología y Evolución, Facultad de Ciencias, Universidad Complutense de Madrid, Madrid, Spain.; ^11^ Departamento de Ciencias de la Vida, EU-US Marine Biodiversity Group, Universidad de Alcalá, Alcalá de Henares, Spain.; ^12^ Division of Marine Ecology, Marine Evolutionary Ecology, GEOMAR Helmholtz Centre for Ocean Research Kiel, Kiel, Germany.; ^13^ Bristol Palaeobiology Group, School of Biological Sciences, University of Bristol, Life Sciences Building, Tyndall Avenue, Bristol BS8 1TH, UK.

## Abstract

Sponges (Porifera) are ecosystem engineers that play a critical role in global biogeochemical processes. Their evolution is key to understanding Neoproterozoic paleoecology but remains mired in controversy. Molecular timescales suggest a Tonian or Cryogenian origin, while their oldest unequivocal fossils consist of disarticulated siliceous spicules from the Late Ediacaran. We derived a new, dated sponge phylogeny and tested whether ancestral sponges had mineralized skeletons. We resolve the sponge phylogeny in good agreement with current knowledge and date their origin to the early Ediacaran. Our results suggest that early sponges were not biomineralized and that both biosilicification and biocalcification evolved independently multiple times across Porifera. We reconcile fossil evidence and molecular estimates of sponge evolution by showing that the Neoproterozoic history of Porifera is limited to the Ediacaran and providing evidence suggesting that sponges are largely absent from the Ediacaran record because they were yet to evolve biomineralized skeletons.

## Teaser

Sponges have a cryptic Ediacaran history because ancestral sponges were soft-bodied and had low fossilization potential.

## INTRODUCTION

The sponge animal phylum, Porifera, is composed of four extant lineages (Calcarea, Homoscleromopha, Demospongiae, and Hexactinellida) sharing a body plan characterized by a system of pores (ostia) and channels that allow water to circulate through them. Sponges provide key ecosystem services, from habitat formation to nutrient and silica cycling, water filtering, and substrate stabilization (*1*). Phylogenetically, sponges are either the sister group of all the other animals or else the second phylum to branch from the animal crown-ancestor (*2*–*8*). As such, sponges are key to understanding the time of emergence, body plan, and ecology of the ancestral animal. However, early sponge evolution is the subject of controversy. Molecular divergence time and fossil biomarker studies have long suggested that sponges diverged in the Tonian or Cryogenian (*9*–*13*) anticipating a deep Neoproterozoic appearance of sponge fossils. Although there are Tonian, Cryogenian, and early Ediacaran fossils with claims on sponge affinity, their interpretations as such range from speculative to contentious (*14*, *15*). For example, anastomosing pores within Tonian carbonates have been interpreted as keratose sponges (*16*) but corroborative anatomical evidence and a preservational model are lacking, and these structures are more readily interpreted as abiotic sedimentary artifacts (*17*). Late Cryogenian C_30_ steranes (fossil biomarkers) interpreted as evidence for Cryogenian demosponges [24-ipc and 26-mes; (*11*)] have also been interpreted as diagenetically altered C_29_ sterols from chlorophyte algae (*18*). Pores in skeletal fragments from the Cryogenian of South Australia have been interpreted as sponge-like ostia (*19*) but again lack corroborating anatomical evidence, while the middle Ediacaran *Eocyathispongia qiania*, originally interpreted as an adult asconoid sponge (*20*), appears to lack the expected internal structure (*21*, *22*). Last, arsenopyrite crystal aggregates from the latest Ediacaran of Mongolia (*23*) have been misinterpreted as siliceous sponge spicules (*24*). There is therefore a minimal mismatch of 150 Myr between most molecular estimates for the time of origin of crown sponges and their first unambiguous fossil evidence, represented by disarticulated siliceous spicules from the latest Ediacaran ca. 543 Ma (*14*, *15*, *25*, *26*).

Three sponge lineages (Hexactinellida, Homoscleromorpha, and Demospongiae) make spicules out of silicic acid and a series of previous molecular and genomic analyses have shown that they use different biochemical pathways to do so, suggesting that siliceous spicules might have evolved multiple times independently (*27*–*30*). The fourth sponge lineage, Calcarea, makes spicules using calcium carbonate, usually calcite but sometimes aragonite (*31*, *32*). Skeletal elements made of calcium carbonate (aragonite rather than calcite) are also found in sclerotized demosponges [represented in our dataset by the keratose *Vaceletia*; (*33*)]*.* However, the skeleton of sclerotized demosponges is not composed of spicules. Some fossil sponges (stromatoporoids) built their skeletons using calcium phosphate, making the sponges the first phylum that could use all three main biominerals (*34*), but there is no evidence of sponges with phosphatic skeletons before the Ordovician. Accordingly, stromatoporoids are not relevant to understanding the early fossil record of sponges. Sponges with skeletal elements made of calcium carbonate (both Calcarea and Demospongiae) use carbonic anhydrase paralogs to precipitate it (*35*–*38*). However, carbonic anhydrase paralogs play key role in many cellular functions [including the regulation of intracellular pH (*32*, *35*, *39*)] and these enzymes seem to have been independently recruited into biocalcification pathways multiple times across Metazoa (*35*). Accordingly, the universal distribution of carbonic anhydrase paralogs across Porifera is not unexpected, and while the last common sponge crown-ancestor certainly had multiple carbonic anhydrase paralogs [perhaps up to eight (*32*, *38*)], this does not constitute evidence for a single origin of biocalcification in sponges. Multiple origins of biocalcification would fit better with the biochemical diversity observed in calcifying sponges for which data are available, which use alternative carbonic anhydrase paralogs (*31*, *37*, *38*).

While the oldest fossil spicules (ca. 543 Ma; late Ediacaran) are made of silica (*14*, *25*, *26*), these siliceous spicules only slightly predate a broader diversity of early sponge fossils (late Ediacaran to early Cambrian) with disparate skeletal structures. These include sponges with a calcareous (aspiculate) skeleton, archaeocyathids (*14*, *40*), biminerallic spicules made of both calcite and silica [e.g., *Eiffelia globosa* (*41*)], and sponges without skeletal elements, including the late Ediacaran *Helicolocellus cantori* [539 Ma; probably a stem hexactinellid (*42*), but see (*43*) for a different opinion] and *Arimasia germsi* [543 to 539 Ma; an archaeocyathid (*44*)]. Hexactinellida and Archaeocyathida (*15*, *40*) are both crown sponges, and these fossils provide direct evidence that Porifera had a Neoproterozoic history, although its duration is unclear.

Fossil evidence alone paints a confusing picture of early sponge evolution, with a limited Late Ediacaran record composed of a mixture of siliceous spicules and body fossils without skeletal elements, making it difficult to rationalize whether the last common sponge ancestor had a biomineralized skeletons with spicules (*26*), and if it had, whether these spicules were made of silica, calcium carbonate, or both (*14*). Alternatively, Ediacaran sponges might have had an aspiculate carbonate skeleton, similarly to Cambrian Archaeocyathida, or an organic skeleton of axial filaments (like some keratose sponges) without biomineralized elements or armed with biomineralized fibers rather than spicules, as in the biosilicified but likely aspiculate Cambrian sponge *Vauxia* (*45*). Soft bodied sponges with a purely organic skeleton would have had low fossilization potential and could have enjoyed a long, cryptic, Neoproterozoic history, potentially extending to the Cryogenian or the Tonian. This uncertainty leaves the problem of reconciling molecular and fossil evidence for the origin of sponges unresolved.

Here, we perform phylogenomic analyses of a new dataset composed of 70 genomes and transcriptomes (12 of which are new to this study). We use our phylogeny to perform molecular clock analyses that integrate new interpretations of the animal fossil record and recent reassessments of key fossil-bearing formations in the rock record (*22*). Last, we test whether late Ediacaran siliceous spicules could be remains of stem or crown sponges using ancestral state estimation methods. Our ancestral state estimations use structured Markov models of character state transformation with embedded dependencies to test a diversity of ways in which biochemical, morphological, and fossil evidence can be integrated when reconstructing ancestral states (*46*, *47*). The use of hierarchical structured models (see Materials and Methods and Supplementary Methods in the Supplementary Materials) also allowed us to investigate whether morphological characters underpinned by different developmental programs (e.g., siliceous spicules underpinned by different biosynthetic pathways) might represent independent origins of the same character, or else reflect a deep homology (*48*) where nonhomologous gene replacements in an ancestral regulatory network led to the emergence of multiple developmental pathways, the homology of the compared characters notwithstanding (*49*).

Our results suggest that sponges originated in the early Ediacaran (ca. 608 Ma), that the last common ancestor of all the sponges was not biomineralized, and that it did not have spicules. Sponges with calcareous skeletons, including calcareous spicules, biminerallic spicules, and sclerotized (aspiculate) skeletons, emerged multiple times independently. Calcareous spicules are limited to Calcarea, a lineage that emerged post-Cambrian (473.24 to 157.52 Ma). Siliceous spicules most likely evolved independently multiple times. With reference to living sponges, siliceous spicules seem to have evolved once in Homoscleromorpha, once in Hexactinellida and twice in Demospongiae (in the stem to the Heteroscleromorpha clade and in the Verongimorpha genus *Chondrilla*). However, the time of origin of biosilicification in Silicea is model-dependent, and if it is assumed that the origin of biosilicification coincided with the origin of siliceous spicules, the last common ancestor of crown Silicea might have already had spicules. Nonetheless, the last common ancestor of all sponges is reconstructed as lacking biomineralized skeletal elements in all our analyses. We performed a focal study, using diversification rate shift analyses on the extant Silicea, which constitutes most extant sponge biodiversity, identifying two potentially significant diversification shifts nested within Heteroscleromorpha, both postdating the emergence of siliceous spicules. Accordingly, at least in the case of Silicea, our diversification shift analyses did not find evidence suggesting that the emergence of siliceous spicules played a role in their diversification.

Our estimates of the timescale of sponge divergence, combined with our study of spicule evolution and previous genomic, biochemical, and paleontological evidence for the evolution of siliceous spicules (*14*, *27*, *29*, *30*, *42*), suggest that while sponges evolved in the early Ediacaran, contradicting previous analyses calibrated using fossil biomarkers, early sponges lacked biomineralized skeletal structures, and had low preservation potential. We conclude that sponges had a cryptic, but relatively short, Neoproterozoic history, which de facto reconciles the sponge fossil record with molecular estimates of divergence times.

## RESULTS

### Resolving the sponge phylogeny

Orthofinder version 2.7.1 (*50*) assigned 75% of the 5,657,552 sequences in our dataset to a total of 335,892 orthogroups. This set of orthogroups was filtered (see Materials and Methods and Supplementary Methods) to remove paralogs. We retained only the orthogroups that had 90% of species left after filtering paralogs out. This resulted in 1468 orthogroups that were further filtered to remove long branched taxa (see Materials and Methods and Supplementary Methods for precise details). A final filtering step was performed to remove families that might include hidden paralogs (see Materials and Methods and Supplementary Methods for details). Our orthogroup filtering strategy left us with a dataset of 70 species (64 belonging to Porifera) and 133 protein coding genes that were used for phylogenetic and molecular clock analyses. The 133 protein coding genes were analyzed independently to generate gene trees and as a concatenated superalignment of 103,269 amino acids (AA). The concatenated dataset was analyzed using maximum likelihood (ML) with the best fitting across-site compositionally homogeneous model in IQTree (*51*) and using Bayesian analysis with the across-site compositionally heterogeneous [CATegories model (*52*), the CAT-Poisson model] in PhyloBayes (*53*). See (*3*, *4*, *7*) for the differences between across-site compositionally homogeneous and heterogeneous models. Last, we used ASTRAL (*54*) on the 133 single gene trees. See https://doi.org/10.6084/m9.figshare.28574570 for the individual single gene alignments, the gene trees and the outputs of all our phylogenetic analyses.

**Fig. 1.**
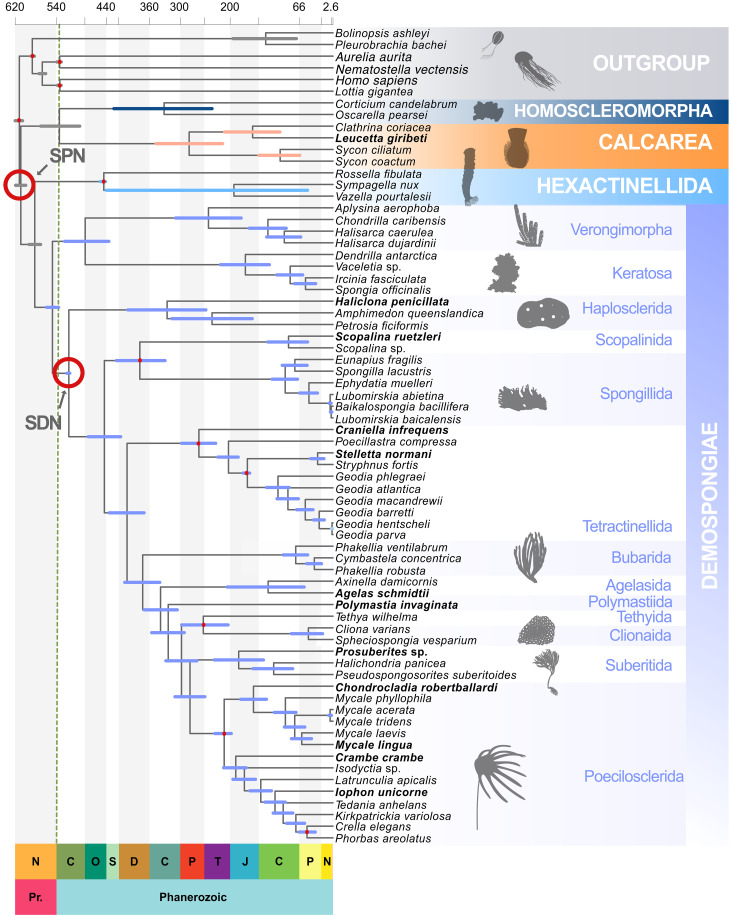
Time-calibrated phylogeny dates the origin of sponges to the early Ediacaran. Porifera timetree with posterior estimates of divergence time estimated using the Bayesian CAT-Poisson tree in fig. S1, under the spicule-bearing Demospongiae (SDN) calibration strategy, with the IR model in MCMCtree. Bars on nodes represent the 95% highest posterior densities. The colors of the bars identify the four main sponge lineages (Calcarea, Homoscleromorpha, Hexactinellida, and Demospongiae). Gray bars indicate internal nodes outside these clades. Red circles represent the two mutually exclusive calibrations strategies Spiculate Porifera Node (SPN) and Spiculate Demosponges Node (SDN). Full red dots represent calibrations used in both strategies. Species in bold were sequences as part of this study. Timescale on *x* axis in hundreds of millions of years. The green dotted line marks the start of the Cambrian. Ne, Neoproterozoic; C, Cambrian; O, Ordovician; S, Silurian; D, Devonian; C, Carboniferous; P, Permian; Tr, Triassic; J, Jurassic; K, Cretaceous; Pe, Paleogene. Support values for the nodes can be found in fig. S1. Differences between our ML, Bayesian, and Astral topologies are described in text and can be investigated comparing the tree here and fig. S1 against those in figs. S2 and S3. Silhouettes are from https://phylopic.org/. Specific attributions: G. Dera is the author of the following silhouettes: *Chrysaora quinquecirrha*, *Plakina jani*, *Leucandra aspera*, *Euplectella aspergillum*, *Homaxinella balfourensis*, and *Spongia officinalis*. *Amphimedon queenslandica* is by seung9park; *Isodyctia grandis* by M. Keesey; Cladorhizidae is by D. Schultz; and *Hormiphora californensis* is by S. Haddock and K. Wothe. All the above mentioned silhouettes have been released under license CC0 1.0 Universal Public Domain Dedication. *Aplysina* is by M. Vingiani (license link: https://creativecommons.org/licenses/by/4.0/). Axinellidae is by Tree of Life App (license link: https://creativecommons.org/licenses/by/4.0/). *Cliona celata* is by M. Eppley (license link: https://creativecommons.org/licenses/by/4.0/). The silhouettes of *Aplysina*, Axinellidae, and *C. celata* were reduced in size and their transparency was enhanced.

Phylogenomic analyses (see Fig. 1 and figs. S1 to S3) unequivocally recovered Porifera to be composed of two sister lineages, Silicea (Hexactinellida plus Demospongiae) and Calcarea plus Homoscleromorpha. We recover Calcarea in ML analyses with ultrafast bootstrap support (UBS) = 100. In Bayesian analyses this clade has posterior probability (PP) = 1, and in Astral analyses it has coalescent branch support (CBS) = 1. Homoscleromorpha has UBS = 100, PP = 1, and CBS = 0.87. The monophyletic group composed of Calcarea and Homoscleromorpha (UBS = 100, PP = 1, and CBS = 0.91), is sister to Silicea (UBS = 100, PP = 1, and CBS = 1), a monophyletic group comprising Hexactinellida and Demospongiae. Within Calcarea, we recover the monophyly of Calcinea and Calcaronea (UBS = 100, PP = 1, and CBS = 1). In our topologies, the Homoscleromorpha (UBS = 100, PP = 1, and CBS = 0.87) is represented by two species from the two families Oscarellidae and Plakinidae, and therefore major relationships were not addressed in detail. We recovered both Demospongiae and Hexactinellida as monophyletic, with full support (UBS = 100, PP = 1, and CBS = 1). For the Hexactinellida, sampling only includes representatives from the order Lyssacinosida.

In Demospongiae, Keratosa and Verongimorpha are monophyletic (UBS = 100, PP = 0.99, and CBS = 0.24) and sister to the Haplosclerida (UBS = 100, PP = 1, and CBS = 1) and Heteroscleromorpha (UBS = 100, PP = 1, and CBS = 1). In the Verongimorpha, we recover *Aplysina aerophoba* (Verongiida) as the sister of Chondrillida, where we recover the genus *Halisarca* to be paraphyletic. *Halisarca dujardini* is sister (UBS = 100, PP = 1, and CBS = 1) to *H. caerulea* and *Chondrilla caribensis* (UBS = 77, PP = 1, and CBS = 1)*.* Within Keratosa, we recover *Dendrilla antarctica* (Dendroceratida) to be the sister to the order Dyctioceratida, which emerges to be monophyletic and fully supported (UBS = 100, PP = 1, and CBS = 1). We recover the monophyly of Haplosclerida (UBS = 100, PP = 1, and CBS = 1), with *Haliclona penicillata* from the family Chalinidae, being sister to *Amphimedon queenslandica* (family Niphatidae) and *Petrosia ficiformis* (family Petrosiidae), all relationships with full support (UBS = 100, PP = 1, and CBS = 1). In the Heteroscleromorpha, the earliest diverging clade is composed of Scopalinida plus Spongillida (UBS = 100, PP = 1, and CBS = 1). In Spongillida, the family Spongillidae is paraphyletic with *Ephydatia muelleri* clustering sister to the family Lubomirskiidae, which is monophyletic (UBS = 100, PP = 1, and CBS = 1). The order Tetractinellida is monophyletic (UBS = 100, PP = 1, and CBS = 1). The suborder Spirophorina, represented by the species *Craniella infrequens*, is sister to the suborder Astrophorina. Astrophorina is monophyletic, with the family Vulcanellidae sister to the families Ancorinidae plus Geodiidae. We recovered Bubarida, as sister to Agelasida, Polymastiida, Tethyida, Clionaida, Suberitida, and Poecilosclerida (UBS = 100, PP = 1, and CBS = 0.96). In the Bayesian tree (Fig. 1 and fig. S1), Clionaida and Tethyida formed a monophyletic sister group to Suberitida and Poecilosclerida, respectively, and the order Suberitida is recovered sister (PP = 1) to the order Poecilosclerida. In the ML (fig. S2) and Astral (fig. S3) topologies, Suberitida is sister (UBS = 68 and CBS = 0.72) to the clade Clionaida plus Tethyida, and Poecilosclerida is sister to these tree orders (UBS = 100 and CBS = 1). In both topologies, the poecilosclerids form two major clades, with the first one composed of the carnivorous sponge *Chondrocladia robertballardi* sister to the family Mycalidae. The second clade comprises all the other families of the order Poecilosclerida.

### Estimating molecular divergence times of sponge evolution

Molecular divergence times (Fig. 1) were inferred using MCMCtree (*55*) for the Bayesian phylogeny (fig. S1), using 12 fossil constraints (see nodes highlighted with red circle in Fig. 1 and detailed descriptions of all 12 calibrations in the “List of calibrations and their justifications” section in the Supplementary Materials). We calibrated the root to have a hard minimum of 574 Ma and a soft maximum of 609 Ma. The fossil minimum is based on the earliest occurrence of *Charnia masoni*, in the Drook Formation of Mistaken Point, Newfoundland (*56*), while the maximum is based on the Lantian biota of South China which contains a diversity of macrofossils but no credible metazoans (*57*). A uniform prior was used for this calibration, implying that we maintain an agnostic view of where the root of our phylogeny might lie between these constraints. The maximum on the root node is soft, with 2.5% of the probability density falling outside of the 574 to 609 Ma interval, on the deep side of the distribution. Eumetazoa was assigned a hard maximum of 573 Ma. This value was empirically defined to counter truncation problems in molecular clock analyses (see Materials and Methods for details) and a soft minimum of 561 Ma based on age of Bed B in the Bradgate Formation, where the earliest crown-eumetazoan, *Auroralumina attenboroughii*, was recovered. While it was possible to establish minimum constraints for the remaining sponge calibrations (*58*, *59*), it was not possible to justify maximum constraints based on paleontological, biogeographic, or geological evidence. The sponge fossil record, at least in terms of records that can inform calibrations for molecular clock analyses, is based on sites of exceptional fossil preservation. As such, we have no prior expectation of whether the earliest records of various sponge clades (details in the Supplementary Materials) represent close approximations of true clade ages. Cauchy distributions with a heavy-tail (see Materials and Methods and the Supplementary Materials) were used for these calibrations, reflecting lack of knowledge on which to establish maximum clade-age constraints.

Two calibration strategies were used. First, we performed analyses where the oldest known siliceous spicule was used to constrain the crown Heteroscleromorpha clade. We refer to this calibration strategy as the spiculate demosponges node (SDN). We then performed an analysis where the same fossil was used to calibrate the root of Porifera, spiculate Porifera node (SPN) strategy. These calibration strategies reflect competing interpretations of the fossil record of siliceous spicules. Under SDN, early siliceous fossil spicules are assumed to represent remains of spicule-bearing demosponges (crown Silicea), as suggested by the morphological similarity between the oldest classifiable siliceous spicules (*18*) and those found in living demosponges. Under SPN, siliceous spicules are assumed to have been present in the last common crown ancestor of Porifera, with the spicule fossil record providing a bound on the origin of sponges. We performed molecular clock analyses under the autocorrelated-rates [AR (*60*)] relaxed-clock model and the independent-rates [IR (*61*)] log-normal relaxed-clock model to assess the impact of both calibration strategies (SDN and SPN); see Materials and Methods and the Supplementary Materials for details. For each analysis (i.e., SDN-AR, SDN-IR, SPN-AR, and SPN-IR), we ran six independent chains, sampling from both the prior and the posterior. We checked for convergence (using in house developed scripts see Materials and Methods, Supplementary Methods, and https://doi.org/10.5281/zenodo.11488993 for details) and used the chains that passed our MCMC diagnostics to estimate divergence times (see figs. S4 to S6 and tables S1 and S2); see Materials and Methods and Supplementary Methods for details. Under SDN, we kept three of the six chains we ran under both the AR and IR relaxed-clock models (table S1). Under SPN, we retained four chains out of six for the AR model and five of six for the IR model (table S2).

Results inferred using the two relaxed-clock models and different calibration strategies did not differ significantly; we therefore focus on the results obtained with the SDN-IR analysis. Results from all other analyses are presented in the Supplementary Materials (figs. S7 to S9 and tables S3 and S4). Our SDN-IR analysis inferred the last common crown metazoan ancestor to have emerged 616.9 to 605.9 Ma (early Ediacaran); see Table 1 for a summary of the age inferred for key nodes across all our analyses. Similarly, the sponge last common crown ancestor was dated to 615.3 to 601.3 Ma (early Ediacaran). Crown-group Silicea (Hexactinellida + Demospongiae) was estimated at 591.3 to 570.3 Ma (middle Ediacaran), Lyssacinosida (within Hexactinellida) at 457.3 to 445 Ma (Late Ordovician), and the last common crown ancestor of Calcarea and Homoscleromorpha at 566.5 to 487.6 Ma (late Ediacaran to late Cambrian). Homoscleromorpha was estimated to have originated 429.1 to 228.6 Ma (middle Silurian, Late Triassic), and Calcarea 351.7 to 213.89 Ma (early Carboniferous, Late Triassic). The last common crown demosponge ancestor is estimated at 559.17 to 536.53 Ma (late Ediacaran, early Cambrian), and the last common crown ancestor of the aspiculate Verongimorpha and Keratosa 521.85 to 430.44 Ma (early Cambrian, middle Silurian). The last common crown ancestor of the spicule-bearing demosponges (Heteroscleromorpha) is estimated to have existed between 521.97 and 515.05 Ma (middle Cambrian), and the last common crown ancestor of the Haplosclerida was inferred at 403.84 to 246.54 Ma (Early Devonian, Middle Triassic). The last common crown ancestor of the rest of the Heteroscleromorpha was inferred to have existed 503.17 to 412.61 Ma (middle Cambrian, Early Devonian). Within Heteroscleromorpha, the freshwater Spongillida separated from the marine Scopalinida 424.69 to 325.85 Ma (late Silurian, late Carboniferous), while the heteroscleromorph lineages (Tetractinellida, Poecilosclerida, and Spongillida) diverged, respectively, 299.37 to 231.07 (early Permian, Late Triassic), 236.53 to 199.74 Ma (Late Triassic, Early Jurassic), and 123.21 to 71.52 Ma (Cretaceous).

**Table 1.** Divergence times for key nodes. Summary of posterior mean time estimates under both calibration strategies and molecular clock models for key nodes in our timetree. Full details can be found in tables S3 and S4.

Nodes	SDN AR 95% CIs for time estimates	SPN AR 95% CIs for time estimates	SDN IR 95% CIs for time estimates	SDN IR 95% CIs for time estimates
Porifera	603.79–582.68	594.59–578.79	615.32–601.3	610.68–590.34
Silicea	577.48–561.72	567.69–551.55	591.32–570.3	575.87–542.68
Demospongiae	549.57–538.18	535.02–503.38	559.17–536.53	519.67–462.67
Hexactinellida	454.92–445.07	443.89–422.5	457.3–445.09	454.99–445.07
Calcarea	473.24–163.27	460.38–157.52	351.7–213.89	337.36–207.53
Homoscleromorpha	540.28–435.36	535.63–420.18	429.15–228.57	421.58–224.87
Heteroscleromorpha	521.97–515.05	500.69–455.96	520.81–515.04	454.95–396.53

Phylogenetic relationships at the root of the metazoan tree are still debated (*2*–*8*). While evidence based on chromosome fusions was suggested to resolve Ctenophora as the sister of all the other animals (*5*), it has been shown that it is not possible to test whether the syntenic similarities underpinning the conclusions of (*5*) result from common descent or convergence (*6*). At the same time, the most recent phylogenomic study on this topic has found strong support for Porifera as the sister of all the other animals (*8*). Accordingly, for our main molecular clock analysis, we used a topology where sponges represent the sister of all the other animals. However, we acknowledge a lack of consensus on early animal relationships (*2*–*8*) and tested whether using a tree where ctenophores represent the sister of all the other animals influenced the results of our molecular clock analyses (figs. S10 and S11 and tables S5 and S6). These analyses showed that the relative relationships of ctenophores and sponges do not affect the timescale of sponge evolution [cf. (*22*)], as the credibility intervals for the divergence times of all key nodes across the two competing timescales overlap. When ctenophores are the first branching animal phyla, the age of the sponge last common crown-ancestor is estimated to fall in the interval 616 to 588 Ma, which includes the interval inferred when Porifera represents the sister of all the other animal phyla (615.3 to 601.3 Ma).

### Ancestral state reconstruction of skeletal elements

#### Incorporation of fossils in the phylogenetic framework

To understand the evolutionary history of spicules, we considered, in addition to the extant taxa in our phylogenomic dataset, all fossil taxa for which phylogenetic relationships have been tested using modern computational tree reconstruction approaches. Fossils were included to test the extent to which the inclusion of fossil spicules with morphological features not found in extant sponges might have affected our ancestral state reconstructions. The phylogenetic relationships of most fossil sponges have never been investigated using computational phylogenetic methods, preventing their inclusion in the backbone phylogenies necessary to anchor ancestral state estimation analyses. However, the recent description of *H. cantori* (*42*) was accompanied by the first formal morphological phylogenetic analysis of sponges including extant and fossil taxa. The fossil sampling in (*42*) is undoubtedly limited (it only includes seven fossil species). However, it includes the full disparity of mineralogical types observed in the early fossil record of sponges, as it includes the biminerallic *Eiffelia* (*62*) and *Protospongia* (*63*), the siliceous *Cyathophycus*, the aspiculate *H. cantori* (*42*), and *Vauxia* a Cambrian sponge initially described as a nonmineralized demosponge (*64*) that has since been shown to possess silicified fibers (*42*, *45*, *65*), if not spicules (*66*), and might be a stem Silicea (*42*) rather than a demosponge, but see (*67*) for a different opinion. Accordingly, the dataset of (*42*) is sufficient to provide us with a means to test the potential effects of including sponges with morphological features not observed across extant taxa on our ancestral state estimations. We first reanalyzed the dataset of (*42*) to confirm the placement of the fossils it includes, with reference to the taxa in our phylogenomic dataset. To achieve this goal, we performed new analysis of the morphological datasets of (*42*), where partial constraints were used to ensure that extant taxa were recovered following their placement in Fig. 1, while fossil placements where informed by the morphological data. Results of our analyses only differed from those of (*42*); see their extended data figure 5b, because we recovered *Vauxia* in a polytomy at the base of Porifera, rather than as a stem silicean (*42*) (see fig. S12B). To take into consideration the minor instability in the placement of *Vauxia* (fig. S12, A and B), we performed ancestral state reconstruction using two topologies: The topology in the extended data figure 5b in (*42*), see also fig. S12A, and the one recovered in our analyses (fig. S12B).

#### Ancestral state estimations

**Fig. 2.**
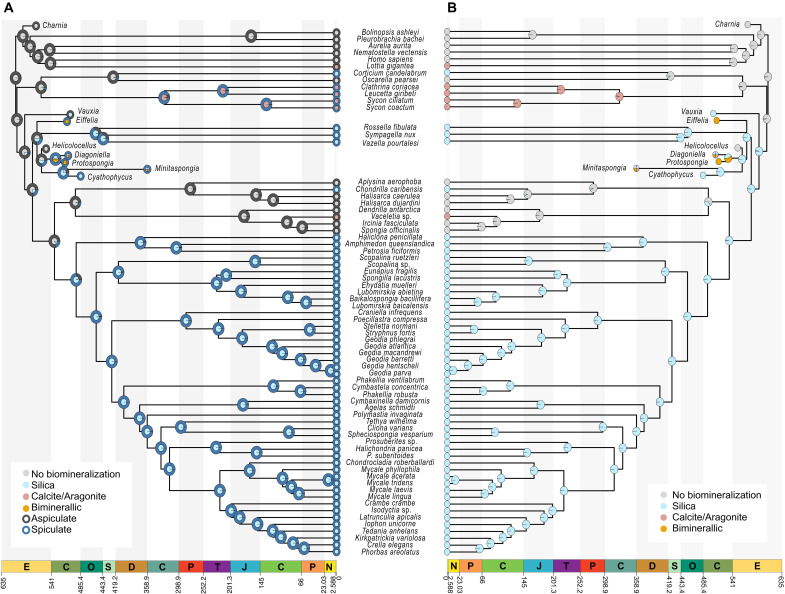
Early sponges did not have biomineralized skeletons. Ancestral state estimations of sponge skeletal elements obtained using the tree topology from (*42*) (their extended data figure 5b), see fig. S12A, dated using the timetree inferred using the SDN calibration strategy and the structured Markov model combining the presence/absence of spicules and their mineralogy (model D). Solid gray in the central circles at the nodes indicates no biomineralization. Light blue siliceous biomineralization, pink calcareous biomineralization, and orange biminerallic (both calcareous and siliceous) biomineralization. Outside circles represent the presence (blue) or absence (dark gray) of spicules. (**A**) Ancestral state reconstructions inferred under model D, which code both alternative biosilicification pathways and the presence and absence of spicules. (**B**) Ancestral state reconstructions inferred under model A, which only reconstruct whether ancestral sponges were capable of biomineralization and does not take into account alternative biosilicification pathways. In (A) (model D), all fossils are coded as unknown for the biosilicification pathway.

We performed three sets of ancestral state estimations using structured Markov models with embedded dependencies (*46*). First, we performed ancestral state reconstructions using the topology in Fig. 1. These analyses did not include fossils. We then performed reconstructions adding the fossils in fig. S12, arranged as in fig. S12A (Fig. 2) and fig. S12B (see fig. S13); see Materials and Methods and Supplementary Methods for details. The results of our analyses suggest that the last common crown-ancestors of Porifera, as well as the last common crown-ancestor of Calcarea plus Homoscleromorpha, were not biomineralized (*P* ≥ 0.92 and *P* ≥ 0.86 respectively; Fig. 2, A and B; fig. S13; and data S1). When *Vauxia* is placed in a polytomy (fig. S12B) and biosilicification is coded ignoring differences in silicification pathway (models A and B), the results still support this conclusion, although with greater ambiguity (*P* ≥ 0.53 and *P* ≥ 0.62, respectively; fig. S13). The last common ancestor of Silicea and Demospongiae emerge as not having been biomineralized when models are used that distinguish between different biosilicification pathways (models C and D; Fig. 2A and fig. S13). Differently, when biosilicification is coded ignoring differences in silicification pathway (models A and B), the last common ancestor of Silicea and Demospongiae emerge to be silicified (Fig. 2B and fig. S13). However, when biosilicification is coded ignoring differences in silicification pathway and the presence or absence of spicules is treated as an independently modeled trait (model B, fig. S13), we find the last common Silicea and Demospongiae ancestors as biomineralized but aspiculate, similarly to how *Vauxia* has been reconstructed in (*45*). Irrespective of when biosilicification first emerged all our results suggest that spicules were absent in the last common crown-ancestors of sponges (*P* ≥ 0.815) and of Calcarea plus Homosleromorpha (*P* ≥ 0.81). In analyses that included fossils (e.g., Fig. 2), the probability that the last common crown-ancestor of Porifera lacked spicules increases to *P* ≥ 0.975. Together, these results suggest that biosilicified spicules evolved independently multiple times, and that early sponges did not have them. Our analyses also strongly support multiple independent origins of biocalcification. Among the extant taxa in our dataset, biocalcification has evolved once in the stem-lineage of Calcarea and independently in the lineage leading to the demosponge genus *Vaceletia*. When fossil taxa are included, biocalcification is also inferred to have independently evolved in the biminerallic *Eifellia* and in the last common ancestor of the biminerallic *Protospongia* and *Diagoniella* (Fig. 2 and fig. S13). All the results of our ancestral state estimations are presented in a separate supplementary result file (data S1). They are also available in our Figshare repository (https://doi.org/10.6084/m9.figshare.28574570).

### The diversification of Silicea

We tested whether the origin of silicified spicules could have driven adaptive radiations in sponge evolution, performing a case study where we inferred diversification rate shifts in Silicea, the most biodiverse extant sponge lineage. We followed (*68*) and first generated a phylogeny of Silicea performing a constrained ML analysis of 807 *cytochrome c oxidase 1* (COI) barcodes, using our phylogenomic tree (Fig. 1 and fig. S1) to constrain the backbone topology (see Materials and Methods and Supplementary Methods for details). This resulted in a “megaphylogeny” (i.e., a species-rich tree) that was then dated (fig. S14 and table S7; see Materials and Methods and Supplementary Methods for details) and used to perform diversification rate shift analyses using Bayesian Analysis of Macroevolutionary Mixtures (BAMM) (*68*) (see table S8 for convergence statistics) and Medusa (*69*); see Materials and Methods and Supplementary Methods for details of how BAMM and Medusa were implemented.

**Fig. 3.**
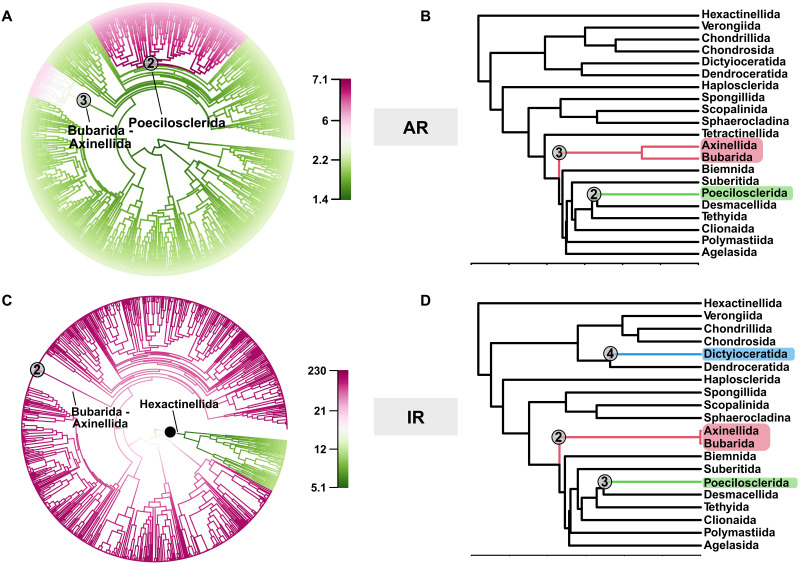
Diversifications events in Silicea do not correlate with spicule evolution. Rate diversification tests. Gray dots with numbers correspond to the shift highlighted by both Medusa and BAMM analyses, the same number is reported when the same shift corresponds on both topologies. (**A**) BAMM results for the COI timetree inferred with the AR model. The two shifts highlighted are on the branches of Bubarida + Axinellida (*3*) and Poecilosclerida (*2*). (**B**) Medusa results for the subsampled topology obtained with the AR molecular clock model. (**C**) BAMM results for the COI timetree inferred with the IR molecular clock model. The two shifts highlighted are on the branches of Bubarida + Axinellida (*2*) and Hexacintellida (black full dot). (**D**) Medusa results for the subsampled topology obtained with the IR molecular clock model, which highlights diversification rates in Axinellida + Bubarida (*2*), Poecilosclerida (*3*), and Dyctioceratida (*4*).

Only one diversification rate shift event was consistently identified by both BAMM and Medusa irrespective of the tree topology used. This shift is placed on the branch leading to the Axinellida plus Bubarida clade (Fig. 3, A to D). A second, putative diversification shift was identified on the branch leading to Poecilosclerida by all Medusa analyses (Fig. 3, B and D) and by one BAMM analysis (BAMM + AR tree; Fig. 3A). Other putative shifts were identified only by individual analyses. One putative shift leading to Hexactinellida was identified by BAMM but only on the IR tree (Fig. 3C), while Medusa identified a putative diversification shifts on the branch leading to Dictyoceratida when using the IR tree (Fig. 3D).

## DISCUSSION

### The sponge phylogeny is robustly resolved and rooted to the early Ediacaran

The results of our phylogenomic analyses confirm that sponges are split into two fundamental lineages, Calcarea plus Homoscleromorpha and Demospongiae plus Hexactinellida (Silicea). Consistent with other phylogenomic and mitogenomic studies (*7*, *8*, *70*), we recovered the monophyly of Keratosa, Verongimorpha, and Heteroscleromorpha within Demospongiae. We resolved the aspiculate Keratosa and Verongimorpha as sister clades, and the spiculate Heteroscleromorpha as sister to Keratosa plus Verongimorpha, corroborating previous findings (*69*). The relationships of Bubarida have been a long-standing problem in Demospongiae phylogeny, but our tree recovers Bubarida as the sister of Agelasida + Polymastiida + Tethyida + Clionaida + Suberitida + Poecilosclerida, similar to results recently obtained with mitochondrial genomes (*70*). However, we could not resolve with clear support the relationships of Suberitida with reference to Clionaida, Tethyida, and Poecilosclerida (figs. S1 to S3). All our timetrees recover an Ediacaran origin of sponges (615 to 579 Ma) while rejecting an Ediacaran origin for the spiculate demosponges (Heteroscleromorpha; 522 to 397 Ma), which according to our results cannot be older than Cambrian. While taxon sampling does not allow us to date the origin of Hexactinellida, our results are consistent with recent evidence for a putative crown hexactinellid (*H. cantori*) in the latest Ediacaran [539 Ma (*42*)].

### Early sponges did not have skeletal elements made of silica

Our ancestral character state estimations combined the taxa in our phylogenomic dataset and the fossils in (*42*). All our ancestral state estimate analyses reconstruct the poriferan crown-ancestor and the last common crown-ancestor of Calcarea plus Homoscleromorpha, as not biomineralized and aspiculate (*P* > 0.75). The condition of the silicean crown-ancestor is more uncertain, because of fossil instability (fig. S12) and the fact that for this node, ancestral character state reconstructions are affected by the way in which the embedded dependencies among states are defined (fig. S13). Nonetheless, all our analyses estimate the Silicea crown-ancestor as aspiculate (*P* > 0.643), even if the precise probability of this result depends on which tree topology was used to represent the relationships of the fossil taxa (fig. S13). In contrast, the ancestral state estimate of whether the silicean crown-ancestor was capable of biomineralization, depended on tree topology and model combination, with the silicean crown-ancestor emerging as capable of siliceous biomineralization when the tree of (*42*) and models A and B are used. In light of interpretations of *Vauxia* (*44*) has having had silicified fibers but no spicules, we suggest that the results from models A and B could be interpreted to suggest that the last common crown-ancestor of Silicea might have been armed with biosilicified fibers but did not have spicules.

If we consider only extant taxa, independent origin of siliceous spicules is suggested in stem-Hexactinellida, Homoscleromorpha (in stem-Plakinidae), in Demospongiae (in stem-Heteroscleromorpha), and the verongimorph *Chondrilla*. If we count also evolutionary events that happened in extinct lineages, other independent origins of siliceous spicules are inferred. However, these numbers are dependent on our fossil taxon sampling and should not be considered to represent precise estimates of the total number of independent origins of siliceous spicules (the estimation of which is not a goal of our study). Nonetheless, the multiple origin of siliceous spicules is consistent with current interpretations of spicule evolution based on comparative genomics and biochemistry, where developmental and biochemical differences have been interpreted as evidence for the convergent origin of these skeletal structures (*27*, *29*, *30*, *71*), a conclusion that is supported in our study.

### Early sponges did not have a calcareous skeleton

Understanding the evolution of calcareous skeletons is more complex. This is because the diversity of calcareous skeletal elements observed across Porifera and their patchy distribution, makes it difficult to decide whether they should be homologized when preparing morphological datasets for ancestral character state estimations. Given that our study was concerned with testing whether the last common crown-ancestor of sponges had skeletal structures, we tested whether early sponges might have had calcareous skeletal elements, irrespective of what these elements were. To achieve our goal, we coded all calcareous skeletal elements found across sponges as homologous. This is a valid hypothesis given that in Metazoa (including sponges), biocalcification is universally reliant on carbonic anhydrase paralogs, which might indicate a homologous origin of biocalcification followed by a diversification process that led to the emergence of significant biochemical and structural differences. However, all our ancestral state reconstructions conclude that calcareous skeletal elements evolved independently in different sponge lineages and were not present in the poriferan crown-ancestor. Our analyses therefore reject the hypothesis that calcareous skeletal elements in sponges might be homologous and that they were present in the crown-ancestor of all sponges.

### Early sponges had low fossilization potential

Given the results of our ancestral state estimation analyses, we conclude that latest Ediacaran siliceous spicules were most likely produced by sponges belonging to crown group Silicea, postdating the origin of crown sponges. Given that stem- and early crown-sponges lacked siliceous spicules and other mineralized skeletal elements, they would have been unlikely to leave fossil remains (*14*). Inevitably, our conclusions are dependent on the sampling of living and fossil sponges included in our ancestral state estimation analyses and our knowledge of their skeletal mineralogy. However, we have attempted to control for this uncertainty by including all fossil sponges that have, to date, been included in a phylogeny inferred using computational methods. Our test suggests that inclusion of fossils does not affect the way in which the crown-ancestor of Porifera is reconstructed but might have an impact on how more recent nodes (e.g., the last common crown ancestor of Silicea) are reconstructed. We recognize that, to date, only a few fossil sponges had their phylogenetic relationships tested using computational phylogenetic methods (*42*), and future studies using more fossils might reach different conclusions. Yet, our results are consistent with the view, based on comparative genomic evidence, that the different biochemical pathways used by sponges to produce siliceous spicules represent evidence that the homoscleromorph, hexactinellid, and demosponge siliceous spicules are not homologous (*27*, *29*, *30*, *71*). In detail, demosponges use silicatein enzymes (*28*, *29*, *72*, *73*) and hexactinellids use glassin, perisilin, and hexaxilin with the organic part of the spicule (the axial filament) having different compositions (*27*, *30*, *74*). Knowledge of the enzymes used by Homoscleromorpha is missing, but homoscleromorphs lack key biosilicification genes found in Hexactinellida and Demospongiae, pointing to a different developmental pathway (*29*, *75*).

### The emergence of silicified spicules did not drive the diversification of sponges

Sponges are globally distributed and comprise ca. 9700 species (*76*). In terms of biodiversity, Demospongiae has undoubtedly been the most successful sponge lineage (*76*). Furthermore, they have been ecologically more successful, for instance, being able to transition to freshwater and undergo revolutionary body plan rearrangements, leading to the only known case of carnivory in the phylum (*77*). Many explanations have been proposed to explain their success. One is that the high diversity of spicule shapes would have been advantageous for structural functions and defense, driving adaptive radiations (*78*); the results of our diversification analyses suggest otherwise. While spicules certainly play a role in defense and structural support, their emergence does not appear to have underpinned adaptive radiations, evidence of which should be shown as a diversification event on the branch that leads to the radiation of Heteroscleromorpha. Instead, the only diversification event consistently recovered across all our analyses is on the branch that leads to Bubarida and Axinellida (Fig. 3). Both groups are nested well within Heteroscleromorpha, and spicules were not an innovation specific to them. Furthermore, their spicule types are shared across many other demosponge orders (table S9) where we did not identify diversification events. Analyses that used Medusa highlighted also a putative diversification event in stem-Poecilosclerida, which is however only confirmed by one BAMM analysis and is therefore less certain. Poecilosclerida represents the most speciose demosponge order with ca. 2488 species described to date (*76*), with a worldwide distribution and substantial ecological diversity. However, Poecilosclerida are also members of the spiculate lineage and did not evolve spicules independently, although they do present one of the largest spicule complements in Porifera. We therefore conclude that while the poecilosclerid evolutionary success might have been linked to the massive expansion of microscleres observed in this clade (*79*, *80*), the origin of spicules itself cannot have driven their diversification.

### Reconciling the fossil record of sponges with molecular estimates of divergence times

Previous evolutionary timelines based on molecular data have inferred sponges to have originated in the Cryogenian (*10*, *12*, *13*, *81*). Differently, our evolutionary timescale infers crown-group sponges to be significantly younger, emerging in the early-middle Ediacaran (617.91 to 581.39 Ma). Our newly estimated, and younger, clade ages are driven by both a revised geochronology and reinterpretation of the fossil evidence used in establishing calibrations to be used in Bayesian node-dating analyses (*22*). There are a number of speculative interpretations of fossil sponges from the Tonian and Cryogenian but none of these are credible (*14*, *15*). In particular, steranes (24-ipc and 26-mes) found from the late Cryogenian (660 to 635 Ma) to the Early Cambrian have had a long history of alternative interpretations (*9*, *11*, *82*–*84*), and many of these might represent diagenetic derivatives of C29 sterols from algae (*18*). Newly identified derivatives of C31 hydrocarbons, 24-secbc and 24-nbc, are more promising in that their co-occurrence with 24-ipc and 26-mes in Ediacaran strata might provide corroborative evidence of their generation from demosponge-derived sterols, rather than algae (*85*). However, their interpretation as demosponge or even sponge-specific remains insufficiently robust to calibrate molecular clock analyses.

Understanding the siliceous spicule fossil record and reconciling its history against the timescale of sponge evolution is key to elucidating sponge origins. Silica is chemically stable under low-temperature and high-pressure conditions (*14*). If sponges bearing siliceous spicules existed in the Neoproterozoic, they should have been preserved, given also that the Neoproterozoic ocean geochemistry was conductive to the preservation of silica, as confirmed by the presence of abundant cherts in the late Ediacaran (*86*, *87*). The probability that sponges bearing siliceous spicules existed significantly earlier than the oldest known records (ca. 543 Ma) but were not preserved is therefore low (*14*, *25*, *26*). However, the interpretation of the siliceous spicule record is difficult. The oldest siliceous spicules could be interpreted to represent evidence for spicule-bearing stem sponges, in which case the siliceous spicule record would place a limit on the age of crown-sponges, which would be unlikely to be much older than the oldest record of fossil spicules (*14*). However, these spicules can also be interpreted to represent Neoproterozoic evidence for one of the crown lineages bearing siliceous spicules: i.e., Silicea or Homoscleromorpha [although the latter is unlikely (*14*), in agreement with our molecular clock results, Fig. 1]. Under this second interpretation, the oldest spicule record would not be representative of the age of the oldest sponges. If the ancestors of these spicule-bearing crown sponges were aspiculate and not capable of biomineralizing, they would have had low fossilization potential and would have been unlikely to leave a fossil record (*26*). Under this second hypothesis, Porifera could have enjoyed a long, cryptic, Neoproterozoic history, potentially extending to the Cryogenian or the Tonian.

Our analyses did not use Cryogenian-to-Cambrian fossil biomarkers to calibrate molecular clock analyses. However, our results still indicate that total-group sponges have a Neoproterozoic history, albeit extending only to the early-middle Ediacaran (617.91 to 581.39 Ma), that is unrepresented in the fossil record. Our study shorten the gap between the earliest unambiguous fossil evidence for sponges and molecular divergence times from ca. 150 Myr to 38 to 75 Myr but continue to support a cryptic phase in the early history of Porifera. Our ancestral character state estimates indicate that stem sponges were aspiculate and uncapable of biomineralization. This allows us to rationalize the Neoproterozoic gap between our molecular estimate for the origin of sponges and the siliceous spicule fossil record as a consequence of the low preservation probability of aspiculate, nonbiomineralized, stem- and early crown-sponges.

In accordance with the fossil record, our results suggest that demosponge spicules emerged in ca. 31 Myr interval between 548 and 517 Ma (Fig. 1). In Homoscleromorpha, silicified spicules were only acquired by the Plakinidae, after the divergence from Oscarellidae in the Carboniferous. Inadequate taxon sampling prevents us from defining precisely the period during which spicules emerged in Hexactinellida. It could be tempting to interpret the aspiculate *H. cantori* to inform a maximum bound on the evolution of spicules in Hexactinellida. However, the diversity of skeletal structures observed in stem hexactinellids (Fig. 2) and the relationships of *H. cantori* within total group Hexactinellida (Fig. 2) suggest that using the age of *H. cantori* as a maximum bound on the origin of siliceous spicules in Hexactinellida would be unwarranted.

Phylogenetic bracketing implies that hexactinellid spicules cannot be older than the silicean crown-ancestor (577.48 to 561.72 Ma), from which both the Hexactinellida and Demospongiae total-groups emerged. However, siliceous spicules emerged relatively late in demosponges (Fig. 2), potentially suggesting that Hexactinellida might have been the first, among the lineages of extant sponges, to evolve spicules.

Our results explain the existence of an early siliceous spicule fossil record without the need to assume that stem sponges synthesized siliceous spicules. Since sponges branch early within the animal phylogeny [irrespective of the controversy on the relative relationships between sponges and ctenophores (*2*–*8*)], they are informative of early metazoan evolution, and the origin of animal multicellularity. Advocates of a literal reading of the fossil record would suggest that the origin of sponges (and Metazoa) corresponds closely with the first appearance of siliceous spicules in the fossil record [ca. 543 Ma; latest Ediacaran (*15*)]. Our divergence time analyses reduce by more than half the perceived gap between the earliest fossil spicules and molecular estimates for the origin of sponges. This gap might diminish even further if older, unambiguous, sponge fossils are discovered. While we agree on the importance of fossils for inferring character evolution and resolving phylogenetic controversies (*88*, *89*), our study demonstrates that the power of the fossil record in inferring evolutionary history is predicated on restoring extinct species to the rightful place within the phylogenies of living taxa. Only with an integrated understanding of phylogeny and character evolution we can hope to achieve an understanding of the evolutionary assembly of animal body plans (*90*).

## MATERIALS AND METHODS

### Sample collection, RNA extraction, library preparation, and Illumina sequencing

Twelve novel sponge specimens were collected for RNA extraction (table S10). Samples were preserved in RNAlater (Thermo Fisher Scientific, Waltham, USA) using an overnight incubation at 4°C before long-term storage at −80°C. Total RNA was extracted with TRIzol (Ambion, Austin, USA) and mRNA purified with a Dynabeads mRNA DIRECT kit (Thermo Fisher Scientific, Waltham, USA). The cDNA libraries were constructed with either the kits TruSeq version 2 or Scriptseq version 2 RNA Library Prep kit (Illumina), according to the manufacturer’s instructions, using the maximum quantity of mRNA allowed, which was 50 to 100 ng of starting material. The quality of the libraries was checked with an Agilent TapeStation 2200 system (Agilent Technologies) and the quantity with Qubit dsDNA HS Assay kit (Thermo Fisher Scientific). The sequencing was conducted with an Illumina NextSeq 500 platform at the Sequencing Facilities of the Natural History Museum of London (Research Core Labs), using a paired-end read strategy (bp length, 2 × 150 bp).

### Assembly of transcriptomic data and preparation of the phylogenomic dataset

In addition to the 12 novel transcriptomes, 58 published RNA sequencing (RNA-seq) datasets with sequence read archive IDs were included. All data were translated to proteins with the final total dataset composed of 70 proteomes (table S10). See Supplementary Methods for details about the software and settings used in the analyses.

Orthologs were inferred using an Orthofinder (*50*) based pipeline; see Supplementary Methods for details about the software and settings used in the pipeline. The final dataset generated in this way was composed of 133 genes that were analyzed independently to reconstruct gene trees in IQTree (*51*) and concatenated in a super-alignment of 103,296 AA using the catfasta2phyml.pl script from https://github.com/nylander/catfasta2phyml.

### Phylogenetic analyses

The 103,296 AA superalignment was analyzed using ML in IQTree2 version 2.1.3 (*91*). Model testing was performed using the BIC in IQTree2, considering only across-site compositionally homogeneous models. Model selection identified the Q.INSECT+I+G4 model as best fit. We used the ultrafast bootstrap (1000 replicates) to estimate support for the nodes in the ML tree. Bayesian analyses were performed using Phylobayes (*53*) under the across-site compositionally heterogeneous CAT-Poisson model (*52*). In these analyses support values are estimated using PPs. We ran two independent chains for over 7000 generations (Burnin = 1500). Convergence was tested using Bpcomp (maxdiff = 0.0018315) and Tracecomp (minimal ESS 61 and maximum real_diff = 0.224488). Bpcomp and Tracecomp are part of the Phylobayes distribution (see table S8 for full convergence scores). Last, we used ASTRAL (*5*) as a coalescent tree reconstruction method on the 133 gene trees generated from the ML analyses of the single gene alignments, performed in IQTree under their gene-specific best fitting model; see Supplementary Methods for details. Astral was used with default parameters.

### Inference of divergence times

We fixed the tree topology to our inferred Bayesian tree (Fig. 1 and fig. S1) and constrained the age of 12 nodes representing major sponge clades in our phylogeny (red dots and circles in Fig. 1) based on fossil evidence (see the “List of calibrations and their justifications” section in the Supplementary Materials). The oldest fossil spicule was initially used to calibrate the origin of the spicule-bearing Demospongiae (SDN calibration strategy). We used a uniform prior on the root age with a soft maximum of 609 Ma (pU = 0.025) and a hard minimum of 574 Ma (*p* = 1 × 10^−300^; this small value enables the usage of hard bounds in MCMCtree as pL = 0 would cause numerical underflow). We constrained the age of Eumetazoa with a hard maximum of 573 Ma (pU = 1 × 10^−300^) and a soft minimum of 561 Ma (pU = 0.025) to avoid truncation issues during the MCMC runs (*87*). The age of the other 10 calibrated nodes was constrained using heavy-tail Cauchy distributions (i.e., lower-bound calibrations with an offset of *p* = 0.1 or *p* = 0.5, a scale parameter of *c* = 0.5 or *c* = 0.1, and a left tail probability of pL = 1 × 10^−300^ to enforce a hard bound on the specified minimum age). The use of a heavy-tailed distribution was preferred as it allows us to capture the credibility of the sponge fossil record, which is unlikely to be reliable in deep time because of taphonomic processes having altered diagnostic characters, thus posing further difficulties on taxonomic classifications. We performed a sensitivity test (SPN strategy) where the oldest siliceous spicule was reassigned to calibrate the node representing the last common sponge ancestor (see also Fig. 1) to account for uncertainty in the interpretation of the spicule fossil record, allowing us to investigate its impact on poriferan divergence times derived from a molecular clock-dating analysis. In short, the SDN strategy constrained the spicule-bearing Demospongiae (Heteroscleromorpha) to have a hard minimum at 515 Ma, while the SPN strategy constrained the root of Porifera to a minimum age of 515 Ma. Both experiments used a Cauchy distribution specified in the same way (*c* = 0.5, *p* = 0.5, and pL = 1 × 10^−300^). The age of 515 Ma used in both the SDN and SPN strategies is based on loose siliceous spicules from the lower Cambrian (series 2, stage 3) Sirius Passet Biota of North Greenland (*92*). A list of all the calibrations used can be found in the Supplementary Materials, “List of calibrations and their justifications.”

To estimate species divergence times, we dated our inferred Bayesian phylogeny (Fig. 1 and fig. S1) using MCMCtree, which is part of the PAML package (*88*). See Supplementary Methods and https://doi.org/10.5281/zenodo.11488993 for details about the settings of all our molecular clock analyses.

To account for uncertainty on sponge divergence times that might be caused by phylogenetic uncertainty at the root of the animal tree of life, we ran a ML analysis constraining the topology to force Ctenophora to represent the sister group of all the other animals. We then calibrated the ML tree using the same calibrations used in our other molecular clock analyses and performed new divergence time analyses. We used our in-house pipeline for chains filtering and to generate convergence and summary plots (see above, the Supplementary Materials, and figs. S15 to S17).

There is some disagreement on the nature of *Geoditesia jordaniensis* (*93*). Accordingly, we performed a sensitivity analysis where we changed the calibration interval defined by this fossil (166.1 to 163.5 Ma), to 166.1 to 150 Ma, to take into consideration uncertainty on this part of the sponge fossil record. This analysis did not affect our results.

### Ancestral character state reconstructions

#### Assembling a phylogenetic framework with fossils and extant taxa

Sponges have a rich fossil record (*14*), which can provide additional evidence of spicule evolution. Fossil observations are particularly useful for ancestral state estimation, as their closer proximity to the root of the phylogenetic tree compared to living taxa gives them greater influence on ancestral likelihoods. Unfortunately, the majority of fossil sponges have never been included in a formal phylogenetic analysis, and their relationships are unknown. Accordingly, they cannot be included in ancestral state reconstruction analyses. However, the recent study of the relationships of *H. cantori* (*42*), included seven fossil sponges with a broad diversity of skeletal structure, from the aspiculate and nonbiomineralized *H. cantori*, to the biminerallic *Eiffelia* and *Protospongia*, and the silicified but likely aspiculate *Vauxia*. Accordingly, in addition to performing ancestral state reconstructions using the taxa in our phylogenomic dataset, we were also able to perform sensitivity tests on sponge phylogenies which included fossils. Because fossils can include combinations of states not observed in living taxa (e.g., sponges with biminerallic spicules), their inclusion can affect ancestral state reconstructions, and it is important to test the extent that this might affect our results. Our sensitivity tests provide a mean to test the uncertainty of our ancestral state estimations, and whether the inclusion of taxa with states not observed among extant taxa can affect ancestral reconstructions at internal nodes. To test the effect of fossils on our ancestral states analyses, we produced time trees from phylogenies integrating the results of our phylogenomic analysis with those of (*42*). Our main ancestral state reconstruction analysis that included fossils used the tree published by (*42*) in extended figure 5b. However, we tested the results of the analyses of (*42*) performing new partially constrained Bayesian analyses [in MrBayes 3.7 (*94*)] where we left the fossils free to arrange themselves in the optimal position in a backbone tree representing our phylogenomic results, that is, on a tree constraining the monophyly of Silicea (Hexactinellida plus Demospongiae) and that of Calcarea plus Homoscleromorpha. Because the results of our phylogenetic analyses were slightly different from those of (*42*), we performed sensitivity analyses also using the tree in fig. S12B, which represent the results of our reanalysis of the dataset of (*42*). Methodological details of these analyses are presented in Supplementary Methods. Input and result files for these analyses, which identified a placement of the fossil *Vauxia* which is slightly different from that of (*42*), can be found in our Figshare repository (https://doi.org/10.6084/m9.figshare.28574570).

#### Estimation of ancestral states

We performed marginal likelihood ancestral state estimation in R; see Supplementary Methods and https://doi.org/10.6084/m9.figshare.28574570 for details of packages and functions used. Character states for the full dataset were sourced from the literature (table S9). The matrix we used for the analyses can be downloaded from https://doi.org/10.6084/m9.figshare.28574570. To investigate the evolution of sponges with different types of skeletal structures, we used embedded dependency models (*45*, *95*) and accounted for underlying biochemical and developmental differences and similarities.

We coded the following characters:

1) Spicule presence: absent = 0 and present = 1

2) Calcareous biomineralization: absent = 0 and present = 1

3) Siliceous biomineralization: absent = 0 and present = 1

4) Siliceous biomineralization pathway: Hexactinellida = 0, Demospongiae = 1, and Homoscleromorpha = 2

For biocalcification, we did not differentiate developmental pathways, assuming a shared mechanism across taxa. This assumption reflects the widespread use of carbonic anhydrase paralogs in sponges to form carbonate-based skeletal elements, including spicules (*31*, *32*, *37*, *38*). The use of carbonic anhydrase paralogs can be considered a shared similarity that could underpin a relationship of homology across all calcified skeletal elements in sponges. It is important to stress that we are aware of the significant differences in biocalcification pathways across sponges (*32*) and we do not propose that biocalcified skeletal elements found across Porifera are homologous. We simply defined a putative homology across these structures (a hypothesis of homology) that we then tested using ancestral character estimation.

Characters (2) and (3) were treated as independent to accommodate fossil taxa with biminerallic spicules [e.g., *Eiffelia* and *Protospongia* (*41*, *63*)]. Character (1) was also treated as independent from (2) and (3), allowing for biomineralizing taxa that lack spicules [e.g., *Vaceletia* and *Vauxia* (*33*)]. Character (4) was treated as dependent on character (3), as it reflects alternative biochemical pathways for silica deposition. We note that not all our analyses used a coding reflecting alternative biochemical pathways. In the models that we refer to as models A and B (see Supplementary Methods for details), biosilicification is coded ignoring differences in silicification pathway. Model A does not model the evolution of spicules, focusing exclusively on whether common sponge ancestors were capable of biosilicification. In model B, the presence and absence of spicules is modeled using an independent (unlinked) binary character. Models C and D define different biosilicification pathways based on biochemical evidence (this is done using two hierarchically nested characters). In model C, similarly to model A, presence absence of spicules is not modeled. In model D, similarly to model B, presence absence of spicules is modelled with an independent binary character. In models C and D, all fossils with siliceous biomineralization were coded as unknown for the specific pathway used to excrete silica.

See Supplementary Methods for details about how we amalgamated the rate matrices of independent characters using structured Markov models (*46*) and dependent characters using embedded dependencies (*47*). In total, we conducted 432 ancestral state estimations. Results and scripts can be found in our Figshare repository (https://doi.org/10.6084/m9.figshare.28574570).

### Silicean diversification rates through time

Macroevolutionary dynamics of diversification was modelled using BAMM version 2.5.0 (*96*) and Medusa (*69*) on large, dated phylogenies (i.e., megaphylogenies) of the Silicea. We followed (*68*) and first generated a dated megaphylogeny of Silicea performing a constrained ML analysis of 807 COI barcodes, using our phylogenomic tree as a backbone constraint. We then dated the megaphylogeny of Silicea under the IR and AR molecular clock models using the calibrations in table S12. BAMM and Medusa were then applied on the dated megaphylogenies to infer diversification rate shifts; see Supplementary Methods for the settings of our BAMM and Medusa analyses.

## Supplementary Materials

### The PDF file includes:

Supplementary Text Figs. S1 to S18 Tables S1 to S13 Legend for data S1 References

## References

[1] J. R. Pawlik, S. E. McMurray, The emerging ecological and biogeochemical importance of sponges on coral reefs. Ann. Rev. Mar. Sci. 12, 315–337 (2020). 10.1146/annurev-marine-010419-010807 31226028

[2] C. W. Dunn, A. Hejnol, D. Q. Matus, K. Pang, W. E. Browne, S. A. Smith, E. Seaver, G. W. Rouse, M. Obst, G. D. Edgecombe, M. V. Sørensen, S. H. D. Haddock, A. Schmidt-Rhaesa, A. Okusu, R. M. Kristensen, W. C. Wheeler, M. Q. Martindale, G. Giribet, Broad phylogenomic sampling improves resolution of the animal tree of life. Nature 452, 745–749 (2008). 18322464 10.1038/nature06614

[3] D. Pisani, W. Pett, M. Dohrmann, R. Feuda, O. Rota-Stabelli, H. Philippe, N. Lartillot, G. Wörheide, Genomic data do not support comb jellies as the sister group to all other animals. Proc. Natl. Acad. Sci. U.S.A. 112, 15402–15407 (2015). 26621703 10.1073/pnas.1518127112 PMC4687580

[4] R. Feuda, M. Dohrmann, W. Pett, H. Philippe, O. Rota-Stabelli, N. Lartillot, G. Wörheide, D. Pisani, Improved modeling of compositional heterogeneity supports sponges as sister to all other animals. Curr. Biol. 27, 3864–3870.e4 (2017). 29199080 10.1016/j.cub.2017.11.008

[5] D. T. Schultz, S. H. D. Haddock, J. V. Bredeson, R. E. Green, O. Simakov, D. S. Rokhsar, Ancient gene linkages support ctenophores as sister to other animals. Nature 618, 110–117 (2023). 37198475 10.1038/s41586-023-05936-6 PMC10232365

[6] R. R. Copley, Sponges, ctenophores and the statistical significance of syntenies. Mol. Biol. and Evol., msaf321 (2025). 41355550 10.1093/molbev/msaf321 PMC12728501

[7] H. Philippe, R. Derelle, P. Lopez, K. Pick, C. Borchiellini, N. Boury-Esnault, J. Vacelet, E. Renard, E. Houliston, E. Quéinnec, C. Da Silva, P. Wincker, H. Le Guyader, S. Leys, D. J. Jackson, F. Schreiber, D. Erpenbeck, B. Morgenstern, G. Wörheide, M. Manuel, Phylogenomics revives traditional views on deep animal relationships. Curr. Biol. 19, 706–712 (2009). 19345102 10.1016/j.cub.2009.02.052

[8] J. L. Steenwyk, N. King, Integrative phylogenomics positions sponges at the root of the animal tree. Science 390, 751–756 (2025). 41232001 10.1126/science.adw9456

[9] D. A. Gold, J. Grabenstatter, A. De Mendoza, A. Riesgo, I. Ruiz-Trillo, R. E. Summons, Sterol and genomic analyses validate the sponge biomarker hypothesis. Proc. Natl. Acad. Sci. U.S.A. 113, 2684–2689 (2016). 26903629 10.1073/pnas.1512614113 PMC4790988

[10] E. A. Sperling, J. M. Robinson, D. Pisani, K. J. Peterson, Where’s the glass? Biomarkers, molecular clocks, and microRNAs suggest a 200-Myr missing Precambrian fossil record of siliceous sponge spicules: Sponge biomarkers, molecular clocks and microRNAs. Geobiology 8, 24–36 (2010). 19929965 10.1111/j.1472-4669.2009.00225.x

[11] J. A. Zumberge, G. D. Love, P. Cárdenas, E. A. Sperling, S. Gunasekera, M. Rohrssen, E. Grosjean, J. P. Grotzinger, R. E. Summons, Demosponge steroid biomarker 26-methylstigmastane provides evidence for Neoproterozoic animals. Nat. Ecol. Evol. 2, 1709–1714 (2018). 30323207 10.1038/s41559-018-0676-2 PMC6589438

[12] D. H. Erwin, M. Laflamme, S. M. Tweedt, E. A. Sperling, D. Pisani, K. J. Peterson, The Cambrian conundrum: Early divergence and later ecological success in the early history of animals. Science 334, 1091–1097 (2011). 22116879 10.1126/science.1206375

[13] M. Dohrmann, G. Wörheide, Dating early animal evolution using phylogenomic data. Sci. Rep. 7, 3599 (2017). 28620233 10.1038/s41598-017-03791-w PMC5472626

[14] J. P. Botting, L. A. Muir, Early sponge evolution: A review and phylogenetic framework. Palaeoworld 27, 1–29 (2018).

[15] J. B. Antcliffe, R. H. T. Callow, M. D. Brasier, Giving the early fossil record of sponges a squeeze. Biol. Rev. 89, 972–1004 (2014). 24779547 10.1111/brv.12090

[16] E. C. Turner, Possible poriferan body fossils in early Neoproterozoic microbial reefs. Nature 596, 87–91 (2021). 34321662 10.1038/s41586-021-03773-z PMC8338550

[17] F. Neuweiler, S. Kershaw, F. Boulvain, M. Matysik, C. Sendino, M. McMenamin, A. Munnecke, Keratose sponges in ancient carbonates – A problem of interpretation. Sedimentology 70, 927–968 (2023).

[18] I. Bobrovskiy, J. M. Hope, B. J. Nettersheim, J. K. Volkman, C. Hallmann, J. J. Brocks, Algal origin of sponge sterane biomarkers negates the oldest evidence for animals in the rock record. Nat. Ecol. Evol. 5, 165–168 (2021). 33230256 10.1038/s41559-020-01334-7

[19] A. C. Maloof, C. V. Rose, R. Beach, B. M. Samuels, C. C. Calmet, D. H. Erwin, G. R. Poirier, N. Yao, F. J. Simons, Possible animal-body fossils in pre-Marinoan limestones from South Australia. Nat. Geosci. 3, 653–659 (2010).

[20] Z. Yin, M. Zhu, E. H. Davidson, D. J. Bottjer, F. Zhao, P. Tafforeau, Sponge grade body fossil with cellular resolution dating 60 Myr before the Cambrian. Proc. Natl. Acad. Sci. U.S.A. 112, E1453–E1460 (2015). 25775601 10.1073/pnas.1414577112 PMC4378401

[21] J. A. Cunningham, A. G. Liu, S. Bengtson, P. C. J. Donoghue, The origin of animals: Can molecular clocks and the fossil record be reconciled? Bioessays 39, 1–12 (2017). 10.1002/bies.201600120 27918074

[22] E. Carlisle, Z. Yin, D. Pisani, P. C. J. Donoghue, Ediacaran origin and Ediacaran-Cambrian diversification of Metazoa. Sci. Adv. 10, eadp7161 (2024). 39536100 10.1126/sciadv.adp7161 PMC11559618

[23] M. Brasier, O. Green, G. Shields, Ediacarian sponge spicule clusters from southwestern Mongolia and the origins of the Cambrian fauna. Geology 25, 303–306 (1997).

[24] Y. Zhang, X. Yuan, L. Yin, Interpreting Late Precambrian microfossils. Science 282, 1783 (1998).

[25] S. Chang, Q. Feng, S. Clausen, L. Zhang, Sponge spicules from the lower Cambrian in the Yanjiahe Formation, South China: The earliest biomineralizing sponge record. Palaeogeogr. Palaeoclimatol. Palaeoecol. 474, 36–44 (2017).

[26] Q. Tang, B. Wan, X. Yuan, A. D. Muscente, S. Xiao, Spiculogenesis and biomineralization in early sponge animals. Nat. Commun. 10, 3348 (2019). 31350398 10.1038/s41467-019-11297-4 PMC6659672

[27] W. R. Francis, M. Eitel, S. Vargas, C. A. Garcia-Escudero, N. Conci, F. Deister, J. L. Mah, N. Guiglielmoni, S. Krebs, H. Blum, S. P. Leys, G. Wörheide, The genome of the reef-building glass sponge *Aphrocallistes vastus* provides insights into silica biomineralization. R. Soc. Open Sci. 10, 230423 (2023). 37351491 10.1098/rsos.230423 PMC10282587

[28] W. E. Müller, A. Krasko, G. Le Pennec, R. Steffen, M. Wiens, M. S. A. Ammar, I. M. Müller, H. C. Schröder, Molecular mechanism of spicule formation in the demosponge suberites domuncula: Silicatein-collagen-myotrophin. Prog. Mol. Subcell. Biol. 33, 195–221 (2003). 14518374 10.1007/978-3-642-55486-5_8

[29] A. Riesgo, M. Maldonado, S. López-Legentil, G. Giribet, A proposal for the evolution of cathepsin and silicatein in sponges. J. Mol. Evol. 80, 278–291 (2015). 25987356 10.1007/s00239-015-9682-z

[30] K. Shimizu, M. Nishi, Y. Sakate, H. Kawanami, T. Bito, J. Arima, L. Leria, M. Maldonado, Silica-associated proteins from hexactinellid sponges support an alternative evolutionary scenario for biomineralization in Porifera. Nat. Commun. 15, 181 (2024). 38185711 10.1038/s41467-023-44226-7 PMC10772126

[31] O. Voigt, M. V. Wilde, T. Fröhlich, B. Fradusco, S. Vargas, G. Wörheide, Genetic parallels in biomineralization of the calcareous sponge Sycon ciliatum and stony corals. eLife 14, RP106239 (2025). 40922549 10.7554/eLife.106239 PMC12419799

[32] O. Voigt, M. Adamska, M. Adamski, A. Kittelmann, L. Wencker, G. Wörheide, Spicule formation in calcareous sponges: Coordinated expression of biomineralization genes and spicule-type specific genes. Sci. Rep. 7, 45658 (2017). 28406140 10.1038/srep45658 PMC5390275

[33] G. Wörheide, A hypercalcified sponge with soft relatives: Vaceletia is a keratose demosponge. Mol. Phylogenet. Evol. 47, 433–438 (2008). 18321733 10.1016/j.ympev.2008.01.021

[34] J. Jeon, M. Simonet Roda, Z.-Y. Chen, C. Luo, S. Kershaw, D. Kim, J.-Y. Ma, J.-H. Lee, Y.-D. Zhang, Phosphatic stromatoporoid sponges formed reefs ~480 Mya. Proc. Natl. Acad. Sci. U.S.A. 122, e2426105122 (2025). 40163761 10.1073/pnas.2426105122 PMC12012458

[35] P. U. P. A. Gilbert, K. D. Bergmann, N. Boekelheide, S. Tambutté, T. Mass, F. Marin, J. F. Adkins, J. Erez, B. Gilbert, V. Knutson, M. Cantine, J. O. Hernández, A. H. Knoll, Biomineralization: Integrating mechanism and evolutionary history. Sci. Adv. 8, eabl9653 (2022). 35263127 10.1126/sciadv.abl9653 PMC8906573

[36] D. J. Jackson, L. Macis, J. Reitner, B. M. Degnan, G. Wörheide, Sponge paleogenomics reveals an ancient role for carbonic anhydrase in skeletogenesis. Science 316, 1893–1895 (2007). 17540861 10.1126/science.1141560

[37] O. Voigt, B. Fradusco, C. Gut, C. Kevrekidis, S. Vargas, G. Wörheide, Carbonic anhydrases: An ancient tool in calcareous sponge biomineralization. Front. Genet. 12, 624533 (2021). 33897759 10.3389/fgene.2021.624533 PMC8058475

[38] O. Voigt, M. Adamski, K. Sluzek, M. Adamska, Calcareous sponge genomes reveal complex evolution of α-carbonic anhydrases and two key biomineralization enzymes. BMC Evol. Biol. 14, 230 (2014). 25421146 10.1186/s12862-014-0230-z PMC4265532

[39] J. Germer, K. Mann, G. Wörheide, D. J. Jackson, The skeleton forming proteome of an early branching metazoan: A molecular survey of the biomineralization components employed by the coralline sponge Vaceletia Sp. PLOS ONE 10, e0140100 (2015). 26536128 10.1371/journal.pone.0140100 PMC4633127

[40] S. M. Rowland, Archaeocyaths—A history of phylogenetic interpretation. J. Paleo. 75, 1065–1078 (2001).

[41] J. P. Botting, N. J. Butterfield, Reconstructing early sponge relationships by using the Burgess Shale fossil Eiffelia globosa, Walcott. Proc. Natl. Acad. Sci. U.S.A. 102, 1554–1559 (2005). 15665105 10.1073/pnas.0405867102 PMC547825

[42] X. Wang, A. G. Liu, Z. Chen, C. Wu, Y. Liu, B. Wan, K. Pang, C. Zhou, X. Yuan, S. Xiao, A late-Ediacaran crown-group sponge animal. Nature 630, 905–911 (2024). 38839967 10.1038/s41586-024-07520-y

[43] J. P. Botting, D. Janussen, M. Dohrmann, L. A. Muir, Y. Zhang, J. Ma, Advanced crown-group Rossellidae (Porifera: Hexactinellida) resembling extant taxa from the Hirnantian (Late Ordovician) Anji Biota. Pap. Palaeontol. 11, e70000 (2025).

[44] B. Runnegar, J. G. Gehling, S. Jensen, M. R. Saltzman, Ediacaran paleobiology and biostratigraphy of the Nama Group, Namibia, with emphasis on the erniettomorphs, tubular and trace fossils, and a new sponge, *Arimasia* germsi n. gen. n. sp. J. Paleontol. 98, 1–59 (2024).

[45] F. Wei, Y. Zhao, A. Chen, X. Hou, P. Cong, New vauxiid sponges from the Chengjiang Biota and their evolutionary significance. J. Geol. Soc. London 178, doi.org/10.1144/jgs2020-162 (2021).

[46] S. Tarasov, Integration of anatomy ontologies and evo-devo using structured markov models suggests a new framework for modeling discrete phenotypic traits. Syst. Biol. 68, 698–716 (2019). 30668800 10.1093/sysbio/syz005 PMC6701457

[47] S. Tarasov, New phylogenetic Markov models for inapplicable morphological characters. Syst. Biol. 72, 681–693 (2023). 36788381 10.1093/sysbio/syad005 PMC10276624

[48] N. Shubin, C. Tabin, S. Carroll, Deep homology and the origins of evolutionary novelty. Nature 457, 818–823 (2009). 19212399 10.1038/nature07891

[49] S. Tarasov, The invariant nature of a morphological character and character state: Insights from gene regulatory networks. Syst. Biol. 69, 392–400 (2020). 31372653 10.1093/sysbio/syz050

[50] D. M. Emms, S. Kelly, OrthoFinder2: Fast and accurate phylogenomic orthology analysis from gene sequences. *BioRxiv* 10.1101/466201 (2018).

[51] B. Q. Minh, H. A. Schmidt, O. Chernomor, D. Schrempf, M. D. Woodhams, A. von Haeseler, R. Lanfear, IQ-TREE 2: New models and efficient methods for phylogenetic inference in the genomic era. Mol. Biol. Evol. 37, 1530–1534 (2020). 32011700 10.1093/molbev/msaa015 PMC7182206

[52] N. Lartillot, H. Philippe, A Bayesian mixture model for across-site heterogeneities in the amino-acid replacement process. Mol. Biol. Evol. 21, 1095–1109 (2004). 15014145 10.1093/molbev/msh112

[53] N. Lartillot, N. Rodrigue, D. Stubbs, J. Richer, PhyloBayes MPI: Phylogenetic reconstruction with infinite mixtures of profiles in a parallel environment. Syst. Biol. 62, 611–615 (2013). 23564032 10.1093/sysbio/syt022

[54] S. Mirarab, R. Reaz, M. S. Bayzid, T. Zimmermann, M. S. Swenson, T. Warnow, ASTRAL: Genome-scale coalescent-based species tree estimation. Bioinformatics 30, i541–i548 (2014). 25161245 10.1093/bioinformatics/btu462 PMC4147915

[55] Z. Yang, PAML 4: Phylogenetic analysis by maximum likelihood. Mol. Biol. Evol. 24, 1586–1591 (2007). 17483113 10.1093/molbev/msm088

[56] F. S. Dunn, A. G. Liu, D. V. Grazhdankin, P. Vixseboxse, J. Flannery-Sutherland, E. Green, S. Harris, P. R. Wilby, P. C. J. Donoghue, The developmental biology of Charnia and the eumetazoan affinity of the Ediacaran rangeomorphs. Sci. Adv. 7, eabe0291 (2021). 34301594 10.1126/sciadv.abe0291 PMC8302126

[57] C. Yang, Y. Li, D. Selby, B. Wan, C. Guan, C. Zhou, X.-H. Li, Implications for Ediacaran biological evolution from the ca. 602 Ma Lantian biota in China. Geology 50, 562–566 (2022).

[58] P. R. Wilby, J. N. Carney, M. P. Howe, A rich Ediacaran assemblage from eastern Avalonia: Evidence of early widespread diversity in the deep ocean. Geology 39, 655–658 (2011).

[59] S. R. Noble, D. J. Condon, J. N. Carney, P. R. Wilby, T. C. Pharaoh, T. D. Ford, U-Pb geochronology and global context of the Charnian Supergroup, UK: Constraints on the age of key Ediacaran fossil assemblages. Geol. Soc. Am. Bull. 127, 250–265 (2015).

[60] J. L. Thorne, H. Kishino, I. S. Painter, Estimating the rate of evolution of the rate of molecular evolution. Mol. Biol. Evol. 15, 1647–1657 (1998). 9866200 10.1093/oxfordjournals.molbev.a025892

[61] B. Rannala, Z. Yang, Inferring speciation times under an episodic molecular clock. Syst. Biol. 56, 453–466 (2007). 17558967 10.1080/10635150701420643

[62] C. D. Walcott, Middle Cambrian Spongiae. *Smithsonian Misc*. Collect. (1917).

[63] J. W. Salter, On some new fossils from the lingula-flags of Wales. Quart. J. Geol. Soc. London 20, 233–241 (1864).

[64] R. M. Finks, R. L. Kaesler, J. K. Rigby, “Paleozoic demospongea: Morphology Morphology and phylogeny,” in *Treatise on Invertebrate Paleontology, Pt. E, Porifera (revised)* (Geological Society of America, 2003), vol. 2, pp. 63–80.

[65] C. Luo, F. Zhao, H. Zeng, The first report of a vauxiid sponge from the Cambrian Chengjiang Biota. J. Paleo. 94, 28–33 (2020).

[66] X.-L. Yang, Y.-L. Zhao, L. E. Babcock, J. Peng, Siliceous spicules in a vauxiid sponge (Demospongia) from the Kaili Biota(Cambrian Stage 5), Guizhou, South China. Sci. Rep. 7, 42945 (2017). 28220860 10.1038/srep42945 PMC5318851

[67] K. A. Kolesnikov, J. P. Botting, A. Y. Ivantsov, A. Y. Zhuravlev, New early Cambrian sponges of the Siberian platform and the origins of spiculate crown-group demosponges. Pap. Palaeontol. 4, e1582 (2024).

[68] M. B. DeBiasse, A. Buckenmeyer, J. Macrander, L. S. Babonis, B. Bentlage, P. Cartwright, C. Prada, A. M. Reitzel, S. N. Stampar, A. G. Collins, A cnidarian phylogenomic tree fitted with hundreds of 18S leaves. Bull. Soc. Syst. Biol. 3, (2024).

[69] M. E. Alfaro, F. Santini, C. Brock, H. Alamillo, A. Dornburg, D. L. Rabosky, G. Carnevale, L. J. Harmon, Nine exceptional radiations plus high turnover explain species diversity in jawed vertebrates. Proc. Natl. Acad. Sci. U.S.A. 106, 13410–13414 (2009). 19633192 10.1073/pnas.0811087106 PMC2715324

[70] D. V. Lavrov, M. C. Diaz, M. Maldonado, C. C. Morrow, T. Perez, S. A. Pomponi, R. W. Thacker, Phylomitogenomics bolsters the high-level classification of Demospongiae (phylum Porifera). PLOS ONE 18, e0287281 (2023). 38048310 10.1371/journal.pone.0287281 PMC10695373

[71] S. Santini, Q. Schenkelaars, C. Jourda, M. Duchesne, H. Belahbib, C. Rocher, M. Selva, A. Riesgo, M. Vervoort, S. P. Leys, L. Kodjabachian, A. L. Bivic, C. Borchiellini, J.-M. Claverie, E. Renard, The compact genome of the sponge Oopsacas minuta (Hexactinellida) is lacking key metazoan core genes. BMC Biol. 21, 139 (2023). 37337252 10.1186/s12915-023-01619-w PMC10280926

[72] W. E. G. Müller, U. Schloßmacher, C. Eckert, A. Krasko, A. Boreiko, H. Ushijima, S. E. Wolf, W. Tremel, I. M. Müller, H. C. Schröder, Analysis of the axial filament in spicules of the demosponge Geodia cydonium: Different silicatein composition in microscleres (asters) and megascleres (oxeas and triaenes). Eur. J. Cell Biol. 86, 473–487 (2007). 17658193 10.1016/j.ejcb.2007.06.002

[73] K. Shimizu, J. Cha, G. D. Stucky, D. E. Morse, Silicatein α: Cathepsin L-like protein in sponge biosilica. Proc. Natl. Acad. Sci. U.S.A. 95, 6234–6238 (1998). 9600948 10.1073/pnas.95.11.6234 PMC27641

[74] K. Shimizu, T. Amano, M. R. Bari, J. C. Weaver, J. Arima, N. Mori, Glassin, a histidine-rich protein from the siliceous skeletal system of the marine sponge Euplectella, directs silica polycondensation. Proc. Natl. Acad. Sci. U.S.A. 112, 11449–11454 (2015). 26261346 10.1073/pnas.1506968112 PMC4577155

[75] M. Maldonado, A. Riesgo, Intra-epithelial spicules in a homosclerophorid sponge. Cell Tissue Res. 328, 639–650 (2007). 17340151 10.1007/s00441-007-0385-7

[76] N. de Voogd, B. Alvarez, N. Boury-Esnault, P. Cárdenas, M.-C. Díaz, M. Dohrmann, R. Downey, C. Goodwin, E. Hajdu, J. Hooper, M. Kelly, M. Klautau, S.-C. Lim, R. Manconi, C. Morrow, U. Pinheiro, A. Pisera, P. Ríos, K. Rützler, C. Schönberg, T. Turner, J. Vacelet, R. van Soest, J. Xavier, World Porifera Database. Accessed at https://www.marinespecies.org/porifera, VLIZ (2023); 10.14284/359.

[77] J. Vacelet, N. Boury-Esnault, Carnivorous sponges. Nature 373, 333–335 (1995).

[78] M.-J. Uriz, X. Turon, M. A. Becerro, G. Agell, Siliceous spicules and skeleton frameworks in sponges: Origin, diversity, ultrastructural patterns, and biological functions. Micros. Res. Tech. 62, 279–299 (2003). 10.1002/jemt.10395 14534903

[79] M. Łukowiak, R. Van Soest, M. Klautau, T. Pérez, A. Pisera, K. Tabachnick, The terminology of sponge spicules. J. Morphol. 283, 1517–1545 (2022). 36208470 10.1002/jmor.21520

[80] C. Morrow, P. Cárdenas, Proposal for a revised classification of the Demospongiae (Porifera). Front. Zool. 12, 7 (2015). 25901176 10.1186/s12983-015-0099-8 PMC4404696

[81] M. dos Reis, Y. Thawornwattana, K. Angelis, M. J. Telford, P. C. J. Donoghue, Z. Yang, Uncertainty in the timing of origin of animals and the limits of precision in molecular timescales. Curr. Biol. 25, 2939–2950 (2015). 26603774 10.1016/j.cub.2015.09.066 PMC4651906

[82] J. B. Antcliffe, Questioning the evidence of organic compounds called sponge biomarkers. Palaeontology 56, 917–925 (2013).

[83] D. A. Gold, S. S. O’Reilly, G. Luo, D. E. G. Briggs, R. E. Summons, Prospects for sterane preservation in sponge fossils from museum collections and the utility of sponge biomarkers for molecular clocks. Bull. Peabody Mus. Nat. Hist. 57, 181–189 (2016).

[84] B. J. Nettersheim, J. J. Brocks, A. Schwelm, J. M. Hope, F. Not, M. Lomas, C. Schmidt, R. Schiebel, E. C. M. Nowack, P. De Deckker, J. Pawlowski, S. S. Bowser, I. Bobrovskiy, K. Zonneveld, M. Kucera, M. Stuhr, C. Hallmann, Putative sponge biomarkers in unicellular Rhizaria question an early rise of animals. Nat. Ecol. Evol. 3, 577–581 (2019). 30833757 10.1038/s41559-019-0806-5

[85] L. Shawar, G. D. Love, B. T. Uveges, J. A. Zumberge, P. Cárdenas, J.-L. Giner, R. E. Summons, Chemical characterization of C_31_ sterols from sponges and Neoproterozoic fossil sterane counterparts. Proc. Natl. Acad. Sci. U.S.A. 122, e2503009122 (2025). 41021825 10.1073/pnas.2503009122 PMC12541326

[86] Z. Wang, X. Xie, Z. Wen, Formation conditions of Ediacaran–Cambrian cherts in South China: Implications for marine redox conditions and paleoecology. Precambrian Res. 383, 106867 (2022).

[87] A. Muscente, F. M. Michel, J. G. Dale, S. Xiao, Assessing the veracity of Precambrian ‘sponge’fossils using in situ nanoscale analytical techniques. Precambrian Res. 263, 142–156 (2015).

[88] M. J. Donoghue, J. A. Doyle, J. Gauthier, A. G. Kluge, T. Rowe, The importance of fossils in phylogeny reconstruction. Annu. Rev. Ecol. Syst. 20, 431–460 (1989).

[89] C. Patterson, Significance of fossils in determining evolutionary relationships. Annu. Rev. Ecol. Syst. 12, 195–223 (1981).

[90] K. J. Peterson, R. E. Summons, P. C. Donoghue, Molecular palaeobiology. Palaeontology 50, 775–809 (2007).

[91] L.-T. Nguyen, H. A. Schmidt, A. von Haeseler, B. Q. Minh, IQ-TREE: A fast and effective stochastic algorithm for estimating maximum-likelihood phylogenies. Mol. Biol. Evol. 32, 268–274 (2015). 25371430 10.1093/molbev/msu300 PMC4271533

[92] J. P. Botting, P. Cárdenas, J. S. Peel, A crown-group demosponge from the early Cambrian Sirius Passet Biota, North Greenland. Palaeontology 58, 35–43 (2015).

[93] P. Cárdenas, Surface microornamentation of demosponge sterraster spicules, phylogenetic and paleontological implications. Front. Mar. Sci. 7, doi.org/10.3389/fmars.2020.613610 (2020).

[94] F. Ronquist, M. Teslenko, P. van der Mark, D. L. Ayres, A. Darling, S. Höhna, B. Larget, L. Liu, M. A. Suchard, J. P. Huelsenbeck, MRBAYES 3.2: Efficient Bayesian phylogenetic inference and model selection across a large model space. Syst. Biol. 61, 539–542 (2012). 22357727 10.1093/sysbio/sys029 PMC3329765

[95] D. S. Porto, J. Uyeda, I. Mikó, S. Tarasov, Ontophylo: Reconstructing the evolutionary dynamics of phenomes using new ontology-informed phylogenetic methods. Methods Ecol. Evol. 15, 290–300 (2024).

[96] D. L. Rabosky, Automatic detection of key innovations, rate shifts, and diversity-dependence on phylogenetic trees. PLOS ONE 9, e89543 (2014). 24586858 10.1371/journal.pone.0089543 PMC3935878

[97] S. Andrews, FastQC: A quality control tool for high throughput sequence data, (2010). Retrieved from: https://www.bioinformatics.babraham.ac.uk/projects/fastqc/.

[98] A. M. Bolger, M. Lohse, B. Usadel, Trimmomatic: A flexible trimmer for Illumina sequence data. Bioinformatics 30, 2114–2120 (2014). 24695404 10.1093/bioinformatics/btu170 PMC4103590

[99] M. G. Grabherr, B. J. Haas, M. Yassour, J. Z. Levin, D. A. Thompson, I. Amit, X. Adiconis, L. Fan, R. Raychowdhury, Q. Zeng, Z. Chen, E. Mauceli, N. Hacohen, A. Gnirke, N. Rhind, F. di Palma, B. W. Birren, C. Nusbaum, K. Lindblad-Toh, N. Friedman, A. Regev, Full-length transcriptome assembly from RNA-Seq data without a reference genome. Nat. Biotechnol. 29, 644–652 (2011). 21572440 10.1038/nbt.1883 PMC3571712

[100] L. Fu, B. Niu, Z. Zhu, S. Wu, W. Li, CD-HIT: Accelerated for clustering the next-generation sequencing data. Bioinformatics 28, 3150–3152 (2012). 23060610 10.1093/bioinformatics/bts565 PMC3516142

[101] K. M. Kocot, M. R. Citarella, L. L. Moroz, K. M. Halanych, PhyloTreePruner: A phylogenetic tree-based approach for selection of orthologous sequences for phylogenomics. Evol. Bioinform. Online 9, 429–435 (2013). 24250218 10.4137/EBO.S12813 PMC3825643

[102] S. Whelan, I. Irisarri, F. Burki, PREQUAL: Detecting non-homologous characters in sets of unaligned homologous sequences. Bioinformatics 34, 3929–3930 (2018). 29868763 10.1093/bioinformatics/bty448

[103] R. C. Edgar, MUSCLE: Multiple sequence alignment with high accuracy and high throughput. Nucleic Acids Res. 32, 1792–1797 (2004). 15034147 10.1093/nar/gkh340 PMC390337

[104] S. Capella-Gutiérrez, J. M. Silla-Martínez, T. Gabaldón, trimAl: A tool for automated alignment trimming in large-scale phylogenetic analyses. Bioinformatics 25, 1972–1973 (2009). 19505945 10.1093/bioinformatics/btp348 PMC2712344

[105] J. A. Ballesteros, G. Hormiga, A new orthology assessment method for phylogenomic data: Unrooted phylogenetic orthology. Mol. Biol. Evol. 33, 2117–2134 (2016). 27189539 10.1093/molbev/msw069

[106] M. dos Reis, Z. Yang, Approximate likelihood calculation on a phylogeny for Bayesian estimation of divergence times. Mol. Biol. Evol. 28, 2161–2172 (2011). 21310946 10.1093/molbev/msr045

[107] H. C. Betts, M. N. Puttick, J. W. Clark, T. A. Williams, P. C. J. Donoghue, D. Pisani, Integrated genomic and fossil evidence illuminates life’s early evolution and eukaryote origin. Nat. Ecol. Evol. 2, 1556–1562 (2018). 30127539 10.1038/s41559-018-0644-x PMC6152910

[108] R. J. Howard, M. Giacomelli, J. Lozano-Fernandez, G. D. Edgecombe, J. F. Fleming, R. M. Kristensen, X. Ma, J. Olesen, M. V. Sørensen, P. F. Thomsen, M. A. Wills, P. C. J. Donoghue, D. Pisani, The Ediacaran origin of Ecdysozoa: Integrating fossil and phylogenomic data. J. Geol. Soc. London 179, doi.org/10.1144/jgs2021-107 (2022).

[109] K. Angelis, S. Álvarez-Carretero, M. Dos Reis, Z. Yang, An evaluation of different partitioning strategies for bayesian estimation of species divergence times. Syst. Biol. 67, 61–77 (2018). 29029343 10.1093/sysbio/syx061 PMC5790132

[110] L. J. Revell, phytools: An R package for phylogenetic comparative biology (and other things). Methods Ecol. Evol. 3, 217–223 (2012).

[111] M. Pagel, Detecting correlated evolution on phylogenies: A general method for the comparative analysis of discrete characters. Proc. Royal Soc. London B 255, 37–45 (1997).

[112] S. Álvarez-Carretero, A. U. Tamuri, M. Battini, F. F. Nascimento, E. Carlisle, R. J. Asher, Z. Yang, P. C. J. Donoghue, M. dos Reis, A species-level timeline of mammal evolution integrating phylogenomic data. Nature 602, 263–267 (2022). 34937052 10.1038/s41586-021-04341-1

[113] D. L. Rabosky, M. Grundler, C. Anderson, P. Title, J. J. Shi, J. W. Brown, H. Huang, J. G. Larson, BAMMtools: An R package for the analysis of evolutionary dynamics on phylogenetic trees. Methods Ecol. Evol 5, 701–707 (2014).

[114] J. J. Matthews, A. G. Liu, C. Yang, D. M. Ilroy, B. Levell, D. J. Condon, A chronostratigraphic framework for the rise of the Ediacaran macrobiota: New constraints from Mistaken Point Ecological Reserve, Newfoundland. Geol. Soc. Am. Bull. 133, 612–624 (2021).

[115] F. S. Dunn, C. G. Kenchington, L. A. Parry, J. W. Clark, R. S. Kendall, P. R. Wilby, A crown-group cnidarian from the Ediacaran of Charnwood Forest, UK. Nat. Ecol. Evol. 6, 1095–1104 (2022). 35879540 10.1038/s41559-022-01807-x PMC9349040

[116] Z. Yin, W. Sun, P. Liu, M. Zhu, P. C. J. Donoghue, Developmental biology of Helicoforamina reveals holozoan affinity, cryptic diversity, and adaptation to heterogeneous environments in the early Ediacaran Weng’an biota (Doushantuo Formation, South China). Sci. Adv. 6, eabb0083 (2020). 32582859 10.1126/sciadv.abb0083 PMC7292632

[117] Z. Yin, K. Vargas, J. Cunningham, S. Bengtson, M. Zhu, F. Marone, P. Donoghue, The early Ediacaran Caveasphaera foreshadows the evolutionary origin of animal-like embryology. Curr. Biol. 29, 4307–4314.e2 (2019). 31786065 10.1016/j.cub.2019.10.057

[118] C. Yang, A. D. Rooney, D. J. Condon, X.-H. Li, D. V. Grazhdankin, F. T. Bowyer, C. Hu, F. A. Macdonald, M. Zhu, The tempo of Ediacaran evolution. Sci. Adv. 7, eabi9643 (2021). 34731004 10.1126/sciadv.abi9643 PMC8565906

[119] M. J. Benton, P. C. J. Donoghue, R. J. Asher, M. Friedman, T. J. Near, J. Vinther, Constraints on the timescale of animal evolutionary history. Palaeont. Electr. 18, 1–106 (2015).

[120] X.-P. Dong, J. A. Cunningham, S. Bengtson, C.-W. Thomas, J. Liu, M. Stampanoni, P. C. J. Donoghue, Embryos, polyps and medusae of the Early Cambrian scyphozoan Olivooides. Proc. Biol. Sci. 280, 20130071 (2013). 23446532 10.1098/rspb.2013.0071 PMC3619488

[121] F. T. Bowyer, A. Y. Zhuravlev, R. Wood, G. A. Shields, Y. Zhou, A. Curtis, S. W. Poulton, D. J. Condon, C. Yang, M. Zhu, Calibrating the temporal and spatial dynamics of the Ediacaran-Cambrian radiation of animals. Earth Sci. Rev. 225, 103913 (2022).

[122] M. Steiner, G. Li, Y. Qian, M. Zhu, B.-D. Erdtmann, Neoproterozoic to early Cambrian small shelly fossil assemblages and a revised biostratigraphic correlation of the Yangtze Platform (China). Palaeogeogr. Palaeoclimatol. Palaeoecol. 254, 67–99 (2007).

[123] B. Runnegar, Muscle scars, shell form and torsion in Cambrian and Ordovician univalved molluscs. Lethaia 14, 311–322 (1981).

[124] M. Steiner, G. Li, Y. Qian, M. Zhu, Lower Cambrian Small Shelly Fossils of northern Sichuan and southern Shaanxi (China), and their biostratigraphic importance (Small Shelly Fossils du Cambrien inférieur du nord du Sichuan et du sud du Shaanxi (Chine) et leur importance biostratigraphique). Geobios 37, 259–275 (2004).

[125] H. Mostler, Mikroskleren von demospongien (Porifera) aus dem basalen Jura der nördlichen Kalkalpen. Geo.-Paläontol. Mitt. Innsbruck 17, 119–142 (1990).

[126] M. Łukowiak, Fossil and modern sponge fauna of southern Australia and adjacent regions compared: Interpretation, evolutionary and biogeographic significance of the late Eocene ‘soft’ sponges. Smithson. Contrib. Zool. 85, 13–35 (2016).

[127] N. P. James, Y. Bone, Eocene cool-water carbonate and biosiliceous sedimentation dynamics, St Vincent Basin, South Australia. Sedimentology 47, 761–786 (2000).

[128] K. A. Ritterbush, S. Rosas, F. A. Corsetti, D. J. Bottjer, A. J. West, Andean sponges reveal long-term benthic ecosystem shifts following the end-Triassic mass extinction. Palaeogeogr. Palaeoclimatol. Palaeoecol. 420, 193–209 (2015).

[129] D. Ungureanu, F. Ahmad, S. Farouk, A Callovian (Middle Jurassic) poriferan fauna from northwestern Jordan: Taxonomy, palaeoecology and palaeobiogeography. Hist. Biol. 30, 577–592 (2018).

[130] T. Schindler, M. Wuttke, M. Poschmann, Oldest record of freshwater sponges (Porifera: Spongillina)—Spiculite finds in the Permo-Carboniferous of Europe. Paläontol. Z. 82, 373–384 (2008).

[131] S. Fortunato, M. Adamski, B. Bergum, C. Guder, S. Jordal, S. Leininger, C. Zwafink, H. T. Rapp, M. Adamska, Genome-wide analysis of the sox family in the calcareous sponge Sycon ciliatum: Multiple genes with unique expression patterns. Evodevo 3, 14 (2012). 22824100 10.1186/2041-9139-3-14 PMC3495037

