# Supplementary material for: Independent origins of spicules reconcile paleontological and molecular evidence of sponge evolutionary history
Source: Sci Adv. 2026 Jan 7;12(2):eadx1754. doi: 10.1126/sciadv.adx1754 (PMC12778063; doi:10.1126/sciadv.adx1754)
Supplement: Supplementary file 1 — Supplementary Text Figs. S1 to S18 Tables S1 to S13 Legend for data S1 References [file sciadv.adx1754_sm.pdf]

Science Advances
AAAS

Supplementary Materials for

Independent origins of spicules reconcile paleontological and molecular evidence of sponge evolutionary history

Maria Eleonora Rossi *et al.*

Corresponding author: Philip C. J. Donoghue, phil.donoghue@bristol.ac.uk;
Ana Riesgo, anariesgogil@mncn.csic.es; Davide Pisani, davide.pisani@bristol.ac.uk

Sci. Adv. **12**, eadx1754 (2026)
DOI: 10.1126/sciadv.adx1754

The PDF file includes:

Supplementary Text
Figs. S1 to S18
Tables S1 to S13
Legend for data S1
References

Other Supplementary Material for this manuscript includes the following:

Data S1

Supplementary Text

Supplementary Methods

Trascriptomes assembly and annotation

For all transcriptomes assembled, read quality assessment was performed with FastQC (*97*) with trimming performed with Trimmomatic (*98*) with the following settings: ILLUMINACLIP:./Adapters.fa:2:30:10 LEADING:3 TRAILING:3 SLIDINGWINDOW:4:28 MINLEN:36, with the Adapters.fa file adjusted to include the adapter sequences specific to each read pair. Assembly of the clean paired reads, obtained from Trimmomatic, was performed using Trinity (*99*) with the following settings: --normalize_reads –CPU 8 –inchwormcpu 8. Prior to orthology inference, nucleotide sequences were translated using TransDecoder (https://hpc.nih.gov/apps/TransDecoder.html) for a total of 70 species and isoforms were collapsed using CD-HIT (*100*). The final total dataset was composed of 70 proteomes (Table S10).

Orthofinder-based pipeline for the identification of orthologous gene families

The Orthofinder v. 2.7.1 (*50*) analysis identified an initial set of 335,892 orthogroups. Putative orthologous sequences in each orthogroups were identified using PhyloTreePruner (*101*). Only sets of orthologous sequences that retained at the least 90% of the species in our dataset (a total of 1,468 orthogroups) were retained for further analyses. This was done to minimise missing data. For each one of the 1,468 orthogroups retained, hypervariable regions were removed using PREQUAL (*102*). After that, orthogroups were individually aligned using MUSCLE (*103*), and trimmed with TrimAl (*104*) (*-auto* option). Individual sequences that after TrimAl were shorter than 25 AA were removed using Al2Phylo (*105*). If an orthogroup had less than 50% of the total number of sequences in our dataset left, after the Al2Phylo step, the orthogroup was removed from the analysis. After this step our dataset was composed of 1,286 orthogroups. Single-gene trees were built for each one of the 1,286 genes retained using IQTree v 1.6.12 (*92*), where a model test was run for each gene tree with the parameters -m MFP -mset LG+F+G, WAG+F+G, JTT+F+G, GTR20 -madd LG+C20+F+G, LG+C10+F+G, LG+C30+F+G, LG+C40+F+G, LG+C50+F+G, LG+C60+F+G, C10, C20, C30, C40, C50, C60, LG4M, LG4X to allow selecting across compositionally site homogeneous and site heterogeneous models. The 1,286 gene trees were screened using a script developed by Mattia Giacomelli and Paschalis Natsidis https://github.com/pnatsi/ParaFilter to remove putative long branched taxa. Finally, we used monophylyl.pl from https://github.com/MaxTelford/Xenacoelomorpha2019 to identify and remove orthogroups corresponding to gene trees that failed to resolve Porifera as monophyletic group. At the end of this final step we were left with a final dataset of 133 orthogroups that were used in our phylogenetic analyses. See Material and Methods in main text for the details of our concatenated phylogenomic analyses, that were performed using Bayesian and ML methods. We also analysed our final dataset using Astral on the 133 gene trees corresponding to the 133 genes

in our datasets. To infer these gene trees, we used IQTree (same parameters used for the inference of the gene trees reported above).

Divergence time analyses

We run timetree inference analyses with the approximate likelihood approach (106). We ran all analyses under both an IR and AR relaxed-clock models implemented in MCMCtree (PAML package v4.9i (*55*)). Six independent chains for every analysis (SDN+AR, SDN+IR, SPN+AR, SPN+IR) were run with 4,020,000 iterations each. As per our MCMC settings, we discarded the first 100,000 collected samples as part of the burn-in phase and, every 100 iterations, we saved the values sampled for each parameter until reaching a total of 20,000 samples for each chain. The MCMC settings for the evolutionary model can be found in the SM. We run six MCMC chains for both our prior and posterior estimations and used an in-house pipeline (see https://doi.org/10.5281/zenodo.11488993) to (i) filter these chains and (ii) generate convergence plots and summary statistics. The plots and summary statistics only used the chains passing our quality control checks (see Zenodo repository for methodological details).

To estimate species divergence times, we dated our inferred Bayesian phylogeny (Fig. S1) using MCMCtree, which is part of the PAML package (*55*), with the approximate likelihood approach (*106*). CODEML, also part of PAML, was first used to calculate the first and second derivatives of the likelihood function (i.e., the gradient and the Hessian) and the branch lengths under maximum likelihood, which are then used by MCMCtree to approximate the likelihood function using the Taylor expansion (see PAML documentation).

To improve precision in divergence time estimation, it is best to partition a superalignment into various alignment blocks, so that each block (or partition) contains subsets of genes that share the same evolutionary process (and thus evolve at the same, or similar, evolutionary rate) (*81*, *107*, *108*). However, the number of chosen partitions cannot be arbitrarily large; including more than 10 partitions can artificially inflate the precision with which divergence times are estimated(*109*). Consequently, we ran IQtree2 (*92*) to find the best-fitting model for each gene alignment that was part of the superalignment, but considered only those models available in PAML. A total of 5 different models were selected as the best-fitting evolutionary models, which allowed us to group our gene alignments into five different alignment blocks. For each alignment block, we selected the relevant rate matrix according to the best-fitting evolutionary model so that CODEML could estimate the branch lengths, the gradient, and the Hessian later required by MCMCtree (see PAML documentation). The first (68 taxa and 25,620 AA) and second (68 taxa and 25,620 AA) partitions used independently parameterised JTT+G4 models. These gene alignments were separately classified as IQtree identified the best-fitting models as having very different alpha parameters for the Gamma distribution that accounts for rate heterogeneity. Please note that IQtree inferred the best-fitting model of one of these alignment blocks as JTT+G4+I, which is not implemented in

PAML, hence we had to simplify this model to JTT+G4 instead when using CODEML. The model for the third partition was DCMut+G4 (66 taxa and 7,388 AA). The fourth (69 taxa and 9,947 AA) and fifth (70 taxa and 49,102 AA) partitions used independently parameterised LG+G4 models; same scenario that occurred with the JTT models as aforementioned. The main output of CODEML is a file called "rst2", which contains the vectors and the matrix. The content of the five "rst2" files (i.e., one per partition) were then concatenated in the so-called "in.BV" file (see PAML documentation).

To estimate divergence times, MCMCtree was then run with the 5-partition in.BV file, the 5-partition AA alignment, and the fixed tree topology of fig. S1 with 12 calibrations (see below List of Calibrations and their Justifications in SM). The prior on divergence times consists of a birth-death process with species sampling (*8*) that we defined with $\lambda = \mu = 1$ (birth and death rates, respectively) and a sampling fraction of $\rho = 0.1$. The resulting kernel density is an approximate uniform distribution that is used to determine the distribution of the node ages for uncalibrated nodes. We used a gamma-Dirichlet rate prior on the mean i-*th* partition rate, μi ~ Γ (2,20) so that the mean evolutionary rate is 0.1 substitutions per site per 100 Myr, the time unit we defined. We used a more diffuse gamma-Dirichlet prior on the variance of the rate for each locus *i*, σi2 ~Γ (1, 10), to account for the violation of the clock that is expected in deep phylogenies. For each combination of clock models and calibration strategy (SDN+AR, SDN+IR, SPN+AR, and SPN+IR – see main for abbreviations), we ran 6 independent chains. We discarded the first 100,000 iterations as part of the burn-in phase and collected a total of 20,000 samples every 100 iterations. In total, all chains ran for 2,100,000 iterations.

MCMC diagnostics

We assessed the quality of the six chains we ran for each analysis. We calculated the mean lower- and upper-quantiles (i.e., 2.5%- and 97.5%-quantiles) of each time distribution (i.e., mean lower- and upper-quantiles for each node in the phylogeny) for each node in Fog. S3, and each of the six chains we ran per analysis. In an iterative manner, we used our in-house R function check_quantiles to compute, for each node, the difference between the quantiles computed for each chain. This is an iterative process where we start denominating Chain 1 as the "main" chain, and calculate differences between the main chain (Chain 1 in the first instance) and the other 5 chains. After that, the procedure is repeated while considering each one of the other six chains as the main chain. For each node, the resulting differences are compared to a predefined threshold value, α, to establish whether the chain being compared to the "main" chain should be flagged as potentially "problematic". If the difference between quantiles is either too large or too low, there may have been convergence issues, and the compared chain is flagged as potentially problematic. Chains labelled as "problematic" are not used for summary statistics nor to compute the mean divergence times. Note that whether a chain is deemed problematic or not may depend

on what chain is used as "main". If the chain used as main is problematic, all (or almost all) of the other chains would be flagged as problematic. Accordingly, to avoid using a potentially problematic chain as main, once all 6 chains are tested, the one that minimises the identification of problematic chains when used as the "main" chain is selected as the "reference" chain. Chains identified as problematic when using the reference chain were not used in our estimation of divergence times. Note that while for shallow phylogenies we can use a stringent threshold value (e.g., $\alpha = 0.05$ or $\alpha = 0.1$), deeper phylogenies require more relaxed values as the uncertainty in time estimates increases (i.e., larger differences between quantiles are expected). For our phylogeny, we used a threshold value of $\alpha = 0.25$, and did not keep chains for which, for at least one node, quantile differences were either larger or lower than $\alpha = 0.25$ (see supplementary tables S3-4 for the results with SDN+AR, SDN+IR, SPN+AR, and SPN+IR).

We wrote a wrapper function around the R function rstan::monitor v.2.21.7 to (i) calculate the effective sample size (ESS) for bulk and tail quantiles as well as (ii) the potential scale reduction factor on rank normalised split chains (i.e., the Rhat value) for all divergence times estimated with the samples collected by the chains that passed our filters. Chain convergence is theoretically assumed if ESS values are over 100 and Rhat values are smaller than or equal to 1.05. After summarising our parameter estimates with the filtered chains, the bulk- and tail-ESS values were over 100 and the Rhat values were smaller than 1.05 for all our model parameters of interest (see supplementary tables S1-S2). To show the quality of our statistical QCs, figure S6 is used to compare the convergence plots generated with all the chains and then with only those chains we kept after applying our filters. To build the plots, if 4 chains had passed our filters, the average of all the divergence times was calculated with the first 2 chains and with the last 2 chains. The estimated mean divergence times were then plotted against each other. If the resulting convergence plot shows an almost straight line (i.e., x =~ y), the chains are assumed to have converged. In the plots with unfiltered chains, some points deviate from the "x =~ y" line, suggesting potential convergence problems for some chains. These issues disappear when only chains retained after filtering are used. All the data, scripts, and step-by-step guidelines required to reproduce these analyses can be found in our zenodo repository.

Ancestral state estimation analyses

Assembling a phylogenetic tree with fossils and extant taxa

The trees in fig. S12 were time-scaled using the timeplaeophy() function in the paleotree R package (*110*), specifying the 'equal' method. Fossil tip ages were taken from (*42*) and internal node ages were fixed based on our molecular clock analyses. We generated 12 alternate timescaled trees, representing all possible combinations of three variables: (I) tree topology (a - a tree without fossils; b - fossils unconstrained; c - fossils constrained to follow *(42)*); (II) molecular timescale (a - autocorrelated rates, b - independent rates); (III) calibration strategy (a - SDN; b - SPN). The

input files used to run these analyses are available in Figshare (https://doi.org/10.6084/m9.figshare.28574570).

Estimation of ancestral state

We performed marginal likelihood ancestral state estimation in R using the fitMk() and ancr() functions from the phytools package (*110*). We amalgamated the rate matrices of independent characters using structured Markov models (*46*, *111*), while dependent characters were amalgamated using embedded dependencies (*47*). We tested four model combinations:

We tested four model combinations:

- Model A: characters (ii) + (iii)
- Model B: characters (i) + (ii) + (iii)
- Model C: characters (ii) + (iii) + (iv)
- Model D: characters (i) + (ii) + (iii) + (iv)

To account for rate heterogeneity, we applied both Equal Rates (ER) and All Rates Different (ARD) models. Each model combination was run under all possible permutations of ER and ARD across the component characters. For instance, in Model A (characters ii and iii), we ran:

- Char(ii)_ER + Char(iii)_ER
- Char(ii)_ER + Char(iii)_ARD
- Char(ii)_ARD + Char(iii)_ER
- Char(ii)_ARD + Char(iii)_ARD

The number of model combinations for each set of characters thus equals 2^n, where n is the number of characters amalgamated. Each amalgamated model was applied to 12 time-scaled phylogenies (see previous section). For each model-tree combination, we calculated the Bayesian Information Criterion (BIC) and corresponding model weight. We then plotted the model-averaged ASE results

for each tree. In total, we conducted 432 ancestral state estimations. All analysis scripts are available at https://doi.org/10.6084/m9.figshare.28574570.

Diversification Rates through Time and Across Clades

Inference of the dated Silicean megaphylogeny

We generated a megaphylogeny of sponges using 804 *COI* sequence sequences that were visualised using Seaview and aligned with MUSCLE v. 3.8.31(*103*). The final alignment was 609 bp. For these sequences we inferred the best-scoring maximum-likelihood (ML) tree with IQTree2 v2.1.3 (*92*). However, we followed (*68*) and constrained the analyses using our phylogenomic tree (Fig. 1; fig. S1) as a backbone constraint as COI sequences can be expected to resolve tip-ward nodes of our phylogeny, but not nodes that are deeper in our tree. Model testing was performed using Model Finder plus (MFP option in IQTree but considering only across-site compositionally homogeneous models) because of the size of the dataset which limited our ability to use complex models. We dated the megaphylogeny following the procedure detailed in (*112*). We fitted skew-t (ST) distributions to the posterior time densities inferred using PAML v4.9i (*54*) (see SM). We selected 13 ST distributions that matched the nodes that had been calibrated prior to timetree inference so that we could use them as prior distributions in the subsequent analyses (table S12) on the ML topology inferred using the 807 COI sequences. We ran 64 independent chains with 4,020,000 iterations each. We discarded the first 20,000 iterations as part of the burn-in phase and subsequently collected samples every 20 iterations. We saved the values sampled for each parameter until reaching a total of 200,000 samples for each chain. Convergence was calculated as explained in the "Timetree inference" section above (see also Table S7).

Estimation of diversification rate shifts on the dated megaphylogenies

We accounted for incomplete taxon sampling by defining a sampling fraction of 5%, which corresponds to the number of species included in the COI dataset compared to the total number of Silicea species accepted. For each order we also calculated the sampling frequencies (species sampled/species accepted); data obtained from the World Porifera Database (*77*). These sampling frequencies are reported in table S13. In BAMM v.2.5.0 (*69*) ten million generations of reversible

jump Markov Chain Monte Carlo sampling were run, drawing samples from the posterior every 10,000 generations. For the prior probability of rate shift, we tested values ranging from 0.1 to 50 and chose the value leading to the highest ESS values for LogLikelihood and NumberOfShifts (table S8). We processed the output data with BAMMtools (*113*) and, after removing the samples collected during the burn-in process (10% of the samples), we obtained the summary statistics, and plotted the diversification rate over time. To overcome the possible bias introduced by incomplete sampling in diversification analyses, we confirmed our results using the taxonomic method implemented in MEDUSA (*70*) where clades are collapsed to terminal lineages of equal ranks. Only diversification events inferred by both methods were deemed reliable.

List of Calibrations and their justifications.

Fossil Provenance: This study did not collect or analyze fossil specimens. All information was collated from the study of the literature.

In the following list calibrated nodes are indicated numerically with reference to the labels in fig. S18.

Node 71 (fig. S18): Crown Metazoa | 574-609 Ma

Fossil taxon and specimen: *Charnia masoni* (LEIUG 2328 from Bed B of North Quarry, Charnwood Forest, UK).

Phylogenetic justification (*56*) demonstrated that *Charnia masoni* is a stem-eumetazoan based on analysis of its morphogenesis and comparative taphonomy.

Minimum age justification: The earliest occurrence of *Charnia masoni* is found in the Drook Formation of Mistaken Point, Newfoundland, which has been dated to 574.17 Ma ± 0.66 Myr, providing for a minimum constraint of 573.51 Ma (*114*). For the analysis we set the minimum is 574 to avoid truncation issues as explained in the Supplementary Method section above and in Material and Methods.

Soft maximum age justification: We establish the minimum constraint on the Lantian biota of South China which contains a diversity of macrofossils but nothing that can definitively be classified as a metazoan. A Re-Os age for the Lantian of 602 Ma ± 7 Ma was derived by (*56*), resulting in a maximum constraint of 609 Ma. This age allows for the possibility of *Eoandromeda* being a crown metazoan.

Node 135 (fig. S18): Crown Eumetazoa | 561.1-590.8 Ma

Fossil taxon and specimen: *Auroralumina attenboroughii* (GSM 106119) from Bed B, Bradgate Formation, Charnian Supergroup of North Quarry, Charnwood Forest, UK (*115*).

Phylogenetic justification: a crown-cnidarian affinity for *Auroralumina attenboroughii* has been established based on a formal character analysis (*114*).

Minimum age justification: The age of Bed B within the Bradgate Formation has been constrained to 563 Ma ± 1.9 Myr (*58*). Later a U-Pb date of 556.6 ± 6.4 was derived by (*58*), but the associated uncertainty entirely encompasses the original, more precise date. Hence, we follow the earlier date from (*58*).

Soft maximum age justification: We establish the minimum constraint on the Weng'an biota of South China which contains a diversity of microfossils, many of which have been interpreted as metazoans in the past (*21*), but which have subsequently been reinterpreted as stem-metazoans, at best (*116*, *117*). A maximum age of 587.2 Ma ±3.6 Myr for the Weng'an Biota has been

established based on the Re-Os system (*118*). This provides for a maximum constraint of 590.8 Ma.

Node 138 (fig. S18): Crown Cnidaria | 531.80 Ma.
Fossil Taxon specimen: *Olivooides multisulcatus* (Geological Museum of Peking University: GMPKU3083-GMPKU3090), Dengying Formation, Fortunian Stage, constituting a range of embryonic and post-embryonic developmental stages. This calibration largely follows (*119*) from which most of the description is derived.
Phylogenetic justification: *Olivooides* is known from embryonic and post-embryonic stages of development, including a polyp theca, characteristic of scyphozoans, and a medusa stage (*120*).
Minimum age justification: *Olivooides multisulcatus* co-occurs with *Anabarites trisulcatus*, which is indicative of the middle of the Fortunian Stage of the Terreneuvian Series, the first of the Cambrian. We derive a numeric age of 531.80 Ma following the age model of (*121*).

Node 139 (fig. S18): Crown Bilateria | 532 Ma.
Fossil taxon and specimen: *Aldanella janjiahensis* (YXII102-02), often synonymized with *Aldanella attleborensis,* from the Lower Cambrian Dahai Member of Zhujiaqing Formation, Xiaotan, Yongshan County, Yunnan (*122*).
Phylogenetic justification: *Aldanella* is a dextrally coiled mollusk assigned to the Pelagiellida. The distinct asymmetries and the preservation of muscle scars (*123*) suggest that it is a partially coiled stem-group gastropod.
Minimum age justification: The fossil occurrence falls fully within the *Anabarites trisulcatus–Protohetzina anabarica* Assemblage Biozone (*124*), falling within the span of 537 – 532 Ma.

Node 72 and Node 75 (fig. S18): Crown Demospongiae | 515 Ma.
Fossil Taxon Specimen: Demosponge indent (MGUH 30886 from GGU sample 340103.3684), from the Sirius Passet Lagerstätte, Buen Formation, Peary Land, North Greenland (*93*).
Phylogenetic justification: The combination of spicule forms (monaxons with small sigma, toxa and unique spiral morphologies) places the sponge as the first crown-group demosponge, thus representing the stem lineage of Heteroscleromorpha.
Minimum age justification: The fossil occurrence falls within the Cambrian Series 2, Stage 3 and therefore presents a reliable calibration point for the minimum root age of the group Haplosclerida + Heteroscleromorpha which is ~ 515 Ma (*124*).

Node 88 (fig. S18): Poecilosclerida | 199 Ma

Fossil Taxon Specimen: Poecilosclerida, Lower Liassic Kirchsteinkalk (Allgäu Formation as basin facies links to Liassic Kirchsein Limestones) of the Northern Calcareous Alps in Germany).
Phylogenetic justification: Based on the morphological characterization of a dense occurrence of C-shaped "sigma microscleres" (type forceps) that are distinct to other known sigmas within demosponges, thus most similar to recent poecilosclerid sigmas, as well as the presence of various chelae spicules (*125*). Chelae are a synapomorphy of the Poecilosclerida.
Minimum age justification: Isochelae, are for the first time undoubtfully reported from the Early Jurassic Hettangian/Sinemurian boundary 199.3 Ma.

Node 83 (fig. S18): Tethya | 37.8 Ma
Fossil Taxon Specimen: *Tethyastra* sp. ZPAL Pf.26, St. Vincent Basin (Blanche Point section)
Phylogenetic justification: numerous oxyasters (Figs. 13F–H) (*30*) resembling those of recent *Tethyastra oxyaster* Burton, 1934. The great morphological resemblance and the occurrence of recent *T. oxyaster* from all over Australia (*126*) confirm the assignment to the family Tethyidae and the Order Tethyida.
Minimum age justification: Fossils were sampled from sediments that were collected from the outcrop that is part of the mid-Eocene to mid-Oligocene, 200 m thick succession that is overlayed by Pliocene and Pleistocene sediments (*127*). More detailed fossilized spicules fall within the Priabonian age 33.9–37.71 Ma. They derived from two middle units of the Blanche Point Formation: Gull Rock member (Mb.) and Perkana Member.

Node 94 (fig. S18): Phorbas | 37.8 Ma
Fossil Taxon Specimen: *Crellastrina* sp. ZPAL Pf.26, Upper Eocene units sampled from Doyle Road, Princess Royal, and the Hamersley River glauconitic and spiculitic marls and limestones. Eastern South Australia.
Phylogenetic justification: Spicules of the fossils resemble those of recent *Crellastrina alecto* Topsent, 1898 [described as Yvesia; family Crellidae (see Fig. 22D)] (*126*), which belongs to the family Crellidae and order Poecilosclerida. Notably, it is suggested that based on the spicules it might be that there is no recent equivalent representative of this fossil, but it has been hypothesized that the spicules still resemble those of *Crellastrina* the most.
Minimum age justification: Fossils were sampled from sediments that were collected from the outcrop that is part of the mid-Eocene to mid-Oligocene, 200 m thick succession that is overlayed by Pliocene and Pleistocene sediments (*127*). More detailed fossilized spicules fall within the Priabonian age 33.9–37.71 Ma. They derived from two middle units of the Blanche Point Formation: Gull Rock member (Mb.) and Perkana Member.

Node 103 (fig. S18): Astrophorina | 199 Ma
Fossil Taxon Specimen: Tetractinellida, Astrophorina (isolated dichotriaenes) Gabbs Valley Range of west central Nevada, USA.
Phylogenetic justification: arrays of long, complete, and complex in situ dichotriaenes of astrophorin demosponge affinity, see Fig. 6 in (*128*).
Minimum age justification: Fossils were found in the stratigraphic Unit of the Ferguson Hill Member of the Sunrise Formation within the Chert-dominated interval (24 to 55 m) which is of Hettangian and lower Sinemurian stages (199.3 Ma).

Node 107 (fig. S18): Crown Geodiidae | 166.1-163.5 Ma
Fossil Taxon Specimen: *Geoditesia jordaniensis*, Holotype ESH 2009 I 34, a Callovian (Middle Jurassic) fossil from north-western Jordan.
Phylogenetic justification: well-preserved specimens of an articulated sponge belonging to Geodia due to the consisting of a homogeneous mass of intermingled spicules, mainly oxeas and triaenes; sterrasters are also present; inter structure is however, chaotic, but nevertheless, seem to represent recent morphological features of the genus *Geodia* (*129*). We note that Cardenas (*94*) has questioned the affiliation of this fossils, and we performed a sensitivity test where the calibrations where relaxed to consider this view (see Supplementary Methods).
Minimum age justification: Fossil is found in the Tal el Dhahab, Callovian marls of Mughanniyya Formation, Jordan which is of Callovian age 166.1–163.5 Ma (Middle Jurassic).

Node 112 (fig. S18): Spongillida | 298 Ma
Fossil Taxon Specimen: Spongillida indet. PWL2004/5035a-LS, findspot Lemberg/Saar-Nahe Basin, layer 4, SW Germany.
Phylogenetic justification: morphological characters such as ordinary smooth monaxone spicules point to Spongillida. In addition, the fossils were from freshwater lake deposits placed far away from the sea, thus excluding a marine sponge assignment. However, gemmules and strongyles are absent, thus no fine scale classification can be made.
Minimum age justification: Permo-Carboniferous of Europe 298.9 Ma from the Saar-Nahe Basin (Stefanian C and Autunian) in south-west Germany. All fossils originated from freshwater lake deposits(*130*).

Node 128 (fig. S18): Hexactinellida | 445 Ma
Fossil Taxon Specimen: *Matteolaspongia hemiglobosa* (Holotype: NIGP168221). Found in the Anji Biota of the Wenchang Formation, middle to late *M. persculptus* Biozone, Hirnantian. Zhejiang Province, South China.

Phylogenetic justification: Based on morphological characterization (hypodermal pentactine prostalia, partly diactin-based skeleton) is fully consistent with a stem-Rossellidae interpretation, thus falls within hexactinellid sponges.
Minimum age justification: The fossil occurs in the latest Ordovician, the Hirnantian Age (445.2–443.8), more precisely dated to 444 Ma (*14*).

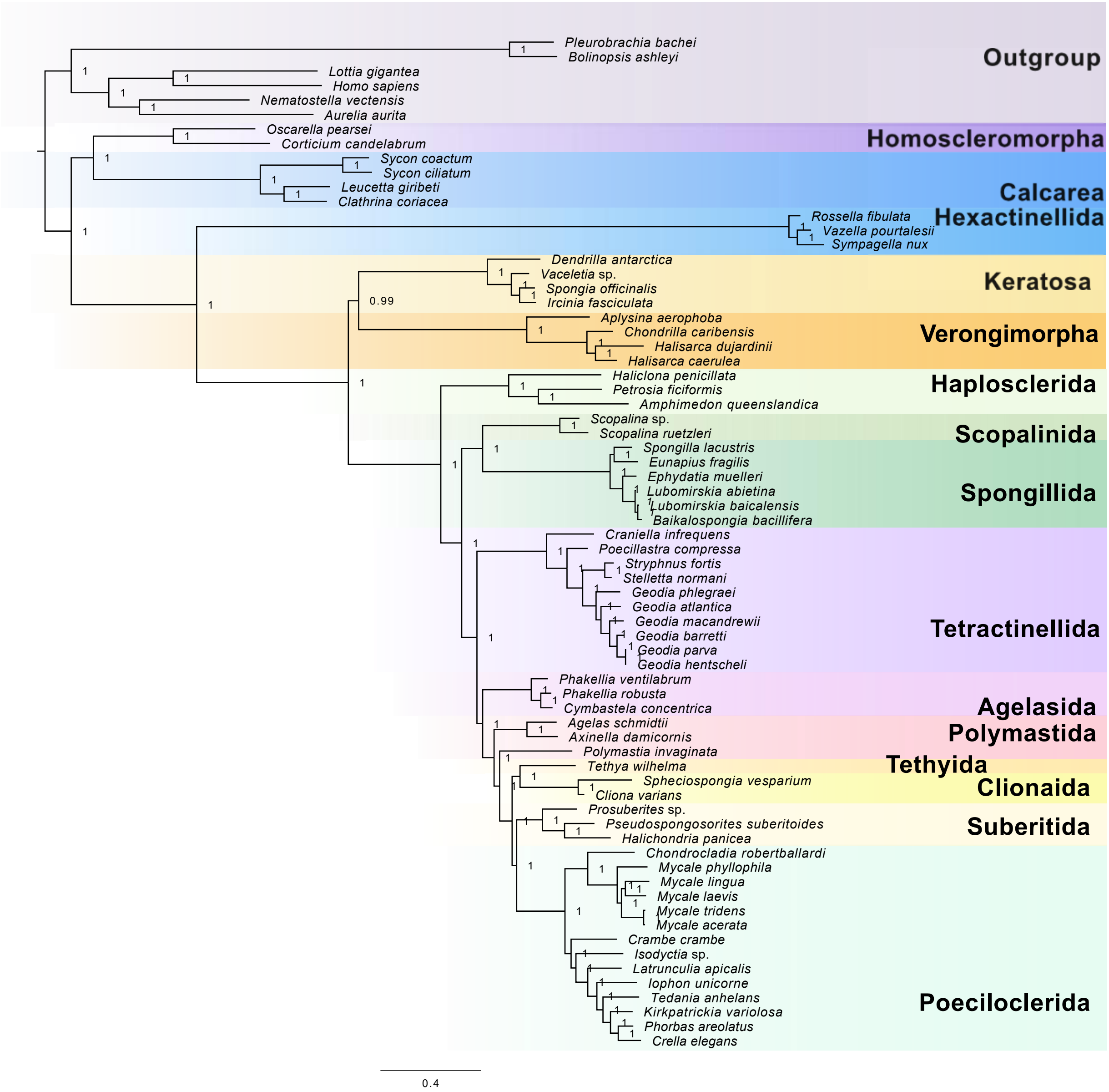

Fig. S1. Phylogenomic tree obtained with Phylobayes using CAT-Poisson. Number on the nodes indicate the posterior probability values. Convergence was check with bpcomp (maxdiff = 0.0018315) and tracecomp (minimal ESS 61 and maximum real_diff= 0.224488).

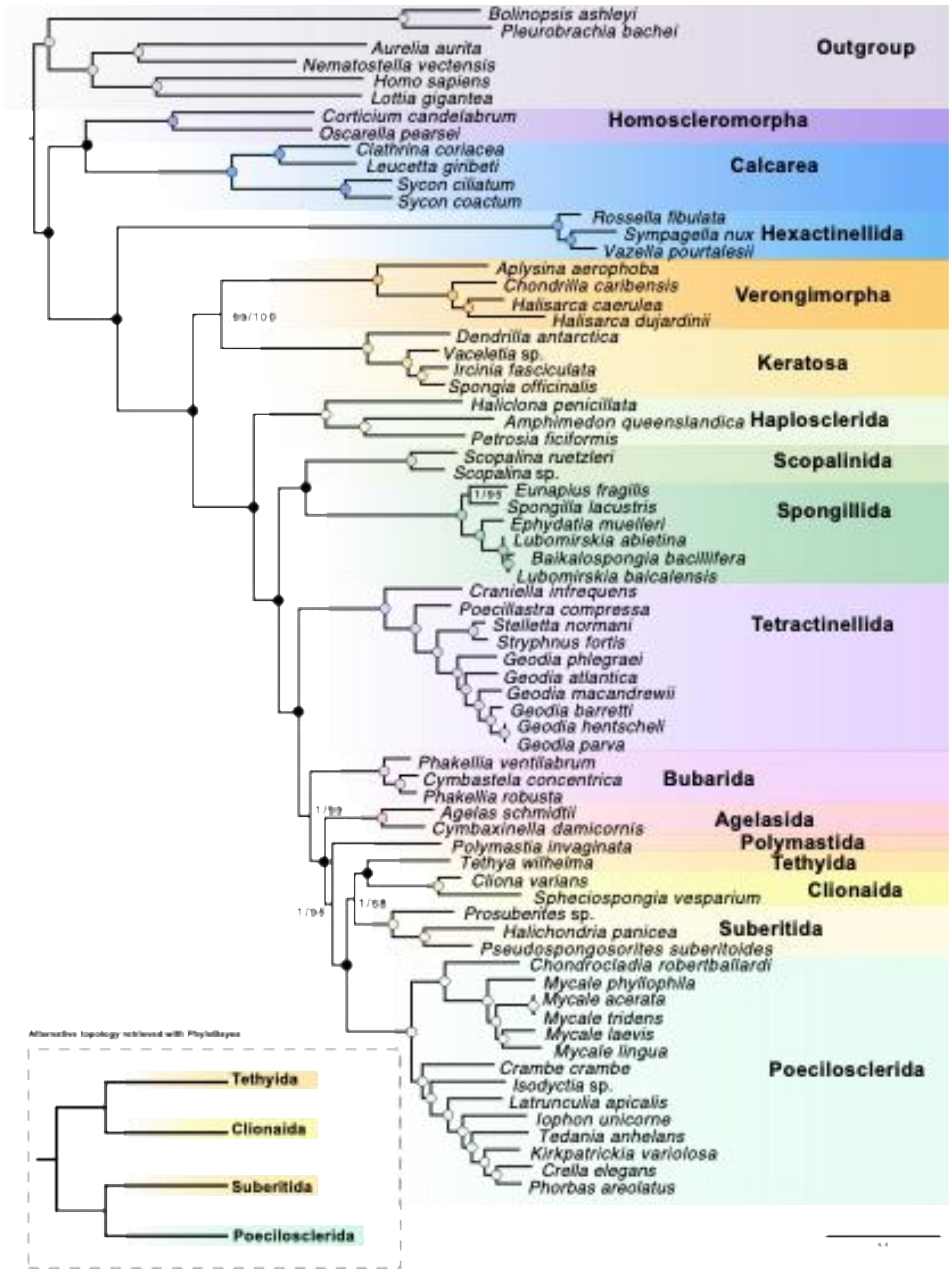

Bolinopsis ashleyi
Pleurobrachia bachei
Aurelia aurita
Nematostella vectensis
Homo sapiens
Lottia gigantea
Outgroup
Corticium candelabrum
Oscarella pearsei
Homoscleromorpha
Clathrina coriacea
Leucetta giribeti
Sycon ciliatum
Sycon coactum
Calcarea
Rossella fibulata
Sympagella nux
Vazella pourtalesii
Hexactinellida
Aplysina aerophoba
Chondrilla caribensis
Halisarca caerulea
Halisarca dujardinii
Verongimorpha
Dendrilla antarctica
Vaceletia sp.
Ircinia fasciculata
Spongia officinalis
Keratosa
Haliclona penicillata
Amphimedon queenslandica
Petrosia ficiformis
Haplosclerida
Scopalina ruetzleri
Scopalina sp.
Scopalinida
Eunapius fragilis
Spongilla lacustris
Ephydatia muelleri
Lubomirskia abietina
Baikalospongia bacillifera
Lubomirskia baicalensis
Spongillida
Craniella infrequens
Poecillastra compressa
Stelletta normani
Stryphnus fortis
Geodia phlegraei
Geodia atlantica
Geodia macandrewii
Geodia barretti
Geodia hentscheli
Geodia parva
Tetractinellida
Phakellia ventilabrum
Cymbastela concentrica
Phakellia robusta
Bubarida
Agelas schmidtii
Cymbaxinella damicornis
Agelasida
Polymastia invaginata
Polymastida
Tethya wilhelma
Tethyida
Cliona varians
Spheciospongia vesparium
Clionaida
Prosuberites sp.
Halichondria panicea
Pseudospongosorites suberitoides
Suberitida
Chondrocladia robertballardi
Mycale phyllophila
Mycale acerata
Mycale tridens
Mycale laevis
Mycale lingua
Crambe crambe
Isodyctia sp.
Latrunculia apicalis
Iophon unicorne
Tedania anhelans
Kirkpatrickia variolosa
Crella elegans
Phorbas areolatus
Poecilosclerida
Tethyida
Clionaida
Suberitida
Poecilosclerida

Fig. S2. Maximum likelihood topology compared against the results of the Bayesian analysis. Numbers on the nodes indicate Posterior Probabilities (From the Bayesian tree in Fig. 1 and S1) and bootstrap support (ML) respectively. Fully supported nodes from both analyses are represented by full circles. The grey box on the left reports the alternative topology for Tethyida, Clionaida, Suberitida and Poecilosclerida from the Bayesian analysis of Fig. 1 and S1 for direct comparison.

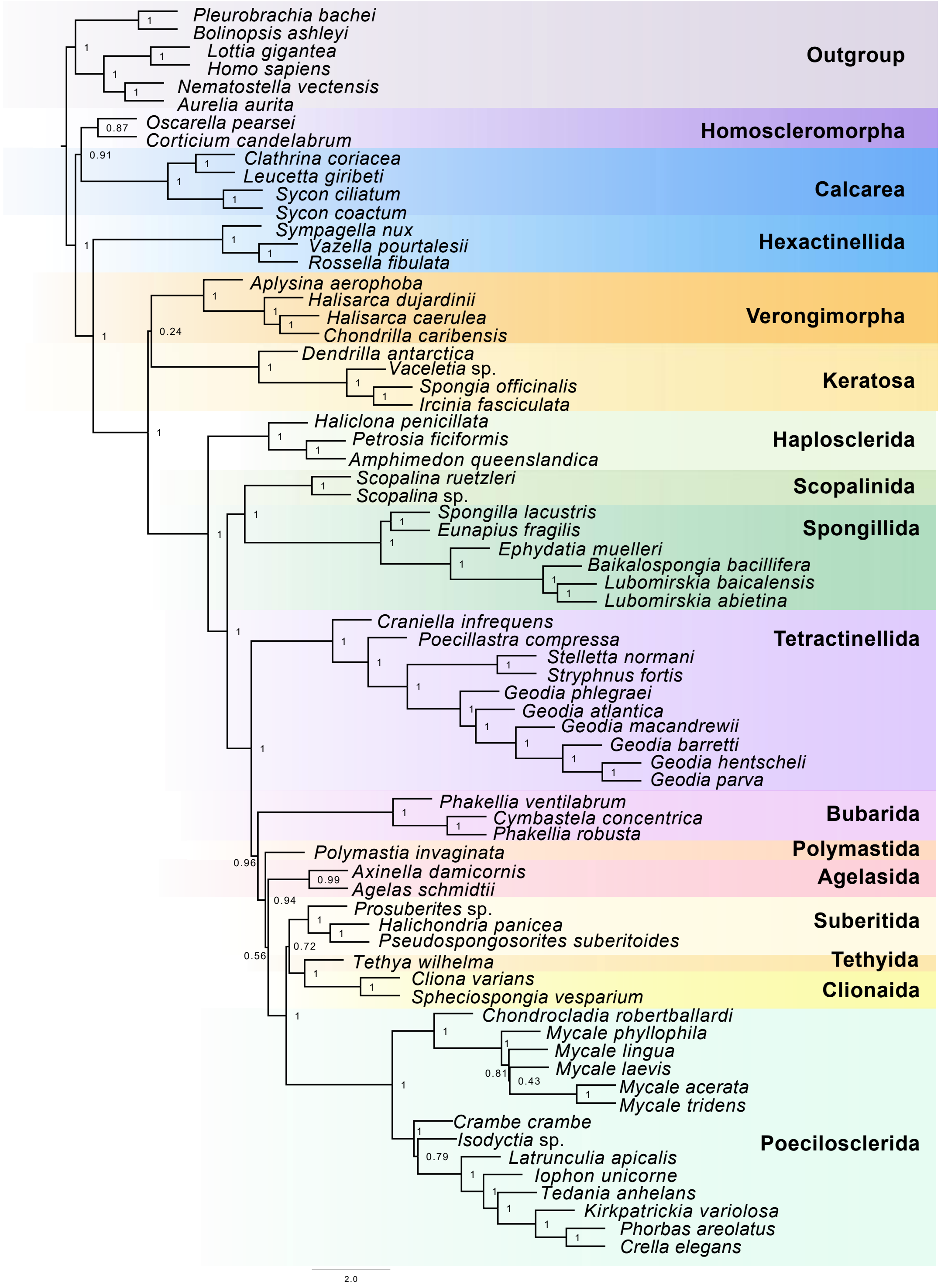

Pleurobrachia bachei
Bolinopsis ashleyi
Lottia gigantea
Homo sapiens
Nematostella vectensis
Aurelia aurita
Outgroup
Oscarella pearsei
Corticium candelabrum
Homoscleromorpha
Clathrina coriacea
Leucetta giribeti
Sycon ciliatum
Sycon coactum
Calcarea
Sympagella nux
Vazella pourtalesii
Rossella fibulata
Hexactinellida
Aplysina aerophoba
Halisarca dujardinii
Halisarca caerulea
Chondrilla caribensis
Verongimorpha
Dendrilla antarctica
Vaceletia sp.
Spongia officinalis
Ircinia fasciculata
Keratosa
Haliclona penicillata
Petrosia ficiformis
Amphimedon queenslandica
Haplosclerida
Scopalina ruetzleri
Scopalina sp.
Scopalinida
Spongilla lacustris
Eunapius fragilis
Ephydatia muelleri
Baikalospongia bacillifera
Lubomirskia baicalensis
Lubomirskia abietina
Spongillida
Craniella infrequens
Poecillastra compressa
Stelletta normani
Stryphnus fortis
Geodia phlegraei
Geodia atlantica
Geodia macandrewii
Geodia barretti
Geodia hentscheli
Geodia parva
Tetractinellida
Phakellia ventilabrum
Cymbastela concentrica
Phakellia robusta
Bubarida
Polymastia invaginata
Polymastida
Axinella damicornis
Agelas schmidtii
Agelasida
Prosuberites sp.
Halichondria panicea
Pseudospongosorites suberitoides
Suberitida
Tethya wilhelma
Tethyida
Cliona varians
Spheciospongia vesparium
Clionaida
Chondrocladia robertballardi
Mycale phyllophila
Mycale lingua
Mycale laevis
Mycale acerata
Mycale tridens
Crambe crambe
Isodyctia sp.
Latrunculia apicalis
Iophon unicorne
Tedania anhelans
Kirkpatrickia variolosa
Phorbas areolatus
Crella elegans
Poecilosclerida
0.87
0.91
0.24
0.96
0.99
0.94
0.72
0.56
0.81
0.43
0.79
2.0

Fig. S3. Phylogenetic topology of Porifera inferred with ASTRAL. Number on the nodes indicates the coalescent branch support values.

A

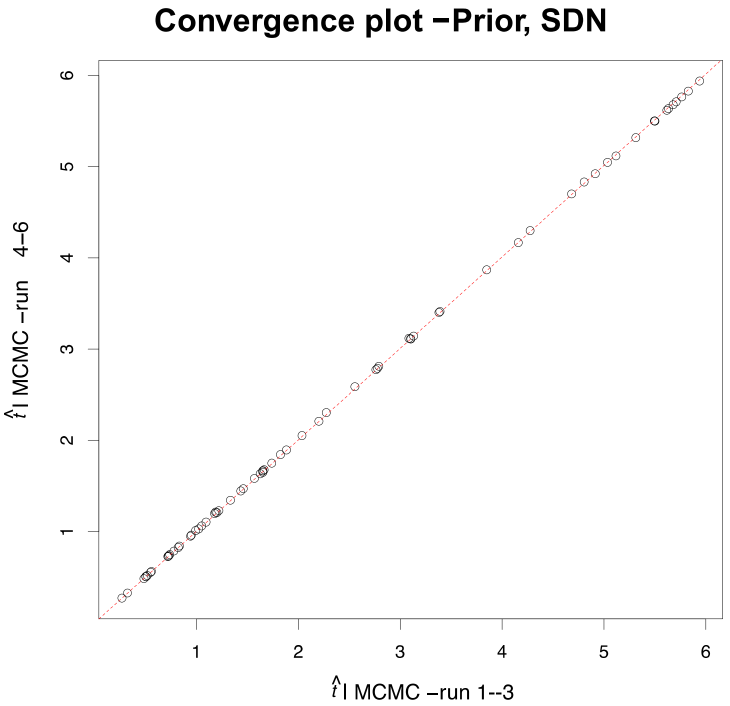

B

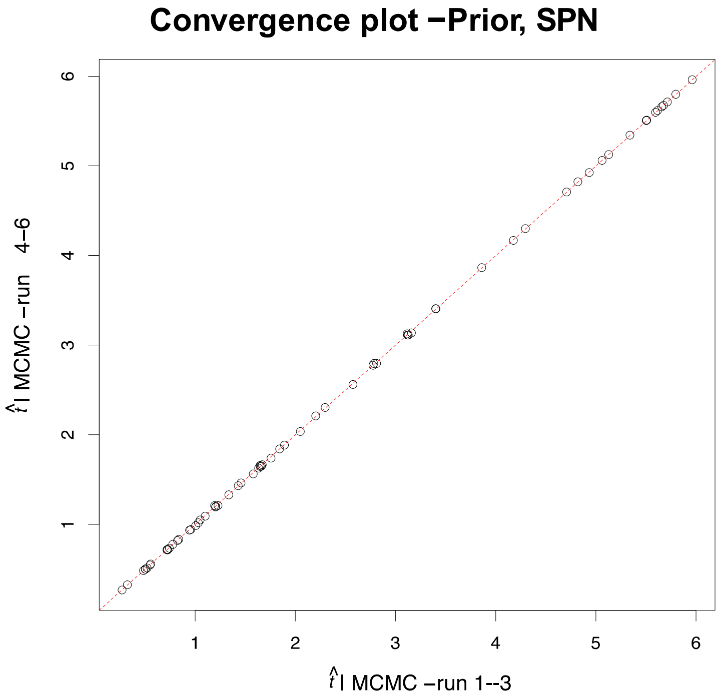

Figure S4 Prior convergence plot for time calibration analyses Convergence plot for the prior estimation of the two calibration strategies SPN and SDN

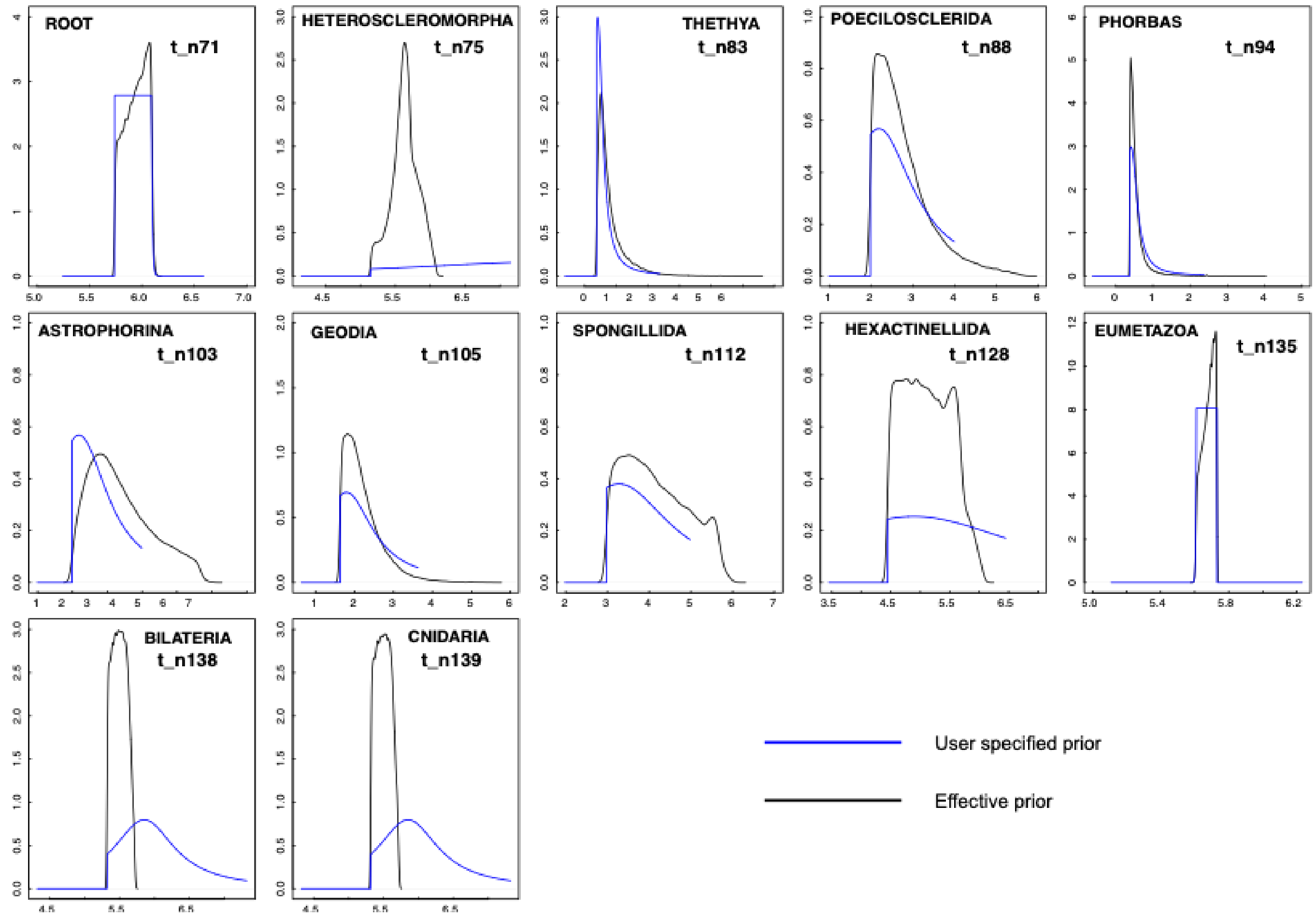

Figure S5 Prior selection plots. Prior distribution plots of the calibration used for the divergence time estimates, comparing the user-specified prior (blue line) and the effective prior (black line) selected by MCMCTree.

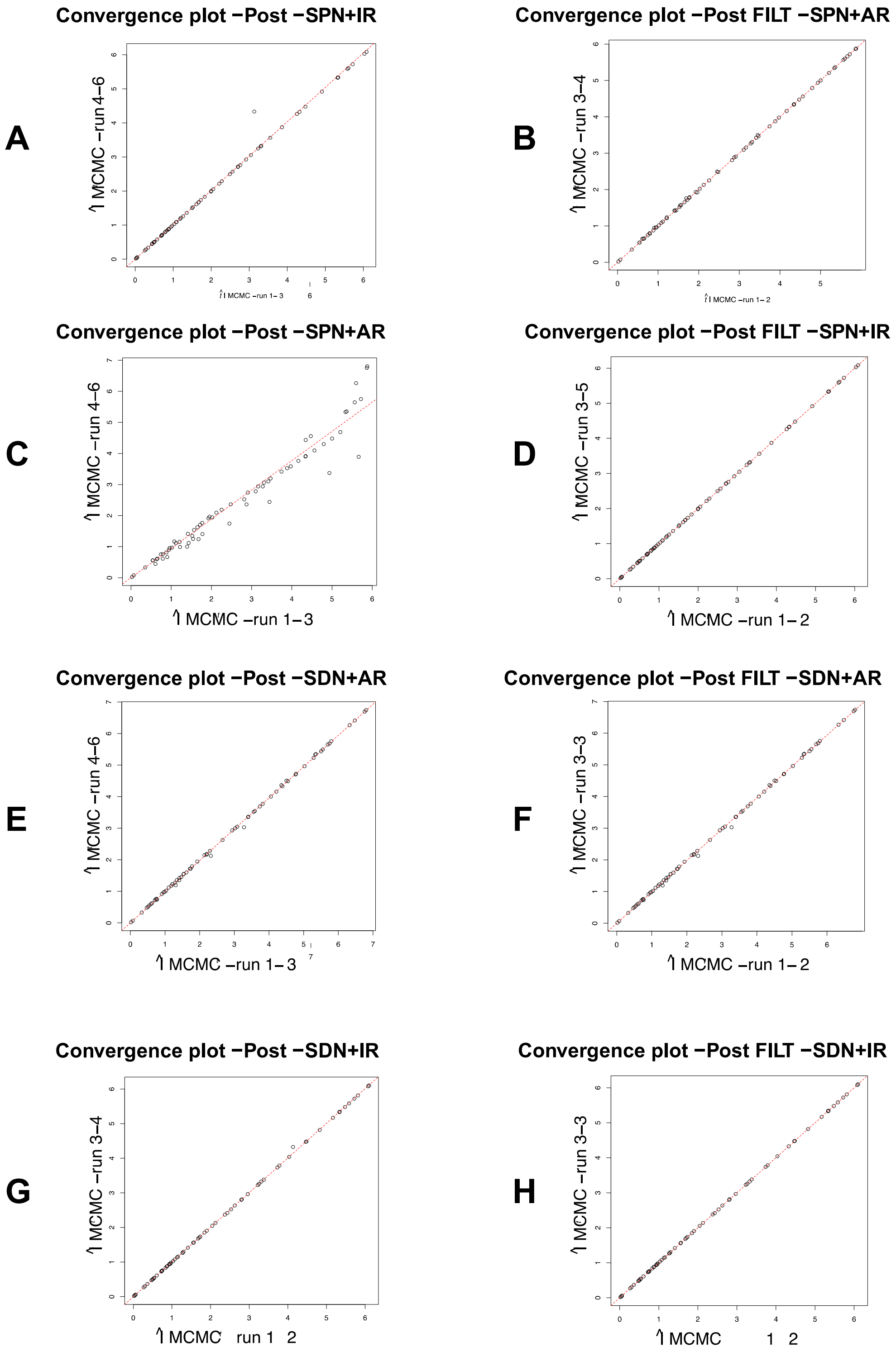
A
Convergence plot −Post −SPN+IR
MCMC −run 4−6
MCMC −run 1−3
B
Convergence plot −Post FILT −SPN+AR
MCMC −run 3−4
MCMC −run 1−2
C
Convergence plot −Post −SPN+AR
MCMC −run 4−6
MCMC −run 1−3
D
Convergence plot −Post FILT −SPN+IR
MCMC −run 3−5
MCMC −run 1−2
E
Convergence plot −Post −SDN+AR
MCMC −run 4−6
MCMC −run 1−3
F
Convergence plot −Post FILT −SDN+AR
MCMC −run 3−3
MCMC −run 1−2
G
Convergence plot −Post −SDN+IR
MCMC −run 3−4
MCMC run 1 2
H
Convergence plot −Post FILT −SDN+IR
MCMC −run 3−3
MCMC 1 2

Figure S6 Posterior convergence plots check Convergence plots of the posterior time estimation for the two calibration strategies pre and post filtering for the chains flagged as problematic by the inhouse script MCMC diagnostics (see Methods).

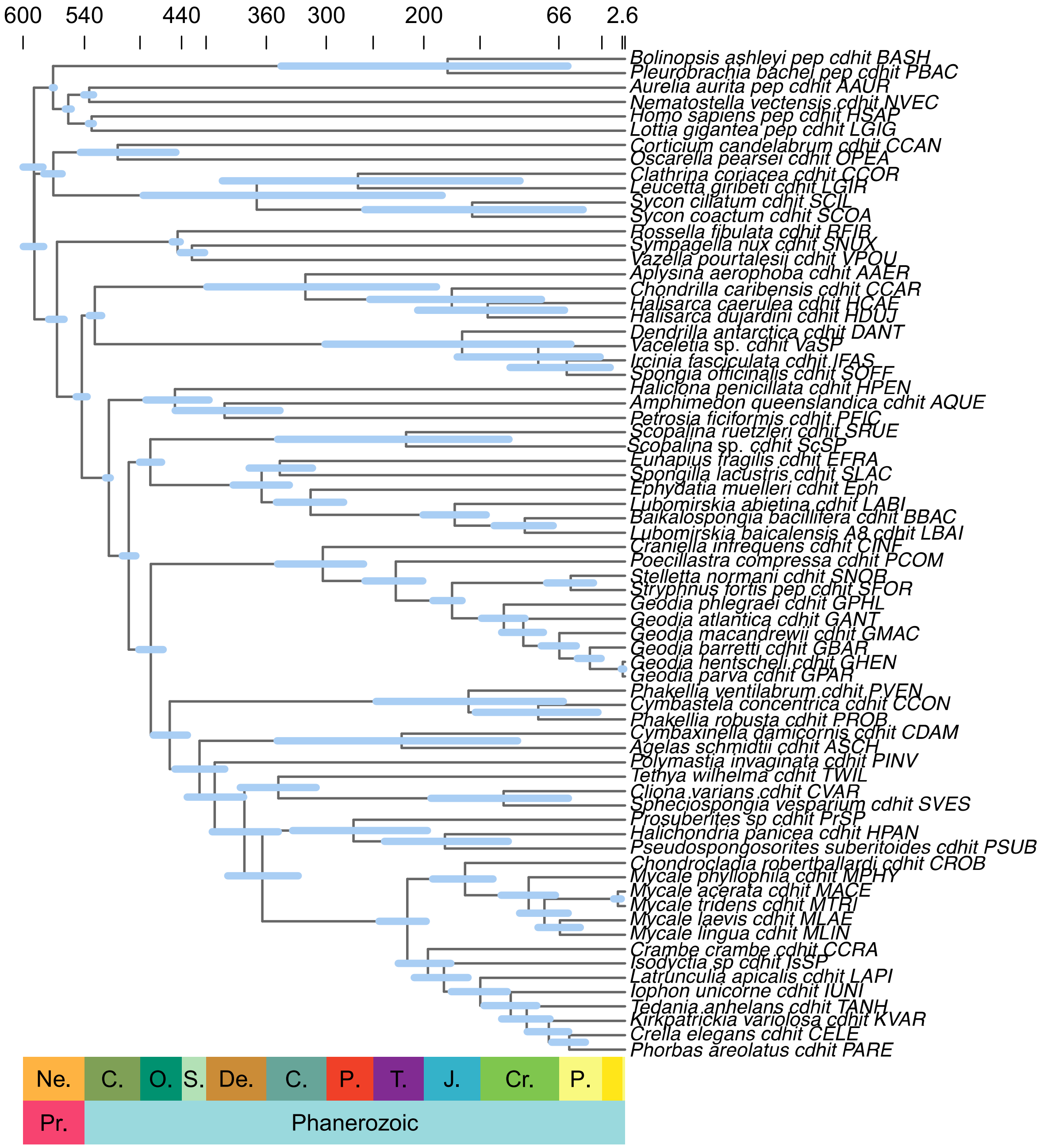

Figure S7 Porifera time-tree with posterior estimates using the SDN calibration strategy and the AR model in MCMCTree. Blue bars on nodes represent the 95% HPD. Time scale on x-axis in hundreds of millions of years. Abbreviation on the scale bar stands for Ne, Neoproterozoic; C, Cambrian; O, Ordovician; S, Silurian; D, Devonian; C, Carboniferous; P, Permian; Tr, Triassic; J, Jurassic; K, Cretaceous; Pe, Paleogene.

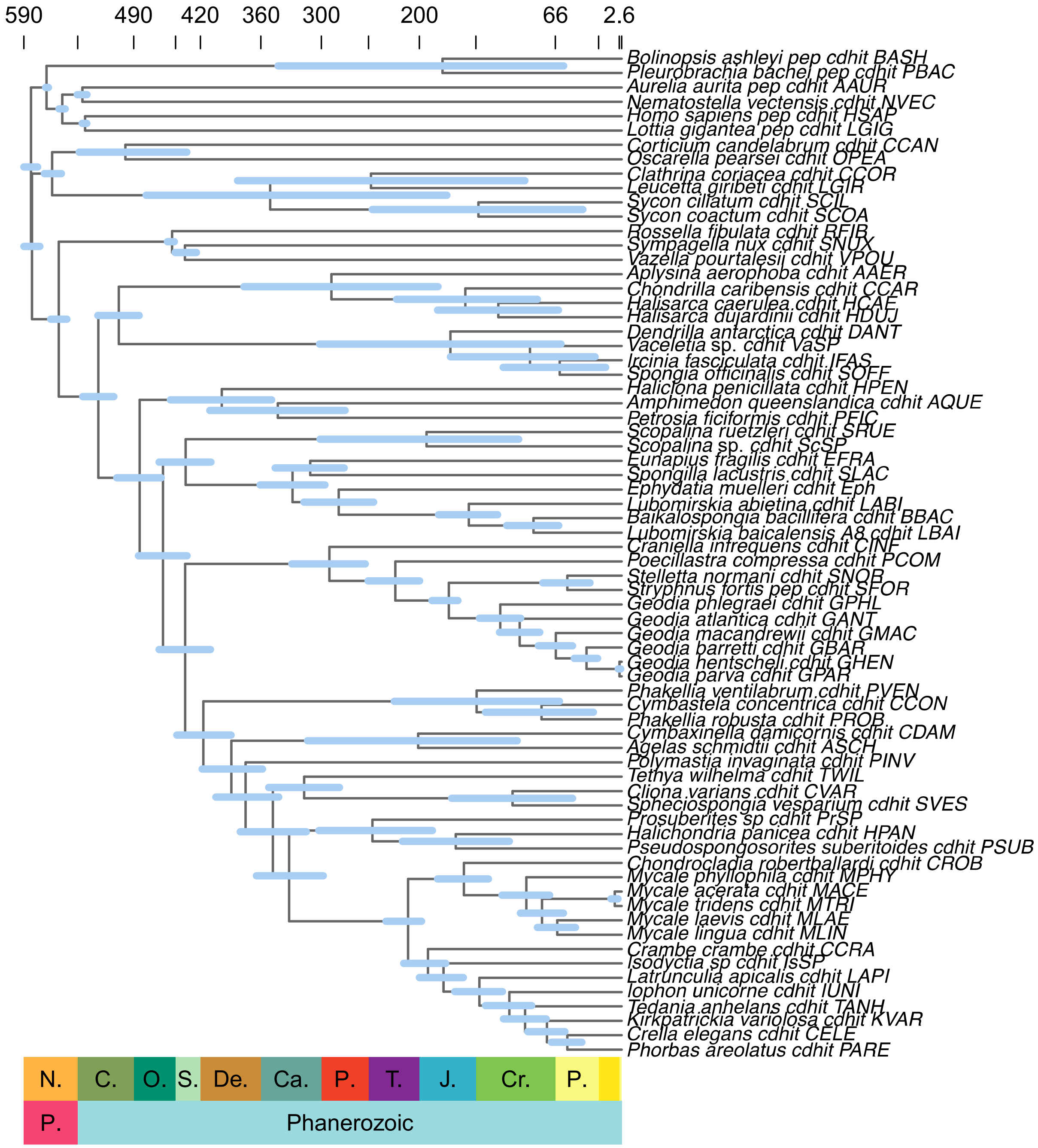

Figure S8 Porifera time-tree with posterior estimates using the SPN calibration strategy and the AR model in MCMCTree. Blue bars on nodes represent the 95% HPD. Time scale on x-axis in hundreds of millions of years. Abbreviation on the scale bar stands for Ne, Neoproterozoic; C, Cambrian; O, Ordovician; S, Silurian; D, Devonian; C, Carboniferous; P, Permian; Tr, Triassic; J, Jurassic; K, Cretaceous; Pe, Paleogene.

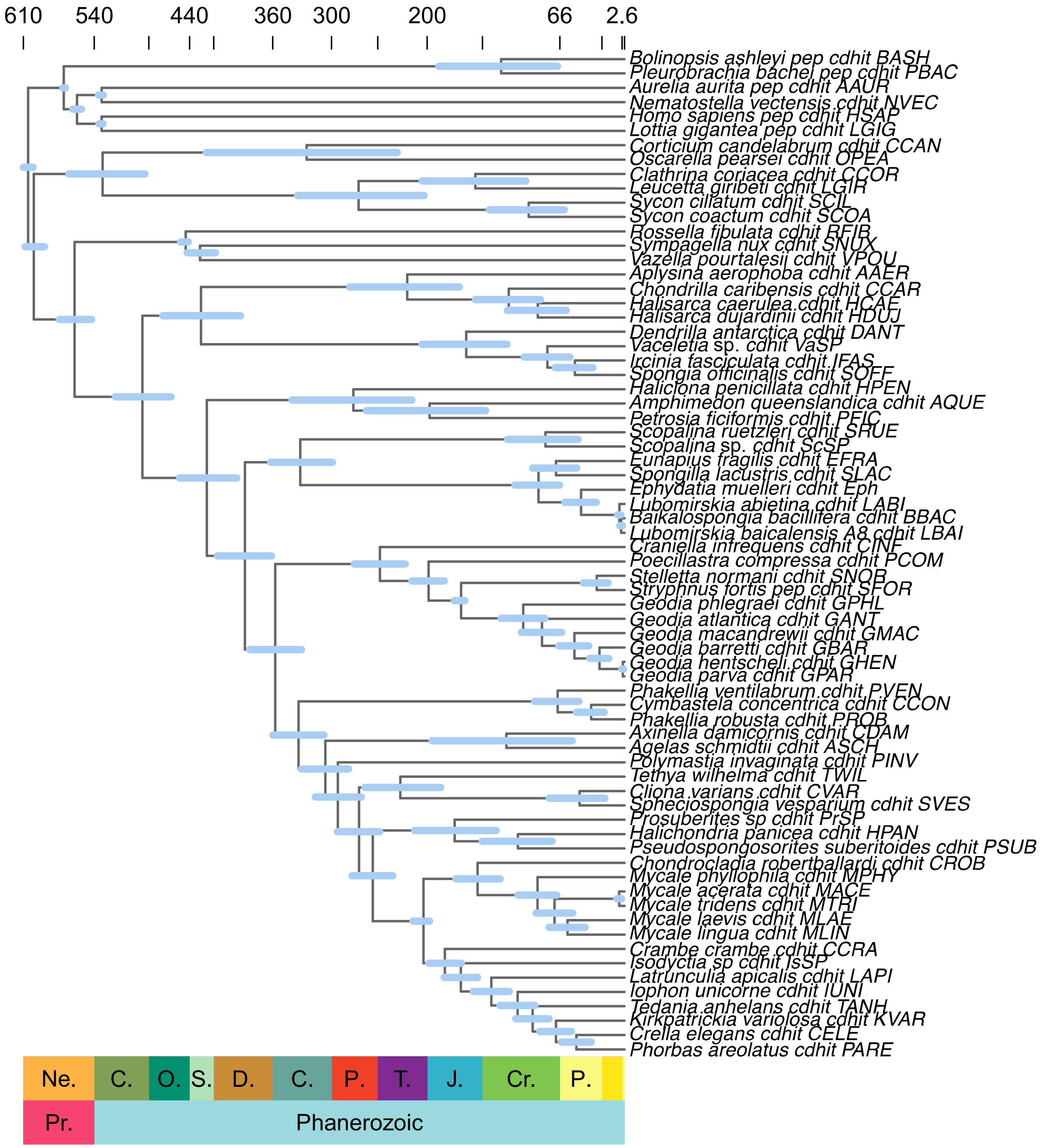

Figure S9 Porifera time-tree with posterior estimates using the SPN calibration strategy and the IR model in MCMCTree. Blue bars on nodes represent the 95% HPD. Time scale on x-axis in hundreds of millions of years. Abbreviation on the scale bar stands for Ne, Neoproterozoic; C, Cambrian; O, Ordovician; S, Silurian; D, Devonian; C, Carboniferous; P, Permian; Tr, Triassic; J, Jurassic; K, Cretaceous; Pe, Paleogene.

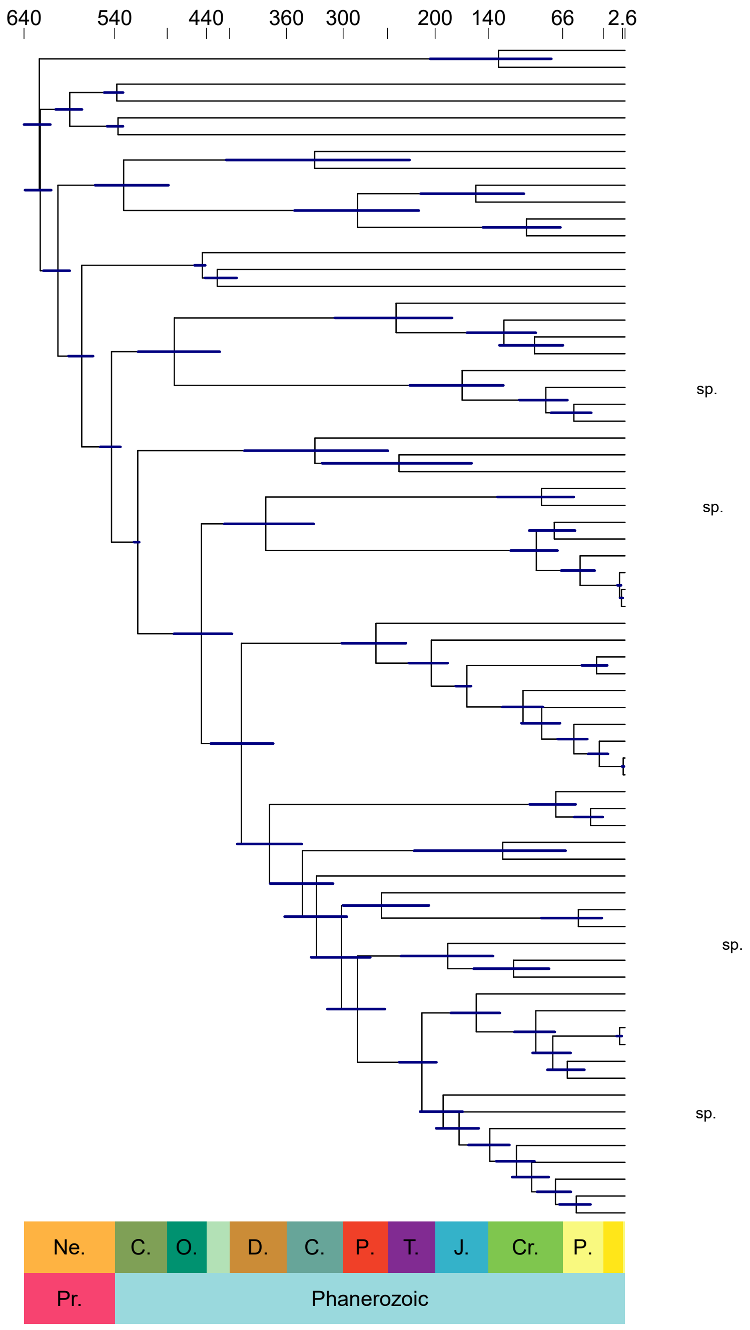

Figure S10 Ctenophora-sister hypotesis time tree under the IR model. Porifera time-tree with posterior estimates using the Ctenophora sister rooting strategy, the SDN calibration strategy and the IR model in MCMCTree. Blue bars on nodes represent the 95% HPD. Time scale on x-axis in hundreds of millions of years. Abbreviation on the scale bar stands for Ne, Neoproterozoic; C, Cambrian; O, Ordovician; S, Silurian; D, Devonian; C, Carboniferous; P, Permian; Tr, Triassic; J, Jurassic; K, Cretaceous; Pe, Paleogene

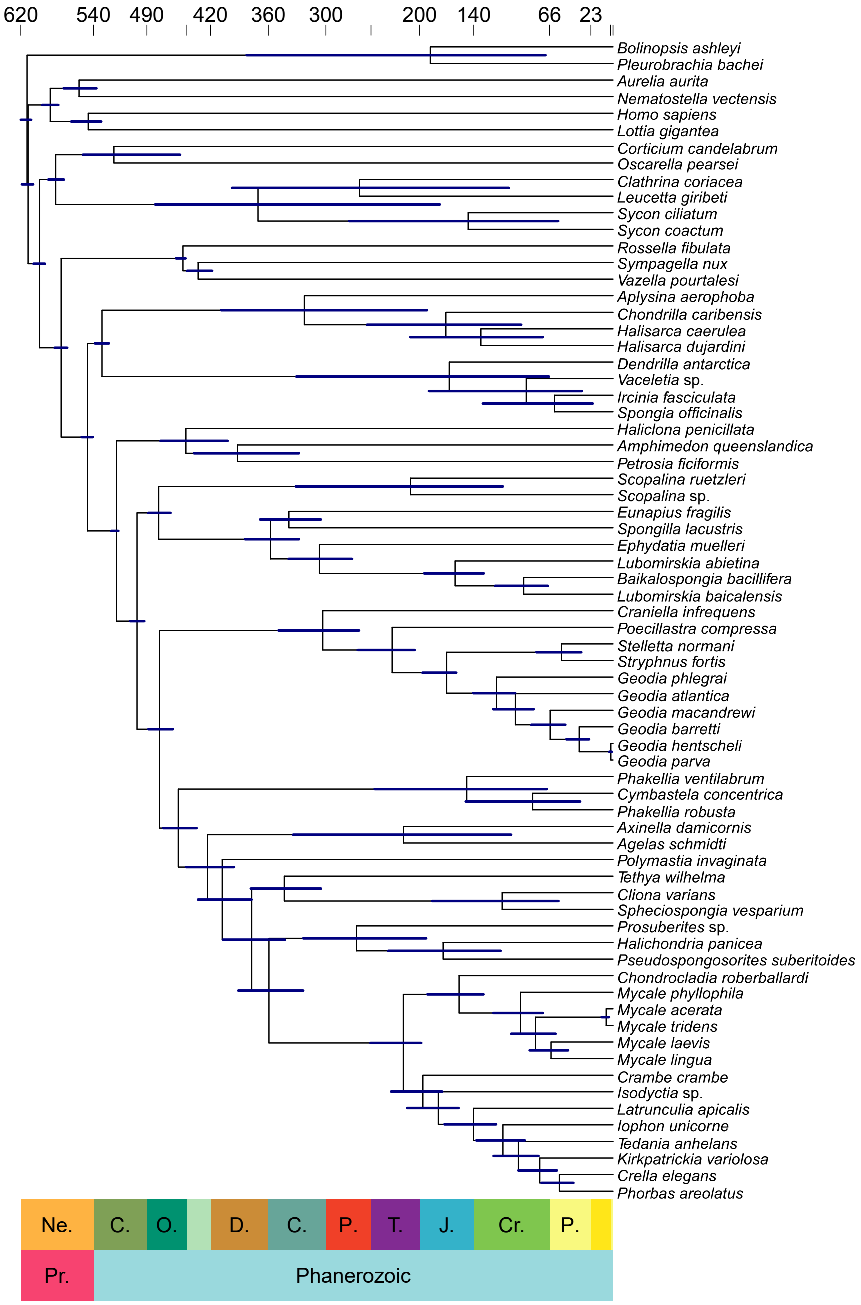

Figure S11 Ctenophora-sister hypotesis time tree under the AR model. Porifera time-tree with posterior estimates using the Ctenophora sister rooting strategy, the SDN calibration strategy and the AR model in MCMCTree. Blue bars on nodes represent the 95% HPD. Time scale on x-axis in hundreds of millions of years. Abbreviation on the scale bar stands for Ne, Neoproterozoic; C, Cambrian; O, Ordovician; S, Silurian; D, Devonian; C, Carboniferous; P, Permian; Tr, Triassic; J, Jurassic; K, Cretaceous; Pe, Paleogene

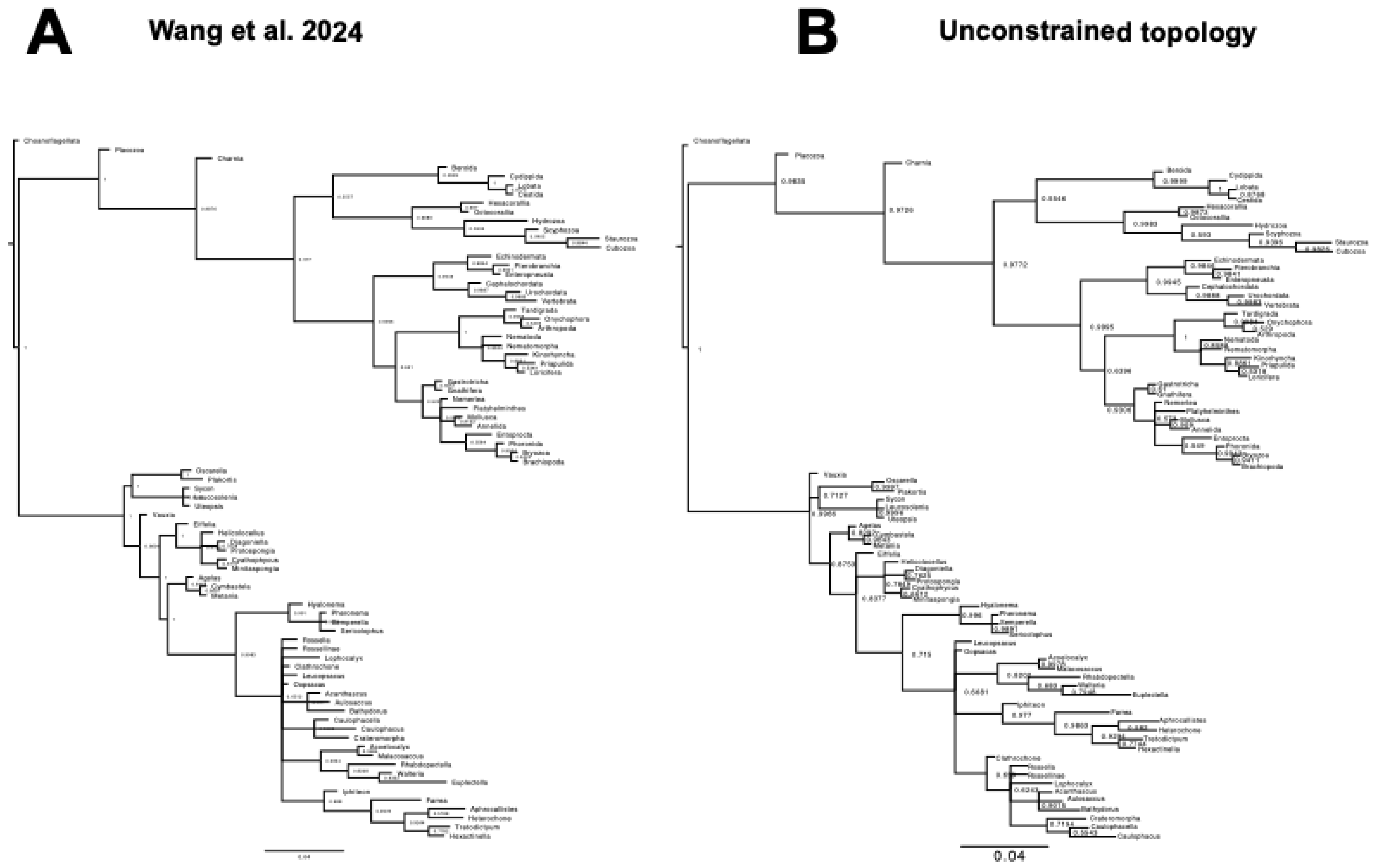
A
Wang et al. 2024
B
Unconstrained topology
0.04

Figure S12 Competing topologies used in the ancestral state estimations analysis (A) Results of MrBayes analysis where extant taxa are constrained to follow results of our phylogenomic analyses and fossil taxa are constrained to belong to the same clade where they were found in Wang et al. (42) original study. **(B)** Results of MrBayes analyses where the extant taxa were constrained to follow the results of our phylogenomic analyses and the fossil taxa were free to place in their optimal (unconstrained) position in the tree topology.

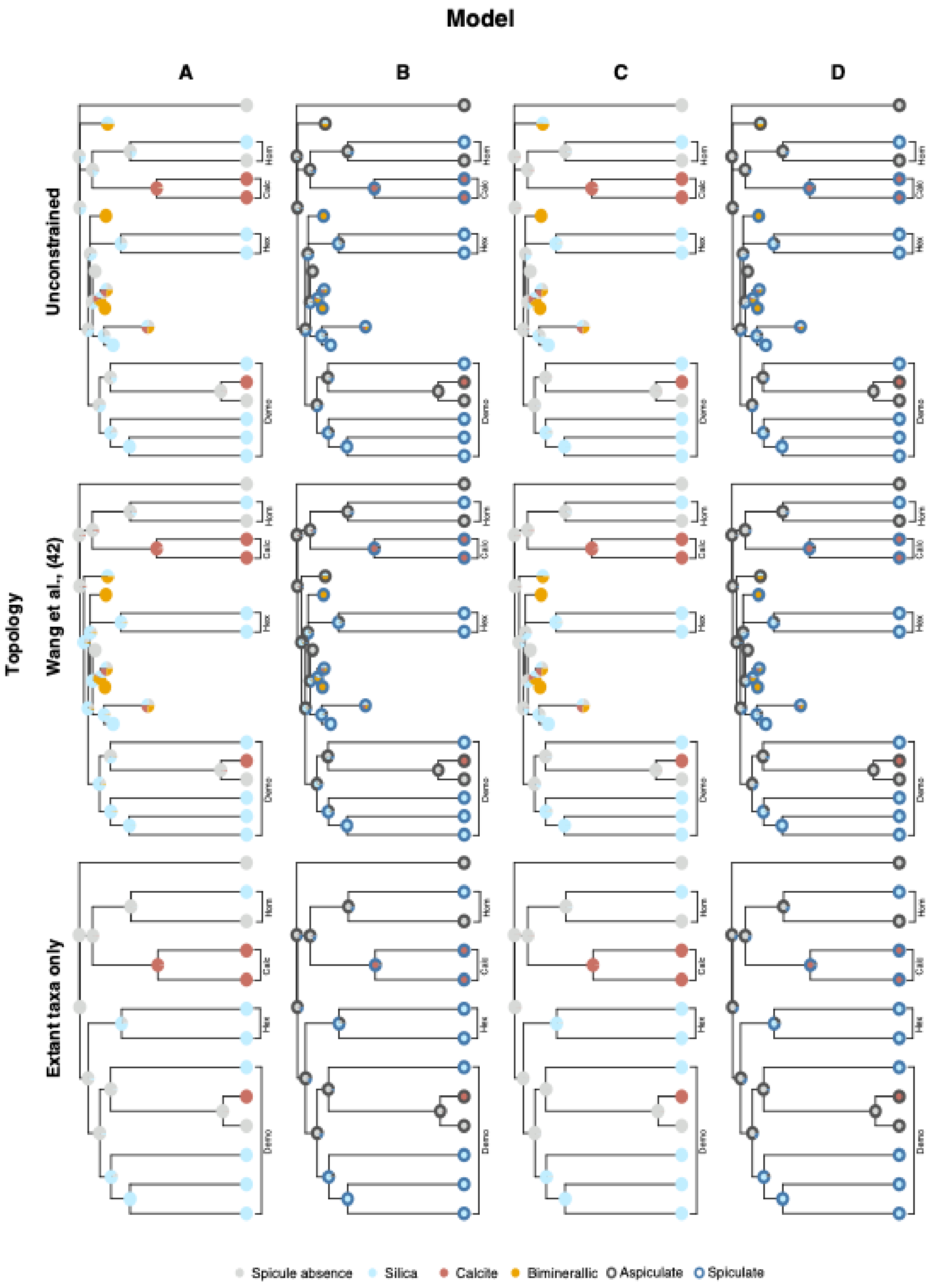
Model
A
B
C
D
Topology
Unconstrained
Wang et al., (42)
Extant taxa only
Hom
Calc
Hex
Demo
Spicule absence
Silica
Calcite
Bimineralllic
Aspiculate
Spiculate

Figure S13 Simplified summary results of the ancestral state estimations. Horizontally the trees are grouped by the topologies, and vertically by models. The legend depicts each color assigned to mineralogy. Grey absence of biomineralisation, light blue for siliceous mineralogy, red for calcareous mineralogy, and orange for biminerallic spicules. Outside circles represent the presence (blue) or absence (dark grey) of any type of spicules. Here we show only mineralogies, but not biochemical differences in the developmental of spicules of a specific type. To see full results please refer to the Figshare repository (https://doi.org/10.6084/m9.figshare.28574570).

Prior

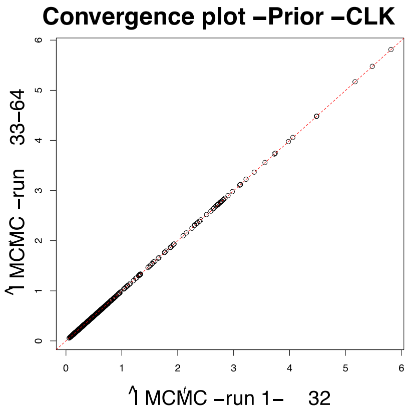

Posterior

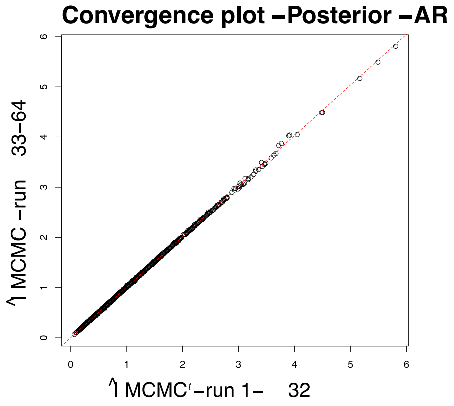

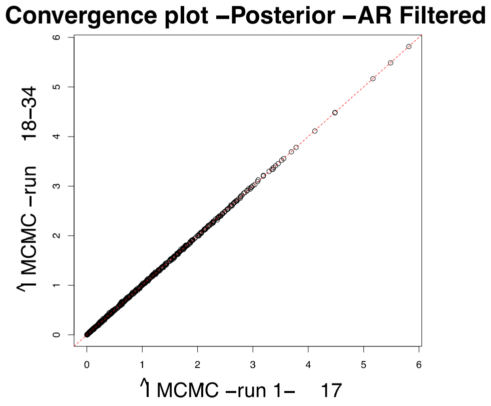

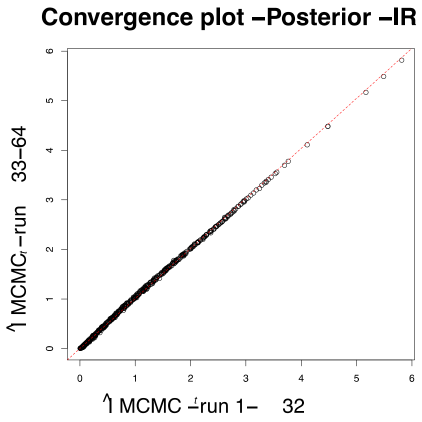

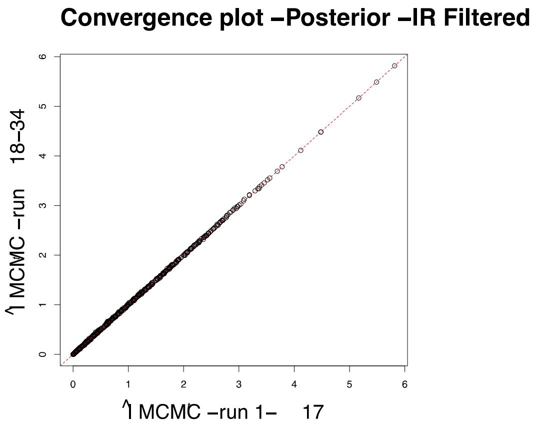

Figure S14 Convergence plots for the COI topology Convergence plots of the posterior time estimation for the COI topology used for diversification rates analyses. Pre and post filtering for the chains flagged as problematic by our in-house script MCMC diagnostics.

Prior

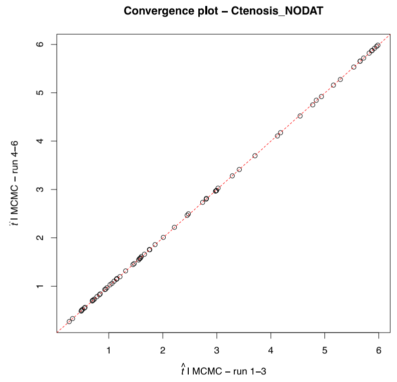

Posterior

A

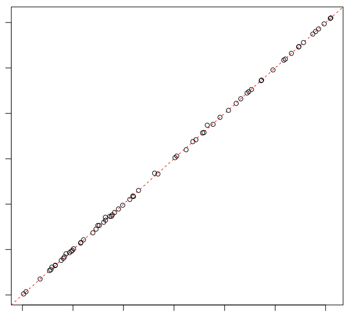

B

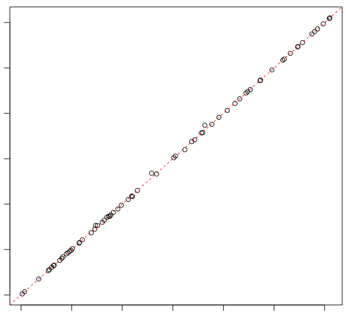

C

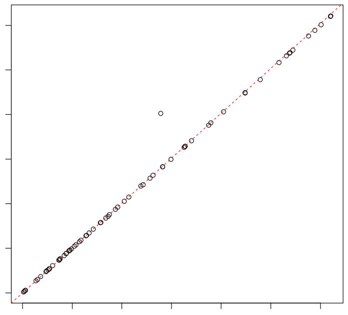

D

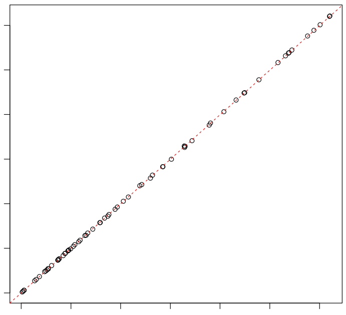

Figure S15 Convergence plots of the posterior time estimation for the Ctenophora-sister topology test. Pre and post filtering for the chains flagged as problematic by our in-house script MCMC diagnostics.

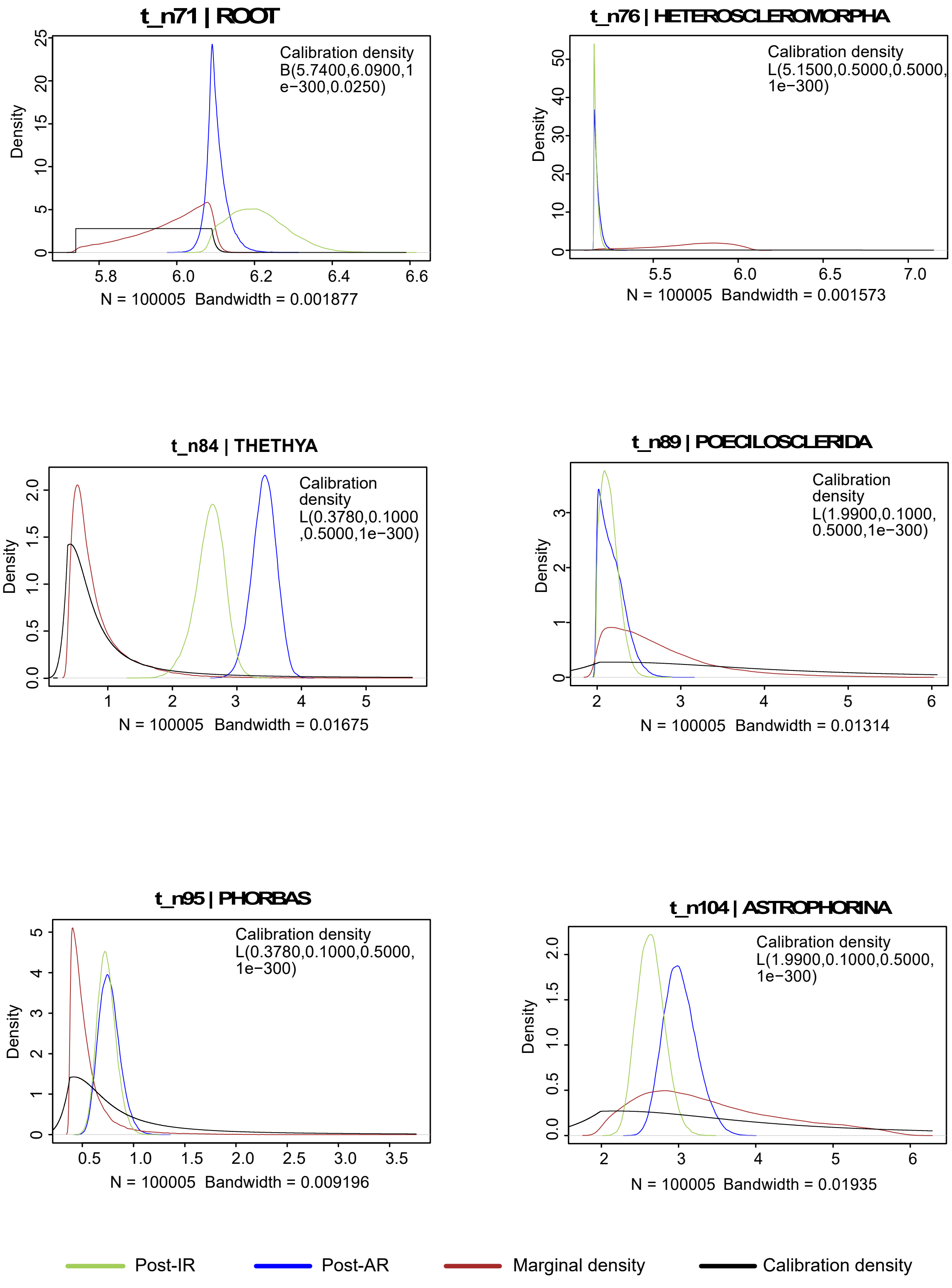
t_n71 | ROOT
Calibration density
B(5.7400,6.0900,1
e−300,0.0250)
Density
N = 100005 Bandwidth = 0.001877
t_n76 | HETEROSCLEROMORPHA
Calibration density
L(5.1500,0.5000,0.5000,
1e−300)
Density
N = 100005 Bandwidth = 0.001573
t_n84 | THETHYA
Calibration
density
L(0.3780,0.1000
,0.5000,1e−300)
Density
N = 100005 Bandwidth = 0.01675
t_n89 | POECILOSCLERIDA
Calibration
density
L(1.9900,0.1000,
0.5000,1e−300)
Density
N = 100005 Bandwidth = 0.01314
t_n95 | PHORBAS
Calibration density
L(0.3780,0.1000,0.5000,
1e−300)
Density
N = 100005 Bandwidth = 0.009196
t_n104 | ASTROPHORINA
Calibration density
L(1.9900,0.1000,0.5000,
1e−300)
Density
N = 100005 Bandwidth = 0.01935
Post-IR
Post-AR
Marginal density
Calibration density

Figure S16 Density plots for each calibrated node in the Ctenophora-sister sensitivity tests. Each plot reports a comparison, for each node, the user-specified prior (black line), the effective priors (green and blue line), and the marginal densitiyes selected by MCMCTree.

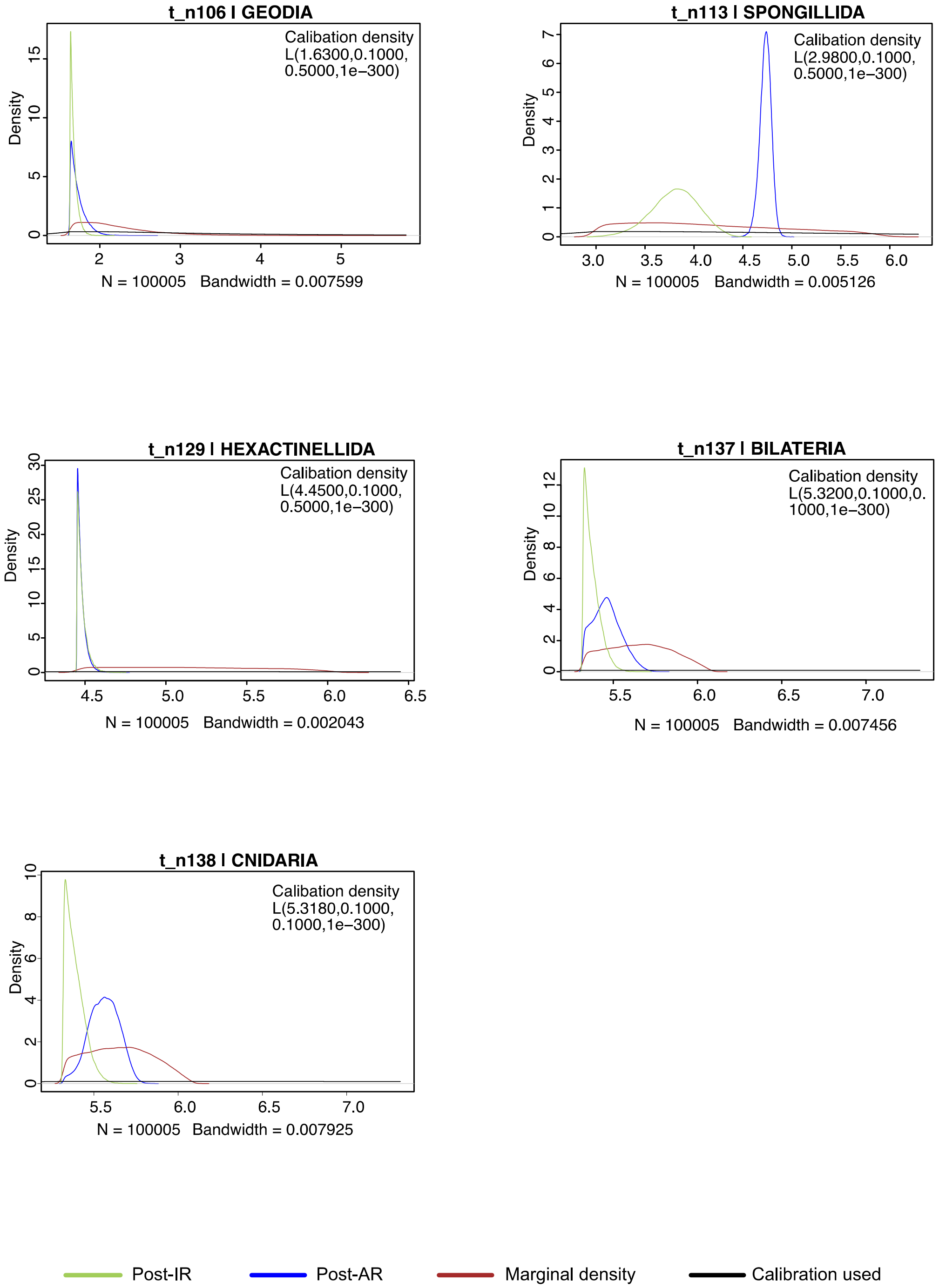

Figure S17 Density plots for each calibrated node in the Ctenophora-sister sensitivity tests. Each plot reports a comparison, for each node, the user-specified prior (black line), the effective priors (green and blue line), and the marginal densitiyes selected by MCMCTree.

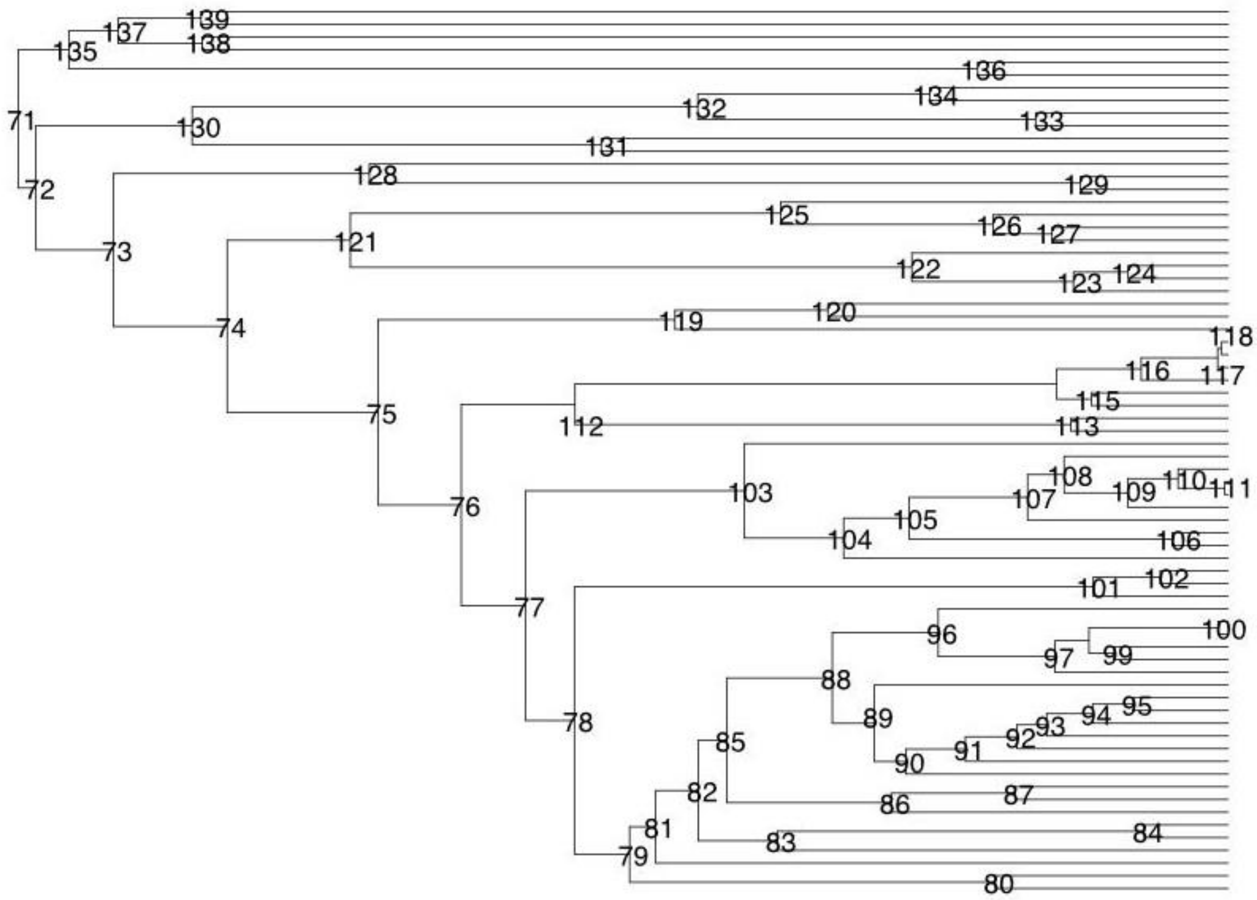

Figure S18 numerical labels identifying nodes in the Bayesian phylogeny. This tree depicts the Bayesian phylogeny from Fig. 1 and fig. S1. Each internal node in this tree has been given a numerical label to identify it. This labeling structure is used to identify the position of calibrated nodes.

Table S1. MCMC diagnostics for time densities obtained with MCMCtree when fixing the SDN tree topology, both when the target distribution was set to be the prior (no data, label "CLK") or the posterior (data were used, labels for relaxed-clock models are "AR" for autocorrelated rates and "IR" for independent rates sampled from a log-normal distribution).

SDN Strategy

	**Prior-CLK**	**Autocorrelated-AR**	**Independent-IR**
**tail-ESS times (median)**	30,865	1,063	3,374
**tail-ESS times (min)**	3,359	273	686
**tail-ESS times (max)**	53,466	23,413	28,471
**bulk-ESS times (median)**	33,260	667	1873
**bulk-ESS times (min)**	6,695	133	348
**bulk-ESS times (max)**	45,726	18,287	29,085
**Rhat min**	0.9999812	0.9999731	0.9999337
**Rhat max**	1.001506	1.038014	1.006146
**Med. number of samples per chain**	20,001	20,001	20,001
**Min. number of samples per chain**	20,001	20,001	20,001
**Max. number of samples per chain**	20,001	20,001	20,001
**Number of chains that were run**	6	6	6
**Number of chains kept after filters**	6	3	3
**Number of samples used to calculate tail-ESS and bulk-ESS**	120,006	60,003	60,003
**Total number of samples kept *post-hoc* analyses**	120,006	60,003	60,003

Table S2 MCMC diagnostics for time densities obtained with MCMCtree when fixing the SPN tree topology, both when the target distribution was set to be the prior (no data, label "CLK") or the posterior (data were used, labels for relaxed-clock models are "AR" for autocorrelated rates and "IR" for independent rates sampled from a log-normal distribution).

SPN strategy

	**Prior-CLK**	**Autocorrelated-AR**	**Independent-IR**
**tail-ESS times (median)**	30,662	1,348	6,014
**tail-ESS times (min)**	3,661	411	1555
**tail-ESS times (max)**	59,697	33,096	49,641
**bulk-ESS times (median)**	33,336	741	3197
**bulk-ESS times (min)**	6,451	214	768
**bulk-ESS times (max)**	58,044	29,179	46,930
**Rhat min**	0.999989	1.000045	0.9999561
**Rhat max**	1.000565	1.025086	1.005087
**Med. number of samples per chain**	20,001	20,001	20,001
**Min. number of samples per chain**	20,001	20,001	20,001
**Max. number of samples per chain**	20,001	20,001	20,001
**Number of chains that were run**	6	6	6
**Number of chains kept after filters**	6	4	5
**Number of samples used to calculate tail-ESS and bulk-ESS**	120,006	80,004	100,005
**Total number of samples kept *post-hoc* analyses**	120,006	80,004	100,005

Table S3 Mean posterior divergence times estimated with MCMCtree when the calibration strategy was SPN under both relaxed-clock models ("AR" for autocorrelated rates and "IR" for independent rates sampled from a log-normal distribution).

SPN-IR					SPN-AR				
	**Mean div. time**	**2.5% CI**	**97.5% CI**	**Priors**		**Mean div. time**	**2.5% CI**	**97.5% CI**	**Priors**
t_n71 ROOT	608.582	603.28	613.15	B(5.7400,6.0900,1e-300,0.0250)	T_n71 ROOT	587.641	581.39	595.25	B(5.7400,6.0900,1e-300,0.0250)
t_n72 PORIFERA	602.821	590.34	610.68	L(5.1500,0.5000,0.5000,1e-300)	t_n72 PORIFERA	586.586	578.79	594.59	L(5.1500,0.5000,0.5000,1e-300)
t_n73	561.092	542.68	575.87	0	t_n73	559.951	551.55	567.69	0
t_n74	491.825	462.67	519.67	0	t_n74	520.579	503.28	535.02	0
t_n75	426.204	396.53	454.95	0	t_n75	479.364	455.96	500.69	0
t_n76	387.255	359.79	414.91	0	t_n76	456.313	431.08	480.01	0
t_n77	356.451	330.46	383.55	0	t_n77	434.377	407.97	459.28	0
t_n78	332.509	307.43	359.25	0	t_n78	416.176	388.49	442.35	0
t_n79	305.303	282.35	330.66	0	t_n79	388.33	357.39	417.34	0
t_n80	120.702	57.97	202.37	0	t_n80	202.386	99.8	308.88	0
t_n81	292.378	270.1	317.26	0	t_n81	374.188	342.07	404.57	0
t_n82	271.005	250.84	294.45	0	t_n82	347.02	313.96	379.31	0
t_n83 THETHYA	228.79	186.07	263.73	L(0.3780,0.1000,0.5000,1e-300)	t_n83 THETHYA	315.936	281.05	351.02	L(0.3780,0.1000,0.5000,1e-300)
t_n84	45.963	23	82.93	0	t_n84	108.33	53.71	174.79	0
t_n85	256.913	237.85	279.57	0	t_n85	330.854	297.89	364.01	0
t_n86	173.353	130.76	213.96	0	t_n86	247.972	186.65	299.89	0
t_n87	108.812	75.22	146.6	0	t_n87	165.207	112.14	218.35	0
t_n88 POECILOSCLERIDA	205.294	199.21	219.24	L(1.9900,0.1000,0.5000,1e-300)	t_n88 POECILOSCLERIDA	212.59	199.41	240.17	L(1.9900,0.1000,0.5000,1e-300)
t_n89	183.281	166.16	199.54	0	t_n89	192.825	176.82	220.16	0
t_n90	167.081	150.1	184.3	0	t_n90	177.225	159.44	204.31	0
t_n91	135.932	117.78	154.46	0	t_n91	141.589	119.98	168.11	0
t_n92	109.163	91.81	127.4	0	t_n92	111.74	90.5	136.84	0
t_n93	93.894	77.43	111.69	0	t_n93	96.071	76.51	119.41	0

t_n94 PHORBAS	70.024	54.79	87.4	L(0.3780,0.1000,0.5000,1e-300)	t_n94 PHORBAS	74.291	57.61	94.9	L(0.3780,0.1000,0.5000,1e-300)
t_n95	49.089	35.03	65.46	0	t_n95	54.232	40.57	71.68	0
t_n96	149.998	126.43	171.73	0	t_n96	157.111	134.25	185.67	0
t_n97	88.803	69.76	109.63	0	t_n97	94.913	72.38	120.65	0
t_n98	71.422	53.88	91.33	0	t_n98	79.37	59.08	102.81	0
t_n99	58.179	41	78.07	0	t_n99	64.237	46.69	85.04	0
t_n100	5.358	3.31	8.22	0	t_n100	6.994	4	11.56	0
t_n101	68.219	48.26	94.21	0	t_n101	143.841	65.09	230.21	0
t_n102	34.13	21.91	50.78	0	t_n102	79.325	32.34	141.67	0
t_n103 ASTROPHORINA	249.583	224.9	277.02	L(1.9900,0.1000,0.5000,1e-300)	t_n103 ASTROPHORINA	290.797	257.3	330.42	L(1.9900,0.1000,0.5000,1e-300)
t_n104	199.961	185.43	219.12	0	t_n104	225.269	203.77	256.23	0
t_n105 GEODIA	166.7	163.1	176.04	L(1.6300,0.1000,0.5000,1e-300)	t_n105 GEODIA	171.826	163.22	193.93	L(1.6300,0.1000,0.5000,1e-300)
t_n106	28.366	17.61	43.29	0	t_n106	54.084	32.7	80.15	0
t_n107	103.253	81.47	126.24	0	t_n107	120.904	101.35	142.95	0
t_n108	84.37	64.9	106.33	0	t_n108	101.644	82.52	122.86	0
t_n109	51.263	37.5	67.97	0	t_n109	65.623	49.6	84	0
t_n110	25.478	16.86	36.71	0	t_n110	35.189	24.76	48.29	0
t_n111	1.788	1.01	2.93	0	t_n111	2.232	1.26	3.74	0
t_n112 SPONGILLIDA	330.911	301.03	366.72	L(2.9800,0.1000,0.5000,1e-300)	t_n112 SPONGILLIDA	433.722	407.5	459.41	L(2.9800,0.1000,0.5000,1e-300)
t_n113	80.812	49.96	123.92	0	t_n113	194.3	106.81	306.48	0
t_n114	87.71	66.94	113.02	0	t_n114	327.482	294.99	359.8	0
t_n115	70.025	49.56	95.19	0	t_n115	309.857	275.13	344.41	0
t_n116	44.267	29.96	62.55	0	t_n116	281.559	247.13	316.9	0
t_n117	5.292	3.73	7.29	0	t_n117	152.267	124.47	183.33	0
t_n118	3.415	2.25	4.93	0	t_n118	87.932	64.23	116.5	0
t_n119	276.571	213.8	336.29	0	t_n119	397.502	338.15	444.03	0
t_n120	198.845	142.11	263.92	0	t_n120	341.744	265.18	404.88	0
t_n121	432.272	390.96	470.4	0	t_n121	500.244	477.51	519.44	0

t_n122	161.414	122.02	209.26	0	t_n122	172.572	69.01	313.43	0
t_n123	78.792	57.86	104.96	0	t_n123	92.724	33.67	187.79	0
t_n124	50.749	33.8	72.88	0	t_n124	62.607	21.75	133.52	0
t_n125	221.766	169.77	281.75	0	t_n125	287.991	177.67	373.04	0
t_n126	118.131	87.8	154.84	0	t_n126	155.17	85.78	227.96	0
t_n127	88.413	61.29	122.31	0	t_n127	122.447	65.41	188.14	0
t_n128 HEXACTINELLIDA	447.732	445.07	454.99	L(4.4500,0.1000,0.5000,1e-300)	T_n128 HEXACTINELLIDA	447.308	445.06	453.36	L(4.4500,0.1000,0.5000,1e-300)
t_n129	433.117	415.1	444.85	0	t_n129	434.484	422.5	443.89	0
t_n130	532.548	480.93	562.42	0	t_n130	566.379	556.69	574.07	0
t_n131	324.35	224.87	421.58	0	t_n131	493.625	420.18	535.63	0
t_n132	271.411	207.53	337.36	0	t_n132	347.621	157.52	460.38	0
t_n133	97.804	63.93	141.51	0	t_n133	141.306	49.76	264.43	0
t_n134	152.246	102.27	208.33	0	t_n134	247.368	96.14	381.18	0
t_n135 EUMETAZOA	572.198	569.91	573.08	B(5.6100,5.7310,0.0250,1e-300)	T_n135 EUMETAZOA	572.138	569.63	573.08	B(5.6100,5.7310,0.0250,1e-300)
t_n136	125.961	72.69	195.46	0	t_n136	178.163	65.34	352.78	0
t_n137	558.524	554.03	562.73	0	t_n137	556.568	553.43	559.54	0
t_n138 BILATERIA	533.43	532.04	537.03	L(5.3200,0.1000,0.1000,1e-300)	T_n138 BILATERIA	533.696	532.05	537.65	L(5.3200,0.1000,0.1000,1e-300)
t_n139 CNIDARIA	533.492	531.85	537.66	L(5.3180,0.1000,0.1000,1e-300)	T_n139 CNIDARIA	536.527	532.1	542.54	L(5.3180,0.1000,0.1000,1e-300)

Table S4 Divergence time estimates obtained with the calibration strategy SDN, for both molecular clocks IR and AR

	**SDN-IR**					**SDN-AR**			
	**Mean div. time**	**2.5% CI**	**97.5% CI**	**Priors**		**Mean div. time**	**2.5% CI**	**97.5% CI**	**Priors**
T_n71 ROOT	610.315	605.92	616.91	B(5.7400,6.0900,1e-300,0.0250)	T_n71 ROOT	591.484	583.49	604.27	B(5.7400,6.0900,1e-300,0.0250)
t_n72	608.138	601.3	615.32	0	t_n72	590.996	582.68	603.79	0
t_n73	581.288	570.3	591.32	0	t_n73	568.471	561.72	577.48	0
t_n74	547.883	536.53	559.17	0	t_n74	543.705	538.18	549.57	0
T_n75 HETEROSCLEROMORPHA	516.905	515.05	521.97	L(5.1500,0.5000,0.5000,1e-300)	T_n75 HETEROSCLEROMORPHA	516.689	515.04	520.81	L(5.1500,0.5000,0.5000,1e-300)
t_n76	447.309	412.61	478.57	0	t_n76	496.554	489.5	503.17	0
t_n77	403.951	368.75	438.04	0	t_n77	474.376	460.89	485.45	0
t_n78	373.428	338.39	408.31	0	t_n78	455.511	436.28	470.99	0
t_n79	338.08	305.31	372.19	0	t_n79	425.887	397.75	448.66	0
t_n80	126.527	59.98	215.67	0	t_n80	223.256	104.76	346.79	0
t_n81	322.887	290.87	356.77	0	t_n81	410.679	379.4	436.63	0
t_n82	296.733	267.19	330.03	0	t_n82	380.923	345.13	412.03	0
T_n83 THETHYA	252.87	199.04	295.9	L(0.3780,0.1000,0.5000,1e-300)	T_n83 THETHYA	347.102	306.93	383.57	L(0.3780,0.1000,0.5000,1e-300)
t_n84	49.452	24.03	91.25	0	t_n84	121.145	58.61	197.27	0
t_n85	280.107	251.82	312.48	0	t_n85	363.014	325.49	396.69	0
t_n86	184.903	136.41	236.04	0	t_n86	271.878	194.47	330.76	0
t_n87	115.62	78.06	158.71	0	t_n87	180.543	117.84	243.11	0
T_n88 POECILOSCLERIDA	213.195	199.74	236.53	L(1.9900,0.1000,0.5000,1e-300)	T_n88 POECILOSCLERIDA	218.2	199.63	254.93	L(1.9900,0.1000,0.5000,1e-300)
t_n89	190.382	169.92	214.39	0	t_n89	197.508	176.93	233.34	0
t_n90	173.422	153	197.6	0	t_n90	181.442	159.5	216.33	0
t_n91	141.298	120.69	163.96	0	t_n91	144.91	119.84	177.89	0

t_n92	113.475	94.44	134.56	0	t_n92	114.52	90.14	144.97	0
t_n93	97.572	79.51	118.07	0	t_n93	98.592	76.25	126.6	0
T_n94 PHORBAS	72.542	56.28	92.04	L(0.3780,0.1000,0.5000,1e-300)	T_n94 PHORBAS	76.423	57.51	100.36	L(0.3780,0.1000,0.5000,1e-300)
t_n95	50.735	35.93	68.78	0	t_n95	55.927	40.67	75.77	0
t_n96	155.672	130	182.52	0	t_n96	160.508	133.89	195.95	0
t_n97	93.13	72.97	115.65	0	t_n97	96.756	71.98	127.16	0
t_n98	74.793	55.94	96.3	0	t_n98	80.945	58.82	108.45	0
t_n99	60.986	42.52	82.7	0	t_n99	65.431	46.41	89.64	0
t_n100	5.695	3.43	8.9	0	t_n100	7.208	4.02	12.31	0
t_n101	73.476	51.51	103.05	0	t_n101	155.794	65.96	256.3	0
t_n102	36.565	23.06	55.3	0	t_n102	86.468	32.82	160.87	0
T_n103 ASTROPHORINA	263.023	231.07	299.37	L(1.9900,0.1000,0.5000,1e-300)	T_n103 ASTROPHORINA	302.114	263.11	349.6	L(1.9900,0.1000,0.5000,1e-300)
t_n104	204.745	187.39	228.83	0	t_n104	229.253	205.57	265.07	0
T_n105 GEODIA	167.654	163.13	179.28	L(1.6300,0.1000,0.5000,1e-300)	t_n105_GEODIA	172.998	163.27	197.64	L(1.6300,0.1000,0.5000,1e-300)
t_n106	30.069	18.53	46.3	0	t_n106	54.279	32.78	80.31	0
t_n107	107.462	85.01	130.04	0	t_n107	121.203	101.4	144.61	0
t_n108	87.836	67.81	110.28	0	t_n108	101.817	82.55	124.18	0
t_n109	53.773	39.25	71.69	0	t_n109	65.656	49.55	84.89	0
t_n110	26.775	17.55	38.97	0	t_n110	35.239	24.75	48.81	0
t_n111	1.888	1.04	3.12	0	t_n111	2.236	1.25	3.79	0
T_n112 SPONGILLIDA	378.697	325.85	424.69	L(2.9800,0.1000,0.5000,1e-300)	T_n112 SPONGILLIDA	475.044	463	485.34	L(2.9800,0.1000,0.5000,1e-300)
t_n113	87.099	52.42	135.13	0	t_n113	218.685	114.69	345.78	0
t_n114	94.741	71.52	123.21	0	t_n114	363.38	333.66	390.36	0
t_n115	75.801	52.9	103.46	0	t_n115	345.117	311.74	375.54	0
t_n116	47.652	31.88	68.43	0	t_n116	314.379	279.92	347.62	0
t_n117	5.56	3.86	7.72	0	t_n117	170.195	139.74	203.04	0
t_n118	3.619	2.35	5.3	0	t_n118	100.024	72.96	132.72	0
t_n119	326.227	246.5	403.84	0	t_n119	450.517	409.36	475.43	0

t_n120	237.875	159.76	321.1	0	t_n120	401.213	338.32	445.46	0
t_n121	482.105	430.44	521.85	0	t_n121	530.528	523.55	536.22	0
t_n122	170.707	126.91	227.91	0	t_n122	162.39	65.48	319.51	0
t_n123	83.447	60.14	112.7	0	t_n123	86.319	32.09	186.12	0
t_n124	53.595	34.93	78.44	0	t_n124	58.064	20.7	130.74	0
t_n125	242.798	180.75	310.25	0	t_n125	319.44	181.23	415.25	0
t_n126	127.479	92.17	168.66	0	t_n126	173.153	87.17	263.53	0
t_n127	95.455	64.34	133.71	0	t_n127	137.371	66.35	218.44	0
T_n128 HEXACTINELLIDA	448.388	445.09	457.3	L(4.4500,0.1000,0.5000,1e-300)	T_n128 HEXACTINELLIDA	447.771	445.07	454.92	L(4.4500,0.1000,0.5000,1e-300)
t_n129	432.639	411.66	446.13	0	t_n129	433.521	419.64	444.51	0
t_n130	534.489	487.64	566.54	0	t_n130	572.171	563.62	582.18	0
t_n131	332.443	228.57	429.15	0	t_n131	507.857	435.36	540.28	0
t_n132	281.402	213.89	351.7	0	t_n132	363.643	163.27	473.24	0
t_n133	102.883	66.28	149.85	0	t_n133	150.452	50.94	280.77	0
t_n134	156.706	105.05	217	0	t_n134	262.718	98.67	399.38	0
T_n135 EUMETAZOA	572.19	569.82	573.07	B(5.6100,5.7310,0.0250,1e-300)	T_n135 EUMETAZOA	572.445	570.7	573.08	B(5.6100,5.7310,0.0250,1e-300)
t_n136	129.759	74.81	201.71	0	t_n136	176.552	65.85	356.75	0
t_n137	558.605	554.02	562.85	0	t_n137	557.065	554.03	560.02	0
T_n138 BILATERIA	533.511	532.04	537.29	L(5.3200,0.1000,0.1000,1e-300)	T_n138 BILATERIA	533.705	532.05	538.14	L(5.3200,0.1000,0.1000,1e-300)
T_n139 CNIDARIA	533.606	531.85	538.05	L(5.3180,0.1000,0.1000,1e-300)	T_n139 CNIDARIA	536.312	532.02	542.72	L(5.3180,0.1000,0.1000,1e-300)

Table S5 MCMC diagnostics for time densities obtained with MCMCtree when fixing the Ctenophora-sister topology, both when the target distribution was set to be the prior (no data, label "CLK") or the posterior (data were used, labels for relaxed-clock models are "AR" for autocorrelated rates and "IR" for independent rates sampled from a log-normal distribution).

SPN strategy

	**Prior-CLK**	**Autocorrelated-AR**	**Independent-IR**
**tail-ESS times (median)**	36,955	2,411	3,689
**tail-ESS times (min)**	3,832	457	653
**tail-ESS times (max)**	59,036	26,768	29,108
**bulk-ESS times (median)**	36,142	1,604	1,843
**bulk-ESS times (min)**	6,172	201	417
**bulk-ESS times (max)**	57,734	26,478	27,075
**Rhat min**	0.999989	1.00001	0.99999
**Rhat max**	1.000565	1.01848	1.00812
**Med. number of samples per chain**	20,001	20,001	20,001
**Min. number of samples per chain**	20,001	20,001	20,001
**Max. number of samples per chain**	20,001	20,001	20,001
**Number of chains that were run**	6	6	6
**Number of chains kept after filters**	6	5	3
**Number of samples used to calculate tail-ESS and bulk-ESS**	120,006	100,005	60,003
**Total number of samples kept *post-hoc* analyses**	120,006	100,005	60,003

Table S6 Divergence time estimates obtained with the Ctenophora-sister topology, for both molecular clocks IR and AR

Ctenophora-sister IR					Ctenophora-sister AR				
**Node**	**Mean_time**	**Mean_qlow**	**Mean_qup**	**Priors**	**Node**	**Mean_time**	**Mean_qlow**	**Mean_qup**	**Priors**
**t_n71_ROOT**	620.879	609.19	637.09	B(5.7400,6.0900,1e-300,0.0250)	**t_n71_ROOT**	610.192	605.77	616.54	B(5.7400,6.0900,1e-300,0.0250)
**t_n72**	619.988	608.34	636.16	0	**t_n72**	608.993	603.83	615.51	0
**t_n73**	601.229	588.65	616.68	0	**t_n73**	596.979	591.5	603.35	0
**t_n74**	576.009	563.87	590.1	0	**t_n74**	574.596	568.21	581.31	0
**t_n75**	544.429	535.06	556.33	0	**t_n75**	547.002	541.51	553.28	0
**t_n76_HETEROSCLEROMORPHA**	516.52	515.04	520.57	L(5.1500,0.5000,0.5000,1e-300)	**t_n76_HETEROSCLEROMORPHA**	517.119	515.06	522.28	L(5.1500,0.5000,0.5000,1e-300)
**t_n77**	449.104	416.58	478.3	0	**t_n77**	495.615	488.05	503.01	0
**t_n78**	406.679	372.97	439.16	0	**t_n78**	472.155	458.34	483.78	0
**t_n79**	376.851	342.66	411.09	0	**t_n79**	452.697	433.62	468.29	0
**t_n80**	341.987	309.44	376.12	0	**t_n80**	422.186	394.62	444.64	0
**t_n81**	129.636	62.91	223.48	0	**t_n81**	218.11	106.17	333.05	0
**t_n82**	327.053	294.97	360.85	0	**t_n82**	406.896	376.4	432.16	0
**t_n83**	300.484	270.09	332.75	0	**t_n83**	376.203	341.63	406.09	0
**t_n84_THETHYA**	258.157	207.84	298.98	L(0.3780,0.1000,0.5000,1e-300)	**t_n84_THETHYA**	342.371	304.05	377.16	L(0.3780,0.1000,0.5000,1e-300)
**t_n85**	49.346	24.49	88.8	0	**t_n85**	115.437	56.79	188.33	0
**t_n86**	283.682	254.39	315.48	0	**t_n86**	358.294	322.57	390.17	0
**t_n87**	187.882	139.84	237.7	0	**t_n87**	267.096	194.58	322.46	0
**t_n88**	118.173	80.37	160.47	0	**t_n88**	176.829	117.05	233.98	0

**t_n89_POECILOSCLERIDA**	215.228	199.95	239.4	L(1.9900,0.1000,0.5000,1e-300)	**t_n89_POECILOSCLERIDA**	218.316	199.67	252.44	L(1.9900,0.1000,0.5000,1e-300)
**t_n90**	192.782	172.05	217.26	0	**t_n90**	197.903	177.83	231.09	0
**t_n91**	175.822	155.07	200.21	0	**t_n91**	181.904	160.81	214.08	0
**t_n92**	143.271	122.45	165.88	0	**t_n92**	145.173	121.7	175.47	0
**t_n93**	115.085	95.91	136.64	0	**t_n93**	114.594	91.92	142.37	0
**t_n94**	98.906	80.86	119.64	0	**t_n94**	98.519	77.64	124.31	0
**t_n95_PHORBAS**	73.749	57.41	93.28	L(0.3780,0.1000,0.5000,1e-300)	**t_n95_PHORBAS**	76.322	58.48	98.55	L(0.3780,0.1000,0.5000,1e-300)
**t_n96**	51.674	36.52	69.55	0	**t_n96**	55.862	41.35	74.33	0
**t_n97**	157.684	132.52	184.61	0	**t_n97**	160.292	134.88	193.16	0
**t_n98**	94.603	74.41	117.32	0	**t_n98**	96.225	72.95	124.43	0
**t_n99**	76.472	57.63	97.96	0	**t_n99**	80.577	59.76	105.99	0
**t_n100**	61.136	42.95	82.31	0	**t_n100**	64.528	46.77	86.85	0
**t_n101**	5.716	3.49	8.83	0	**t_n101**	7.114	4.09	11.82	0
**t_n102**	73.318	52.33	101.05	0	**t_n102**	152.259	68.92	248.11	0
**t_n103**	36.513	23.51	54.02	0	**t_n103**	83.685	34.24	153.37	0
**t_n104_ASTROPHORINA**	264.012	232.26	300.32	L(1.9900,0.1000,0.5000,1e-300)	**t_n104_ASTROPHORINA**	302.173	264.34	348.32	L(1.9900,0.1000,0.5000,1e-300)
**t_n105**	205.431	187.99	229.25	0	**t_n105**	229.994	206.57	265.93	0
**t_n106_GEODIA**	167.654	163.13	179.53	L(1.6300,0.1000,0.5000,1e-300)	**t_n106_GEODIA**	173.19	163.26	198.3	L(1.6300,0.1000,0.5000,1e-300)
**t_n107**	30.08	18.81	45.92	0	**t_n107**	53.775	33.11	79.74	0
**t_n108**	108.039	86.8	130.01	0	**t_n108**	121.249	101.82	145.52	0
**t_n109**	88.218	68.8	109.71	0	**t_n109**	101.65	82.68	124.78	0

**t_n110**	54.137	39.85	71.35	0	**t_n110**	65.43	49.63	85.09	0
**t_n111**	27.155	17.95	39.11	0	**t_n111**	35.162	24.89	48.63	0
**t_n112**	1.968	1.1	3.2	0	**t_n112**	2.312	1.3	3.89	0
**t_n113_SPONGILLIDA**	380.834	329.98	424.9	L(2.9800,0.1000,0.5000,1e-300)	**t_n113_SPONGILLIDA**	473.09	460.82	483.83	L(2.9800,0.1000,0.5000,1e-300)
**t_n114**	88.599	54.29	135.41	0	**t_n114**	210.973	114.8	330.48	0
**t_n115**	93.992	71.45	121.31	0	**t_n115**	356.637	326.87	383.17	0
**t_n116**	74.968	52.89	101.59	0	**t_n116**	337.515	304.27	367.39	0
**t_n117**	47.543	32.05	67.6	0	**t_n117**	305.678	271.65	337.76	0
**t_n118**	5.871	4.11	8.11	0	**t_n118**	164.476	134.67	196.55	0
**t_n119**	3.653	2.41	5.27	0	**t_n119**	92.998	67.77	122.85	0
**t_n120**	328.812	251.32	403.45	0	**t_n120**	444.692	401.28	471.1	0
**t_n121**	239.61	162.6	321.13	0	**t_n121**	391.135	327.05	436.28	0
**t_n122**	477.9	429.58	516.27	0	**t_n122**	532.081	524.92	539.55	0
**t_n123**	172.675	128.88	228.3	0	**t_n123**	170.513	66.66	329.77	0
**t_n124**	83.927	60.98	112.2	0	**t_n124**	90.285	32.5	191.66	0
**t_n125**	54.226	35.72	78.44	0	**t_n125**	61.039	21.1	135.55	0
**t_n126**	242.677	183.31	307.8	0	**t_n126**	321.353	193.67	408.09	0
**t_n127**	128.393	94.56	167.59	0	**t_n127**	174.052	95.56	256.18	0
**t_n128**	96.006	65.82	133.11	0	**t_n128**	137.633	73.03	211.03	0
**t_n129_HEXACTINELLIDA**	448.135	445.08	456.46	L(4.4500,0.1000,0.5000,1e-300)	**t_n129_HEXACTINELLIDA**	447.765	445.07	454.89	L(4.4500,0.1000,0.5000,1e-300)
**t_n130**	432.303	411.7	445.42	0	**t_n130**	432.035	417.44	443.42	0
**t_n131**	531.544	484	561.78	0	**t_n131**	580.247	571.75	588.04	0
**t_n132**	329.003	228.37	422.95	0	**t_n132**	519.716	450.73	551.93	0
**t_n133**	283.457	218.61	350.4	0	**t_n133**	369.55	180.38	476.71	0

**t_n134**	104.466	68.39	150.64	0	**t_n134**	150.764	57.03	274.89	0
**t_n135**	158.103	107.08	216.69	0	**t_n135**	264.037	108.41	396.69	0
**t_n136**	588.718	575.69	603.66	0	**t_n136**	585.899	577.58	594.23	0
**t_n137_BILATERIA**	537.533	532.16	549.19	L(5.3200,0.1000,0.1000,1e-300)	**t_n137_BILATERIA**	546.392	532.92	564.11	L(5.3200,0.1000,0.1000,1e-300)
**t_n138_CNIDARIA**	538.781	532.03	552.24	L(5.3180,0.1000,0.1000,1e-300)	**t_n138_CNIDARIA**	555.98	537.87	572.09	L(5.3180,0.1000,0.1000,1e-300)
**t_n139**	134.069	78.03	206.6	0	**t_n139**	190.263	70.27	381.45	0

Table S7 MCMC diagnostics for time densities obtained with MCMCtree when fixing the tree topology used for BAMM analyses, both when the target distribution was set to be the prior (no data, label "CLK") or the posterior (data were used, labels for relaxed-clock models are "AR" for autocorrelated rates and "IR" for independent rates sampled from a log-normal distribution).

COI Topology used in BAMM

	**Prior-CLK**	**Autocorrelated-AR**	**Independent-IR**
**tail-ESS times (median)**	49408	1198.5	1365.5
**tail-ESS times (min)**	49354	70.0	57.0
**tail-ESS times (max)**	49629	1464.0	2332.0
**bulk-ESS times (median)**	48943	1098.5	851
**bulk-ESS times (min)**	48880	42.0	44
**bulk-ESS times (max)**	49867	1538.0	2393
**Rhat min**	1.00008	0.9963348	0.9969007
**Rhat max**	1.000564	1.475644	3.844148
**Med. number of samples per chain**	20000	20000	20000
**Min. number of samples per chain**	20000	20000	20000
**Max. number of samples per chain**	20000	20000	20000
**Number of chains run**	64	64	64
**Number of chains kept after filters**	64	20	34
**Number of samples used to calculate tail-ESS and bulk-ESS**	100000	12512	11131
**Total number of samples kept *post-hoc* analyses**	100000	12512	11131

Table S8. Statistics obtained with BAMMTools after the BAMM on both divergence timetrees of Silicea with both molecular clocks (AR and IR).

Bamm stats	AR	IR
ESS postburn$N_shifts	2126.471	13860.54
ESS postburn$logLik	1240.406	376.4842
n.shifts	2	2
bulk-ESS times (median)	1098.5	851
bulk-ESS times (min)	42.0	44

Table S9 Spicule types of the species included in the phylogenomic dataset, used to build the morphological matrix for the ASE. Spicule character information was taken from Systema Porifera.

**Class/Order**	***Species***	**Megasclere**	**Microsclere**	**Mineralogy**
**Agelasida**	*Agelas schmidtii*	stylotes	NO	Siliceous
**Agelasida**	*Cymbaxinella damicornis*	oxeas	NO	Siliceous
**Bubarida**	*Cymbastela concentrica*	oxeas	NO	Siliceous
**Bubarida**	*Phakellia robusta*	oxeas	NO	Siliceous
**Bubarida**	*Phakellia ventilabrum*	oxeas	NO	Siliceous
**Chondrillida**	*Chondrilla caribensis*	NO	spherasters, oxyspherasters (microspination on the tips)	Siliceous
**Clionaida**	*Cliona varians*	tylostyles	spirasters	Siliceous
**Clionaida**	*Spheciospongia vesparium*	tylostyles	spirasters	Siliceous
**Dendroceratida**	*Dendrilla antarctica*	NO	NO	Absent
**Dictyoceratida**	*Ircinia fasciculata*	NO	NO	Absent
**Dictyoceratida**	*Spongia officinalis*	NO	NO	Absent
**Dictyoceratida**	*Vaceletia* sp.	NO	NO	Aragonite
**Halisarcida**	*Halisarca caerulea*	NO	NO	Absent
**Halisarcida**	*Halisarca dujardinii*	NO	NO	Absent
**Haplosclerida**	*Amphimedon queenslandica*	oxeas	NO	Siliceous
**Haplosclerida**	*Haliclona penicillata*	oxeas	NO	Siliceous
**Haplosclerida**	*Petrosia ficiformis*	oxeas	NO	Siliceous
**Poecilosclerida**	*Chondrocladia robertballardi*	Mycalostyles	isochaele	Siliceous
**Poecilosclerida**	*Crambe crambe*	Subtylostyles, styles	unguiferate anchorate chelae (28–(38.3)–43m) astrose desmoid spicules rounded knobbed ends, cladome 38–(68.5)–99, rays 10–(35.7)–477 (10.6)–14m	Siliceous
**Poecilosclerida**	*Crella elegans*	Tornotes, oxeote; acanthostyles, acanthoxeas	NO	Siliceous
**Poecilosclerida**	*Iophon unicorne*	Choanosomal oxeote styles, Ectosomal strongyles	Bipocoelles, Anisochelae	Siliceous

**Poecilosclerida**	*Isodictya* sp.	oxeas	isochelae	Siliceous
**Poecilosclerida**	*Kirkpatrickia variolosa*	styles, strongyles	NO	Siliceous
**Poecilosclerida**	*Latrunculia apicalis*	Smooth styles	aciculodiscorhabds	Siliceous
**Poecilosclerida**	*Mycale (Oxymycale) acerata*	oxeas	anisochelae, trichodragmas	Siliceous
**Poecilosclerida**	*Mycale (Mycale) laevis*	oxeas	anisochelae, trichites, anisanchorae	Siliceous
**Poecilosclerida**	*Mycale (Mycale) lingua*	styles/mycalostyles	Anisochelae, sigmas, raphide	Siliceous
**Poecilosclerida**	*Mycale (Carmia) phyllophila*	tylostyle/subtylostyles	Anisochelae, sigmas	Siliceous
**Poecilosclerida**	*Mycale (Mycale) tridens*	Mycalostyles	Chaele	Siliceous
**Poecilosclerida**	*Phorbas areolatus* (antarctica)	acanthostyles, amphioxeas	isochelae	Siliceous
**Poecilosclerida**	*Tedania (Tedania) anhelans*	Styles, tylostyles	onychaetes	Siliceous
**Polymastiida**	*Polymastia invaginata*	Styles, tylostyles	NO	Siliceous
**Scopalinida**	*Scopalina ruetzleri*	styles with oxeote modifications	NO	Siliceous
**Scopalinida**	*Scopalina* sp.	styles	NO	Siliceous
**Spongillida**	*Baikalospongia bacillifera*	slightly curved amphistrongyla, spines near their ends, rarely oxeas (26227m),	NO	Siliceous
**Spongillida**	*Ephydatia fluviatilis*	acanthoxeas, slightly curved or rarely straight oxeas, from smooth to microspined (210–4006–19m).	gemmoscleres Microscleres absent	Siliceous
**Spongillida**	*Eunapius fragilis (emue)*	oxeas	gemmoscleres (spiny amphistrongyla)	Siliceous
**Spongillida**	*Lubomirskia abietina*	curved amphioxea, fusiform, spines near their tips	No mention	Siliceous

**Spongillida**	*Lubomirskia baikalensis*	oxeas, acanthoxeas, slightly curved amphioxea, fusiform, many spines along whole length	Absent	Siliceous
**Spongillida**	*Spongilla lacustris*	oxeas tips gently to sharply pointed, slightly spined if associated to gemmules, acanthoxeas	fusiform oxeas (25–1782–8m) with dense spines regularly distributed along length and microspinosity on spines , asterose shape. Gemmules two types in the same specimen, naked without gemmuloscleres and armoured with gemmuloscleres	Siliceous
**Suberitida**	*Halichondria panicea*	oxeas	NO	Siliceous
**Suberitida**	*Pseudospongosorites suberitoides*	smooth sharp pointed oxeas, occasionally centrotylote and bent, occasionally stylote or strongylote, probably divisible in two overlapping size categories, 176–2952–12m and 125–1852–10m.	No mention	Siliceous
**Suberitida**	*Suberites domuncula*	tylostyles, slightly subterminal (drop-shaped) but mostly well-formed tyle, except for rare annular swellings in the neck region. Small surface tylostyles, 100–3504–8m, larger choanosomal tylostyles 250–4805–8m,	No mention	Siliceous

**Tethyida**	*Tethya wilhelma*	strongyloxeas, anisostrongyles, styles	spherasters, tylasters, strongylasters	Siliceous
**Tetractinellida**	*Craniella infrequens*	anatriaenes, protriaenes, oxeas	NO	Siliceous
**Tetractinellida**	*Geodia barretti*	oxeas, anatriaenes, dichotriaene	sterrasters, oxyaster, strongylaster, microxea	Siliceous
**Tetractinellida**	*Geodia atlantica*	oxeas, dichotriaenes, orthotriaenes	sterrasters, spheroxyasters, oxyasters	Siliceous
**Tetractinellida**	*Geodia hentscheli*	oxeas, dichotriaenes, mesoprotriaenes	sterrasters, oxyaster, strongylaster, microxea	Siliceous
**Tetractinellida**	*Geodia macandrewii*	oxeas, anatriaenes, orthotriaenes, promesotriaene	sterrasters, spheroxyasters, oxyasters	Siliceous
**Tetractinellida**	*Geodia parva*	oxeas, anatriaenes, orthotriaenes, Meso/protriaenes	sterrasters, spherasters, oxyasters	Siliceous
**Tetractinellida**	*Geodia phlegraei*	oxeas, anatriaenes, orthotriaenes, Meso/protriaenes	sterrasters, spherasters, oxyasters	Siliceous
**Tetractinellida**	*Poecillastra compressa*	oxeas, short-shated triaenes	microxeas, spiraster to metaster, plesiaster	Siliceous
**Tetractinellida**	*Stelletta normanii*	oxeas, anatriaenes, dichotriaenes, protriaenes	oxyaster, strongylaster, trichodragma	Siliceous
**Tetractinellida**	*Stryphnus fortis*	oxeas, dichotriaenes, plagiotriaenes, anatriaenes, mesoanatriaenes	oxyasters, sanidasters to amphisanidasters	Siliceous
**Verongiida**	*Aplysina aerophoba*	NO	NO	Absent
**Homoscleromorphida**	*Corticium candelabrum*	Chaltrops	Microchaltrops	Siliceous
**Homoscleromorphida**	*Oscarella pearsei*	NO	NO	Absent
**Lyssacinosida**	*Rossella fibulata*	heterodiactin, pentactin, diactins	oxyhexasters	Siliceous
**Lyssacinosida**	*Sympagella nux*	pinular hexactin, pentactin, diactins	Discohexasters	Siliceous
**Lyssacinosida**	*Vazella pourtalesii*	stauractin, pentactin, diactins	microdiscohexasters, Hemihexasters	Siliceous
**Calcarea**	*Clathrina coriacea*	triactines equiangular and equiradiate		Calcite

**Calcarea**	*Leucetta giribeti*	triactines and tetractines	Calcite
**Calcarea**	*Sycon ciliatum*	triactines	Calcite
**Calcarea**	*Sycon coactum*	triactines and tetractines	Calcite

Table S10 Species collected for the phylogenomic tree, time calibration and ASE analyses.

Species	Code	Order	Class	Accession number
Leucetta giribeti	LGIR	Clathrinida	Calcarea	SRR33975192
Clathrina coriacea	CCOR	Clathrinida	Calcarea	SRR3417192
Sycon ciliatum	SCIL	Leucosolenida	Calcarea	(*131*)
Sycon coactum	SCOA	Leucosolenida	Calcarea	SRS344480
Agelas schmidtii	ASCH	Agelasida	Demospongiae	SRR33939774
Axinella damicornis	CDAM	Agelasida	Demospongiae	SRP224770
Cymbastela concentrica	CCON	Bubarida	Demospongiae	SRP061923
Phakellia robusta	PROB	Bubarida	Demospongiae	SRP271794
Phakellia ventilabrum	PVEN	Bubarida	Demospongiae	SRP268794
Chondrilla caribensis	CCAR	Chondrillida	Demospongiae	ERR13669959
Cliona varians	CVAR	Clionaida	Demospongiae	SRP028615
Spheciospongia vesparium	SVES	Clionaida	Demospongiae	SRR7702355
Dendrilla antarctica	DANT	Dendroceratida	Demospongiae	SRP192036
Ircinia fasciculata	IFAS	Dictyoceratida	Demospongiae	SRR7655554
Vaceletia sp.	VACSP	Dictyoceratida	Demospongiae	SRR4423080
Spongia officinalis	SOFF	Dictyoceratida	Demospongiae	SRP150632
Halisarca caerulea	HCAE	Halisarcida	Demospongiae	SRP098972
Halisarca dujardinii	HDUJ	Halisarcida	Demospongiae	ERR1143554
Amphimedon queenslandica	AQUE	Haplosclerida	Demospongiae	GCA_000090795
Haliclona penicillata	HPEN	Haplosclerida	Demospongiae	SRR33975194
Petrosia ficiformis	PFIC	Haplosclerida	Demospongiae	SRR504687
Crella elegans	CELE	Poecilosclerida	Demospongiae	SRR648683
Iophon unicorne	IUNI	Poecilosclerida	Demospongiae	SRR33975193
Kirkpatrickia variolosa	KVAR	Poecilosclerida	Demospongiae	SRR1916957
Latrunculia apicalis	LAPI	Poecilosclerida	Demospongiae	SRR1915755
Mycale laevis	MLAE	Poecilosclerida	Demospongiae	SRP252526
Mycale tridens	MTRI	Poecilosclerida	Demospongiae	SRS6316513
Mycale acerata	MACE	Poecilosclerida	Demospongiae	SRS6305371
Mycale lingua	MLIN	Poecilosclerida	Demospongiae	SRR33975190
Mycale phyllophyla	MPHY	Poecilosclerida	Demospongiae	SRP051131
Isodyctia sp.	ISOSP	Poecilosclerida	Demospongiae	SRP120971
Crambe crambe	CCRA	Poecilosclerida	Demospongiae	SRR33975191
Phorbas areolatus	PARE	Poecilosclerida	Demospongiae	SRS5425827
Chondrocladia robertballardi	CROB	Poecilosclerida	Demospongiae	SRR33975189
Tedania anhelans	TANH	Poecilosclerida	Demospongiae	SRP061923
Polymastia invaginata	PINV	Polymastiida	Demospongiae	SRR33975188
Scopalina ruetzleri	SRUE	Scopalinida	Demospongiae	SRR33975186
Scopalina sp.	SCOPSP	Scopalinida	Demospongiae	SRP061923
Spongilla lacustris	SLAC	Spongillida	Demospongiae	SRS557419
Ephydatia muelleri	EMUE	Spongillida	Demospongiae	SRP371964

Eunapius fragilis	EFRA	Spongillida	Demospongiae	https://era.library.ualberta.ca/items/6139a88f-895d-44a7-bd0d-e22d455d2785
Lubomirskia baikalensis	LBAI	Spongillida	Demospongiae	SRR6667231
Lubomirskia abietina	LABI	Spongillida	Demospongiae	PRJNA431612
Baikalospongia bacillifera	BBAC	Spongillida	Demospongiae	PRJNA431612
Pseudospongosorites suberitoides	PSUB	Suberitida	Demospongiae	SRP037540
Prosuberites sp.	PROSP	Suberitida	Demospongiae	SRR33975187
Halichondria panicea	HPAN	Suberitida	Demospongiae	SRP100065
Geodia barretti	GBAR	Tetractinellida	Demospongiae	SRP246203
Geodia atlantica	GATL	Tetractinellida	Demospongiae	SRP246203
Geodia macandrewi	GMAC	Tetractinellida	Demospongiae	SRP246203
Geodia parva	GPAR	Tetractinellida	Demospongiae	SRP246203
Geodia phlegraei	GPHL	Tetractinellida	Demospongiae	SRP246203
Geodia hentscheli	GHEN	Tetractinellida	Demospongiae	SRP246203
Poecillastra compressa	PCOM	Tetractinellida	Demospongiae	SRP224770
Stelletta normani	SNOR	Tetractinellida	Demospongiae	SRR33975185
Stryphnus fortis	SFOR	Tetractinellida	Demospongiae	SRP224770
Craniella infrequens	CINF	Tetractinellida	Demospongiae	SRR33975184
Tethya wilhelma	TWIL	Tethyida	Demospongiae	SRR4255675
Aplysina aerophoba	AAER	Verongiida	Demospongiae	ERR2220853
Rossella fibulata	RFIB	Lyssacinosida	Hexactinellida	SRR1915835
Sympagella nux	SNUX	Lyssacinosida	Hexactinellida	SRR1916581
Vazella pourtalesii	VPOU	Lyssacinosida	Hexactinellida	SRS6494416
Corticium candelabrum	CCAN	Homosclerophorida	Homoscleromorpha	SRP012620
Oscarella pearsei	OPEA	Homosclerophorida	Homoscleromorpha	SRR1042040
Pleurobrachia bachei	PBAC	Outgroup	Ctenophora	GCA_000695325.1
Bolinopsis ashley	BASH	Outgroup	Ctenophora	SRR5892570
Nematostella vectensis	NVEC	Outgroup	Cnidaria	ASM20922v1
Aurelia aurita	AAUR	Outgroup	Cnidaria	SRR10240021
Lottia gigantea	LGIG	Outgroup	Mollusca	GCF_000327385.1
Homo sapiens	HSAP	Outgroup	Chordata	GCA_000306695.2

Table S11 Convergence values for the two bayesian chains estimated with Tracecomp (Burnin = 1500), as implemented in Phylobayes

Name	Effective sample size	Real_diff
**Log-likelihood**	61	0.15233
**Tree length**	709	0.0709294
**Alpha parameter of gamma distribution**	595	0.0110885
**Number of modes**	169	0.224488
**State of entropy**	316	0.099967
**State alpha**	1047	0.138845

Table S12 ST calibrations applied to the COI topology used for the diversification rates analyses in BAMM and MEDUSA

Name of clades corresponding to nodes	Node	Prior
**Silicea**	808	ST(5.8300,0.0590,0.1120,109.1240)
**Demospongiae**	809	ST(5.4510,0.0700,0.9220,209.2980)
**Haplosclerida + + Heteroscleromorpha**	810	ST(5.1500,0.0190,394.1320,3.5100)
**After split with Haplosclerida**	811	ST(4.5960,0.2010,-1.1000,404.5290)
**After split with Scopalinida +Spongillida**	812	ST(4.0910,0.1770,-0.3850,246.6070)
**After split with Bubarida**	813	ST(3.6390,0.1960,0.7740,151.2750)
**After split with Agelasida**	815	ST(3.2620,0.2050,1.1200,431.6690)
**Poecilosclerida**	928	ST(2.6670,0.2050,1.5490,477.3950)
**Bubarida**	1143	ST(0.6040,0.1810,2.1930,136.9570)
**Tetractinellida**	1179	ST(2.4770,0.2300,1.4590,701.2120)
**Spongillida + Scopalinida**	1353	ST(4.0020,0.3260,-1.4770,1215.9540)
**Haplosclerida**	1392	ST(3.5230,0.4820,-0.9020,983.7760)
**Hexactinellida**	1563	ST(4.4500,0.0330,1588.4600,3.5420)

Table S13 Sample frequencies used for the BAMM and MEDUSA analysis

Clade	Species Sampled	Num. species accepted	Sampling frquency
**Silicea**	807	8649	0.09330558
**Hexactinellida**	52	701	0.07417974
**Agelasida**	26	73	0.35616438
**Tetractinellida**	175	1175	0.14893617
**Haplosclerida**	66	1147	0.05754141
**Poecilosclerida**	165	2448	0.06740196
**Polymastiida**	33	132	0.25
**Axinellida**	25	534	0.04681648
**Bubarida**	12	153	0.07843137
**Spongillida**	33	283	0.11660777
**Tethyida**	27	212	0.12735849
**Biemnida**	10	106	0.09433962
**Sphaerocladina**	4	5	0.8
**Dictyoceratida**	51	588	0.08673469
**Dendorceratida**	17	70	0.24285714
**Verongiida**	31	95	0.32631579
**Scopalinida**	3	39	0.07692308
**Suberitida**	40	519	0.07707129
**Chondrosida**	2	11	0.18181818
**Chondrillida**	6	42	0.14285714
**Clionaida**	25	213	0.11737089
**Desmacellida**	3	37	0.08108108

Data S1. (separate file)

Table with all the ASE results for each topology analysed.

REFERENCES

1. J. R. Pawlik, S. E. McMurray, The emerging ecological and biogeochemical importance of sponges on coral reefs. *Ann. Rev. Mar. Sci.* **12**, 315–337 (2020).

2. C. W. Dunn, A. Hejnol, D. Q. Matus, K. Pang, W. E. Browne, S. A. Smith, E. Seaver, G. W. Rouse, M. Obst, G. D. Edgecombe, M. V. Sørensen, S. H. D. Haddock, A. Schmidt-Rhaesa, A. Okusu, R. M. Kristensen, W. C. Wheeler, M. Q. Martindale, G. Giribet, Broad phylogenomic sampling improves resolution of the animal tree of life. *Nature* **452**, 745–749 (2008).

3. D. Pisani, W. Pett, M. Dohrmann, R. Feuda, O. Rota-Stabelli, H. Philippe, N. Lartillot, G. Wörheide, Genomic data do not support comb jellies as the sister group to all other animals. *Proc. Natl. Acad. Sci. U.S.A.* **112**, 15402–15407 (2015).

4. R. Feuda, M. Dohrmann, W. Pett, H. Philippe, O. Rota-Stabelli, N. Lartillot, G. Wörheide, D. Pisani, Improved modeling of compositional heterogeneity supports sponges as sister to all other animals. *Curr. Biol.* **27**, 3864–3870.e4 (2017).

5. D. T. Schultz, S. H. D. Haddock, J. V. Bredeson, R. E. Green, O. Simakov, D. S. Rokhsar, Ancient gene linkages support ctenophores as sister to other animals. *Nature* **618**, 110–117 (2023).

6. R. R. Copley, Sponges, ctenophores and the statistical significance of syntenies. *Mol. Biol. and Evol.* , msaf321 (2025).

7. H. Philippe, R. Derelle, P. Lopez, K. Pick, C. Borchiellini, N. Boury-Esnault, J. Vacelet, E. Renard, E. Houliston, E. Quéinnec, C. Da Silva, P. Wincker, H. Le Guyader, S. Leys, D. J. Jackson, F. Schreiber, D. Erpenbeck, B. Morgenstern, G. Wörheide, M. Manuel, Phylogenomics revives traditional views on deep animal relationships. *Curr. Biol.* **19**, 706–712 (2009).

8. J. L. Steenwyk, N. King, Integrative phylogenomics positions sponges at the root of the animal tree. *Science* **390**, 751–756 (2025).

9. D. A. Gold, J. Grabenstatter, A. De Mendoza, A. Riesgo, I. Ruiz-Trillo, R. E. Summons, Sterol and genomic analyses validate the sponge biomarker hypothesis. *Proc. Natl. Acad. Sci. U.S.A.* **113**, 2684–2689 (2016).

10. E. A. Sperling, J. M. Robinson, D. Pisani, K. J. Peterson, Where's the glass? Biomarkers, molecular clocks, and microRNAs suggest a 200-Myr missing Precambrian fossil record of siliceous sponge spicules: Sponge biomarkers, molecular clocks and microRNAs. *Geobiology* **8**, 24–36 (2010).

11. J. A. Zumberge, G. D. Love, P. Cárdenas, E. A. Sperling, S. Gunasekera, M. Rohrssen, E. Grosjean, J. P. Grotzinger, R. E. Summons, Demosponge steroid biomarker 26-methylstigmastane provides evidence for Neoproterozoic animals. *Nat. Ecol. Evol.* **2**, 1709–1714 (2018).

12. D. H. Erwin, M. Laflamme, S. M. Tweedt, E. A. Sperling, D. Pisani, K. J. Peterson, The Cambrian conundrum: Early divergence and later ecological success in the early history of animals. *Science* **334**, 1091–1097 (2011).

13. M. Dohrmann, G. Wörheide, Dating early animal evolution using phylogenomic data. *Sci. Rep.* **7**, 3599 (2017).

14. J. P. Botting, L. A. Muir, Early sponge evolution: A review and phylogenetic framework. *Palaeoworld* **27**, 1–29 (2018).

15. J. B. Antcliffe, R. H. T. Callow, M. D. Brasier, Giving the early fossil record of sponges a squeeze. *Biol. Rev.* **89**, 972–1004 (2014).

16. E. C. Turner, Possible poriferan body fossils in early Neoproterozoic microbial reefs. *Nature* **596**, 87–91 (2021).

17. F. Neuweiler, S. Kershaw, F. Boulvain, M. Matysik, C. Sendino, M. McMenamin, A. Munnecke, Keratose sponges in ancient carbonates – A problem of interpretation. *Sedimentology* **70**, 927–968 (2023).

18. I. Bobrovskiy, J. M. Hope, B. J. Nettersheim, J. K. Volkman, C. Hallmann, J. J. Brocks, Algal origin of sponge sterane biomarkers negates the oldest evidence for animals in the rock record. *Nat. Ecol. Evol.* **5**, 165–168 (2021).

19. A. C. Maloof, C. V. Rose, R. Beach, B. M. Samuels, C. C. Calmet, D. H. Erwin, G. R. Poirier, N. Yao, F. J. Simons, Possible animal-body fossils in pre-Marinoan limestones from South Australia. *Nat. Geosci.* **3**, 653–659 (2010).

20. Z. Yin, M. Zhu, E. H. Davidson, D. J. Bottjer, F. Zhao, P. Tafforeau, Sponge grade body fossil with cellular resolution dating 60 Myr before the Cambrian. *Proc. Natl. Acad. Sci. U.S.A.* **112**, E1453–E1460 (2015).

21. J. A. Cunningham, A. G. Liu, S. Bengtson, P. C. J. Donoghue, The origin of animals: Can molecular clocks and the fossil record be reconciled? *Bioessays* **39**, 1–12 (2017).

22. E. Carlisle, Z. Yin, D. Pisani, P. C. J. Donoghue, Ediacaran origin and Ediacaran-Cambrian diversification of Metazoa. *Sci. Adv.* **10**, eadp7161 (2024).

23. M. Brasier, O. Green, G. Shields, Ediacarian sponge spicule clusters from southwestern Mongolia and the origins of the Cambrian fauna. *Geology* **25**, 303–306 (1997).

24. Y. Zhang, X. Yuan, L. Yin, Interpreting Late Precambrian microfossils. *Science* **282**, 1783 (1998).

25. S. Chang, Q. Feng, S. Clausen, L. Zhang, Sponge spicules from the lower Cambrian in the Yanjiahe Formation, South China: The earliest biomineralizing sponge record. *Palaeogeogr. Palaeoclimatol. Palaeoecol.* **474**, 36–44 (2017).

26. Q. Tang, B. Wan, X. Yuan, A. D. Muscente, S. Xiao, Spiculogenesis and biomineralization in early sponge animals. *Nat. Commun.* **10**, 3348 (2019).

27. W. R. Francis, M. Eitel, S. Vargas, C. A. Garcia-Escudero, N. Conci, F. Deister, J. L. Mah, N. Guiglielmoni, S. Krebs, H. Blum, S. P. Leys, G. Wörheide, The genome of the reef-building glass sponge *Aphrocallistes vastus* provides insights into silica biomineralization. *R. Soc. Open Sci.* **10**, 230423 (2023).

28. W. E. Müller, A. Krasko, G. Le Pennec, R. Steffen, M. Wiens, M. S. A. Ammar, I. M. Müller, H. C. Schröder, Molecular mechanism of spicule formation in the demosponge suberites domuncula: Silicatein-collagen-myotrophin. *Prog. Mol. Subcell. Biol.* **33**, 195–221 (2003).

29. A. Riesgo, M. Maldonado, S. López-Legentil, G. Giribet, A proposal for the evolution of cathepsin and silicatein in sponges. *J. Mol. Evol.* **80**, 278–291 (2015).

30. K. Shimizu, M. Nishi, Y. Sakate, H. Kawanami, T. Bito, J. Arima, L. Leria, M. Maldonado, Silica-associated proteins from hexactinellid sponges support an alternative evolutionary scenario for biomineralization in Porifera. *Nat. Commun.* **15**, 181 (2024).

31. O. Voigt, M. V. Wilde, T. Fröhlich, B. Fradusco, S. Vargas, G. Wörheide, Genetic parallels in biomineralization of the calcareous sponge Sycon ciliatum and stony corals. *eLife* **14**, RP106239 (2025).

32. O. Voigt, M. Adamska, M. Adamski, A. Kittelmann, L. Wencker, G. Wörheide, Spicule formation in calcareous sponges: Coordinated expression of biomineralization genes and spicule-type specific genes. *Sci. Rep.* **7**, 45658 (2017).

33. G. Wörheide, A hypercalcified sponge with soft relatives: Vaceletia is a keratose demosponge. *Mol. Phylogenet. Evol.* **47**, 433–438 (2008).

34. J. Jeon, M. Simonet Roda, Z.-Y. Chen, C. Luo, S. Kershaw, D. Kim, J.-Y. Ma, J.-H. Lee, Y.-D. Zhang, Phosphatic stromatoporoid sponges formed reefs ~480 Mya. *Proc. Natl. Acad. Sci. U.S.A.* **122**, e2426105122 (2025).

35. P. U. P. A. Gilbert, K. D. Bergmann, N. Boekelheide, S. Tambutté, T. Mass, F. Marin, J. F. Adkins, J. Erez, B. Gilbert, V. Knutson, M. Cantine, J. O. Hernández, A. H. Knoll, Biomineralization: Integrating mechanism and evolutionary history. *Sci. Adv.* **8**, eabl9653 (2022).

36. D. J. Jackson, L. Macis, J. Reitner, B. M. Degnan, G. Wörheide, Sponge paleogenomics reveals an ancient role for carbonic anhydrase in skeletogenesis. *Science* **316**, 1893–1895 (2007).

37. O. Voigt, B. Fradusco, C. Gut, C. Kevrekidis, S. Vargas, G. Wörheide, Carbonic anhydrases: An ancient tool in calcareous sponge biomineralization. *Front. Genet.* **12**, 624533 (2021).

38. O. Voigt, M. Adamski, K. Sluzek, M. Adamska, Calcareous sponge genomes reveal complex evolution of α-carbonic anhydrases and two key biomineralization enzymes. *BMC Evol. Biol.* **14**, 230 (2014).

39. J. Germer, K. Mann, G. Wörheide, D. J. Jackson, The skeleton forming proteome of an early branching metazoan: A molecular survey of the biomineralization components employed by the coralline sponge Vaceletia Sp. *PLOS ONE* **10**, e0140100 (2015).

40. S. M. Rowland, Archaeocyaths—A history of phylogenetic interpretation. *J. Paleo.* **75**, 1065–1078 (2001).

41. J. P. Botting, N. J. Butterfield, Reconstructing early sponge relationships by using the Burgess Shale fossil Eiffelia globosa, Walcott. *Proc. Natl. Acad. Sci. U.S.A.* **102**, 1554–1559 (2005).

42. X. Wang, A. G. Liu, Z. Chen, C. Wu, Y. Liu, B. Wan, K. Pang, C. Zhou, X. Yuan, S. Xiao, A late-Ediacaran crown-group sponge animal. *Nature* **630**, 905–911 (2024).

43. J. P. Botting, D. Janussen, M. Dohrmann, L. A. Muir, Y. Zhang, J. Ma, Advanced crown-group Rossellidae (Porifera: Hexactinellida) resembling extant taxa from the Hirnantian (Late Ordovician) Anji Biota. *Pap. Palaeontol.* **11**, e70000 (2025).

44. B. Runnegar, J. G. Gehling, S. Jensen, M. R. Saltzman, Ediacaran paleobiology and biostratigraphy of the Nama Group, Namibia, with emphasis on the erniettomorphs, tubular and trace fossils, and a new sponge, *Arimasia* germsi n. gen. n. sp. *J. Paleontol.* **98**, 1–59 (2024).

45. F. Wei, Y. Zhao, A. Chen, X. Hou, P. Cong, New vauxiid sponges from the Chengjiang Biota and their evolutionary significance. *J. Geol. Soc. London* **178**, doi.org/10.1144/jgs2020-162 (2021).

46. S. Tarasov, Integration of anatomy ontologies and evo-devo using structured markov models suggests a new framework for modeling discrete phenotypic traits. *Syst. Biol.* **68**, 698–716 (2019).

47. S. Tarasov, New phylogenetic Markov models for inapplicable morphological characters. *Syst. Biol.* **72**, 681–693 (2023).

48. N. Shubin, C. Tabin, S. Carroll, Deep homology and the origins of evolutionary novelty. *Nature* **457**, 818–823 (2009).

49. S. Tarasov, The invariant nature of a morphological character and character state: Insights from gene regulatory networks. *Syst. Biol.* **69**, 392–400 (2020).

50. D. M. Emms, S. Kelly, OrthoFinder2: Fast and accurate phylogenomic orthology analysis from gene sequences. *BioRxiv* 10.1101/466201 (2018).

51. B. Q. Minh, H. A. Schmidt, O. Chernomor, D. Schrempf, M. D. Woodhams, A. von Haeseler, R. Lanfear, IQ-TREE 2: New models and efficient methods for phylogenetic inference in the genomic era. *Mol. Biol. Evol.* **37**, 1530–1534 (2020).

52. N. Lartillot, H. Philippe, A Bayesian mixture model for across-site heterogeneities in the amino-acid replacement process. *Mol. Biol. Evol.* **21**, 1095–1109 (2004).

53. N. Lartillot, N. Rodrigue, D. Stubbs, J. Richer, PhyloBayes MPI: Phylogenetic reconstruction with infinite mixtures of profiles in a parallel environment. *Syst. Biol.* **62**, 611–615 (2013).

54. S. Mirarab, R. Reaz, M. S. Bayzid, T. Zimmermann, M. S. Swenson, T. Warnow, ASTRAL: Genome-scale coalescent-based species tree estimation. *Bioinformatics* **30**, i541–i548 (2014).

55. Z. Yang, PAML 4: Phylogenetic analysis by maximum likelihood. *Mol. Biol. Evol.* **24**, 1586–1591 (2007).

56. F. S. Dunn, A. G. Liu, D. V. Grazhdankin, P. Vixseboxse, J. Flannery-Sutherland, E. Green, S. Harris, P. R. Wilby, P. C. J. Donoghue, The developmental biology of Charnia

and the eumetazoan affinity of the Ediacaran rangeomorphs. *Sci. Adv.* **7**, eabe0291 (2021).

57. C. Yang, Y. Li, D. Selby, B. Wan, C. Guan, C. Zhou, X.-H. Li, Implications for Ediacaran biological evolution from the ca. 602 Ma Lantian biota in China. *Geology* **50**, 562–566 (2022).

58. P. R. Wilby, J. N. Carney, M. P. Howe, A rich Ediacaran assemblage from eastern Avalonia: Evidence of early widespread diversity in the deep ocean. *Geology* **39**, 655–658 (2011).

59. S. R. Noble, D. J. Condon, J. N. Carney, P. R. Wilby, T. C. Pharaoh, T. D. Ford, U-Pb geochronology and global context of the Charnian Supergroup, UK: Constraints on the age of key Ediacaran fossil assemblages. *Geol. Soc. Am. Bull.* **127**, 250–265 (2015).

60. J. L. Thorne, H. Kishino, I. S. Painter, Estimating the rate of evolution of the rate of molecular evolution. *Mol. Biol. Evol.* **15**, 1647–1657 (1998).

61. B. Rannala, Z. Yang, Inferring speciation times under an episodic molecular clock. *Syst. Biol.* **56**, 453–466 (2007).

62. C. D. Walcott, Middle Cambrian Spongiae. *Smithsonian Misc*. Collect. (1917).

63. J. W. Salter, On some new fossils from the lingula-flags of Wales. *Quart. J. Geol. Soc. London* **20**, 233–241 (1864).

64. R. M. Finks, R. L. Kaesler, J. K. Rigby, "Paleozoic demospongea: Morphology Morphology and phylogeny," in *Treatise on Invertebrate Paleontology, Pt. E, Porifera (revised)* (Geological Society of America, 2003), vol. 2, pp. 63–80.

65. C. Luo, F. Zhao, H. Zeng, The first report of a vauxiid sponge from the Cambrian Chengjiang Biota. *J. Paleo.* **94**, 28–33 (2020).

66. X.-L. Yang, Y.-L. Zhao, L. E. Babcock, J. Peng, Siliceous spicules in a vauxiid sponge (Demospongia) from the Kaili Biota(Cambrian Stage 5), Guizhou, South China. *Sci. Rep.* **7**, 42945 (2017).

67. K. A. Kolesnikov, J. P. Botting, A. Y. Ivantsov, A. Y. Zhuravlev, New early Cambrian sponges of the Siberian platform and the origins of spiculate crown-group demosponges. *Pap. Palaeontol.* **4**, e1582 (2024).

68. M. B. DeBiasse, A. Buckenmeyer, J. Macrander, L. S. Babonis, B. Bentlage, P. Cartwright, C. Prada, A. M. Reitzel, S. N. Stampar, A. G. Collins, A cnidarian phylogenomic tree fitted with hundreds of 18S leaves. *Bull. Soc. Syst. Biol.* **3**, (2024).

69. M. E. Alfaro, F. Santini, C. Brock, H. Alamillo, A. Dornburg, D. L. Rabosky, G. Carnevale, L. J. Harmon, Nine exceptional radiations plus high turnover explain species diversity in jawed vertebrates. *Proc. Natl. Acad. Sci. U.S.A.* **106**, 13410–13414 (2009).

70. D. V. Lavrov, M. C. Diaz, M. Maldonado, C. C. Morrow, T. Perez, S. A. Pomponi, R. W. Thacker, Phylomitogenomics bolsters the high-level classification of Demospongiae (phylum Porifera). *PLOS ONE* **18**, e0287281 (2023).

71. S. Santini, Q. Schenkelaars, C. Jourda, M. Duchesne, H. Belahbib, C. Rocher, M. Selva, A. Riesgo, M. Vervoort, S. P. Leys, L. Kodjabachian, A. L. Bivic, C. Borchiellini, J.-M. Claverie, E. Renard, The compact genome of the sponge Oopsacas minuta (Hexactinellida) is lacking key metazoan core genes. *BMC Biol.* **21**, 139 (2023).

72. W. E. G. Müller, U. Schloßmacher, C. Eckert, A. Krasko, A. Boreiko, H. Ushijima, S. E. Wolf, W. Tremel, I. M. Müller, H. C. Schröder, Analysis of the axial filament in spicules of the demosponge Geodia cydonium: Different silicatein composition in microscleres (asters) and megascleres (oxeas and triaenes). *Eur. J. Cell Biol.* **86**, 473–487 (2007).

73. K. Shimizu, J. Cha, G. D. Stucky, D. E. Morse, Silicatein α: Cathepsin L-like protein in sponge biosilica. *Proc. Natl. Acad. Sci. U.S.A.* **95**, 6234–6238 (1998).

74. K. Shimizu, T. Amano, M. R. Bari, J. C. Weaver, J. Arima, N. Mori, Glassin, a histidine-rich protein from the siliceous skeletal system of the marine sponge Euplectella, directs silica polycondensation. *Proc. Natl. Acad. Sci. U.S.A.* **112**, 11449–11454 (2015).

75. M. Maldonado, A. Riesgo, Intra-epithelial spicules in a homosclerophorid sponge. *Cell Tissue Res.* **328**, 639–650 (2007).

76. N. de Voogd, B. Alvarez, N. Boury-Esnault, P. Cárdenas, M.-C. Díaz, M. Dohrmann, R. Downey, C. Goodwin, E. Hajdu, J. Hooper, M. Kelly, M. Klautau, S.-C. Lim, R. Manconi, C. Morrow, U. Pinheiro, A. Pisera, P. Ríos, K. Rützler, C. Schönberg, T. Turner, J. Vacelet, R. van Soest, J. Xavier, World Porifera Database. Accessed at https://www.marinespecies.org/porifera, VLIZ (2023); https://doi.org/10.14284/359.

77. J. Vacelet, N. Boury-Esnault, Carnivorous sponges. *Nature* **373**, 333–335 (1995).

78. M.-J. Uriz, X. Turon, M. A. Becerro, G. Agell, Siliceous spicules and skeleton frameworks in sponges: Origin, diversity, ultrastructural patterns, and biological functions. *Micros. Res. Tech.* **62**, 279–299 (2003).

79. M. Łukowiak, R. Van Soest, M. Klautau, T. Pérez, A. Pisera, K. Tabachnick, The terminology of sponge spicules. *J. Morphol.* **283**, 1517–1545 (2022).

80. C. Morrow, P. Cárdenas, Proposal for a revised classification of the Demospongiae (Porifera). *Front. Zool.* **12**, 7 (2015).

81. M. dos Reis, Y. Thawornwattana, K. Angelis, M. J. Telford, P. C. J. Donoghue, Z. Yang, Uncertainty in the timing of origin of animals and the limits of precision in molecular timescales. *Curr. Biol.* **25**, 2939–2950 (2015).

82. J. B. Antcliffe, Questioning the evidence of organic compounds called sponge biomarkers. *Palaeontology* **56**, 917–925 (2013).

83. D. A. Gold, S. S. O'Reilly, G. Luo, D. E. G. Briggs, R. E. Summons, Prospects for sterane preservation in sponge fossils from museum collections and the utility of sponge biomarkers for molecular clocks. *Bull. Peabody Mus. Nat. Hist.* **57**, 181–189 (2016).

84. B. J. Nettersheim, J. J. Brocks, A. Schwelm, J. M. Hope, F. Not, M. Lomas, C. Schmidt, R. Schiebel, E. C. M. Nowack, P. De Deckker, J. Pawlowski, S. S. Bowser, I. Bobrovskiy, K.

Zonneveld, M. Kucera, M. Stuhr, C. Hallmann, Putative sponge biomarkers in unicellular Rhizaria question an early rise of animals. *Nat. Ecol. Evol.* **3**, 577–581 (2019).

85. L. Shawar, G. D. Love, B. T. Uveges, J. A. Zumberge, P. Cárdenas, J.-L. Giner, R. E. Summons, Chemical characterization of C_{31} sterols from sponges and Neoproterozoic fossil sterane counterparts. *Proc. Natl. Acad. Sci. U.S.A.* **122**, e2503009122 (2025).

86. Z. Wang, X. Xie, Z. Wen, Formation conditions of Ediacaran–Cambrian cherts in South China: Implications for marine redox conditions and paleoecology. *Precambrian Res.* **383**, 106867 (2022).

87. A. Muscente, F. M. Michel, J. G. Dale, S. Xiao, Assessing the veracity of Precambrian 'sponge' fossils using in situ nanoscale analytical techniques. *Precambrian Res.* **263**, 142–156 (2015).

88. M. J. Donoghue, J. A. Doyle, J. Gauthier, A. G. Kluge, T. Rowe, The importance of fossils in phylogeny reconstruction. *Annu. Rev. Ecol. Syst.* **20**, 431–460 (1989).

89. C. Patterson, Significance of fossils in determining evolutionary relationships. *Annu. Rev. Ecol. Syst.* **12**, 195–223 (1981).

90. K. J. Peterson, R. E. Summons, P. C. Donoghue, Molecular palaeobiology. *Palaeontology* **50**, 775–809 (2007).

91. L.-T. Nguyen, H. A. Schmidt, A. von Haeseler, B. Q. Minh, IQ-TREE: A fast and effective stochastic algorithm for estimating maximum-likelihood phylogenies. *Mol. Biol. Evol.* **32**, 268–274 (2015).

92. J. P. Botting, P. Cárdenas, J. S. Peel, A crown-group demosponge from the early Cambrian Sirius Passet Biota, North Greenland. *Palaeontology* **58**, 35–43 (2015).

93. P. Cárdenas, Surface microornamentation of demosponge sterraster spicules, phylogenetic and paleontological implications. *Front. Mar. Sci.* **7**, doi.org/10.3389/fmars.2020.613610 (2020).

94. F. Ronquist, M. Teslenko, P. van der Mark, D. L. Ayres, A. Darling, S. Höhna, B. Larget, L. Liu, M. A. Suchard, J. P. Huelsenbeck, MRBAYES 3.2: Efficient Bayesian phylogenetic inference and model selection across a large model space. *Syst. Biol.* **61**, 539–542 (2012).

95. D. S. Porto, J. Uyeda, I. Mikó, S. Tarasov, Ontophylo: Reconstructing the evolutionary dynamics of phenomes using new ontology-informed phylogenetic methods. *Methods Ecol. Evol.* **15**, 290–300 (2024).

96. D. L. Rabosky, Automatic detection of key innovations, rate shifts, and diversity-dependence on phylogenetic trees. *PLOS ONE* **9**, e89543 (2014).

97. S. Andrews, FastQC: A quality control tool for high throughput sequence data, (2010). Retrieved from: https://www.bioinformatics.babraham.ac.uk/projects/fastqc/.

98. A. M. Bolger, M. Lohse, B. Usadel, Trimmomatic: A flexible trimmer for Illumina sequence data. *Bioinformatics* **30**, 2114–2120 (2014).

99. M. G. Grabherr, B. J. Haas, M. Yassour, J. Z. Levin, D. A. Thompson, I. Amit, X. Adiconis, L. Fan, R. Raychowdhury, Q. Zeng, Z. Chen, E. Mauceli, N. Hacohen, A. Gnirke, N. Rhind, F. di Palma, B. W. Birren, C. Nusbaum, K. Lindblad-Toh, N. Friedman, A. Regev, Full-length transcriptome assembly from RNA-Seq data without a reference genome. *Nat. Biotechnol.* **29**, 644–652 (2011).

100. L. Fu, B. Niu, Z. Zhu, S. Wu, W. Li, CD-HIT: Accelerated for clustering the next-generation sequencing data. *Bioinformatics* **28**, 3150–3152 (2012).

101. K. M. Kocot, M. R. Citarella, L. L. Moroz, K. M. Halanych, PhyloTreePruner: A phylogenetic tree-based approach for selection of orthologous sequences for phylogenomics. *Evol. Bioinform. Online* **9**, 429–435 (2013).

102. S. Whelan, I. Irisarri, F. Burki, PREQUAL: Detecting non-homologous characters in sets of unaligned homologous sequences. *Bioinformatics* **34**, 3929–3930 (2018).

103. R. C. Edgar, MUSCLE: Multiple sequence alignment with high accuracy and high throughput. *Nucleic Acids Res.* **32**, 1792–1797 (2004).

104. S. Capella-Gutiérrez, J. M. Silla-Martínez, T. Gabaldón, trimAl: A tool for automated alignment trimming in large-scale phylogenetic analyses. *Bioinformatics* **25**, 1972–1973 (2009).

105. J. A. Ballesteros, G. Hormiga, A new orthology assessment method for phylogenomic data: Unrooted phylogenetic orthology. *Mol. Biol. Evol.* **33**, 2117–2134 (2016).

106. M. dos Reis, Z. Yang, Approximate likelihood calculation on a phylogeny for Bayesian estimation of divergence times. *Mol. Biol. Evol.* **28**, 2161–2172 (2011).

107. H. C. Betts, M. N. Puttick, J. W. Clark, T. A. Williams, P. C. J. Donoghue, D. Pisani, Integrated genomic and fossil evidence illuminates life's early evolution and eukaryote origin. *Nat. Ecol. Evol.* **2**, 1556–1562 (2018).

108. R. J. Howard, M. Giacomelli, J. Lozano-Fernandez, G. D. Edgecombe, J. F. Fleming, R. M. Kristensen, X. Ma, J. Olesen, M. V. Sørensen, P. F. Thomsen, M. A. Wills, P. C. J. Donoghue, D. Pisani, The Ediacaran origin of Ecdysozoa: Integrating fossil and phylogenomic data. *J. Geol. Soc. London* **179**, doi.org/10.1144/jgs2021-107 (2022).

109. K. Angelis, S. Álvarez-Carretero, M. Dos Reis, Z. Yang, An evaluation of different partitioning strategies for bayesian estimation of species divergence times. *Syst. Biol.* **67**, 61–77 (2018).

110. L. J. Revell, phytools: An R package for phylogenetic comparative biology (and other things). *Methods Ecol. Evol.* **3**, 217–223 (2012).

111. M. Pagel, Detecting correlated evolution on phylogenies: A general method for the comparative analysis of discrete characters. *Proc. Royal Soc. London B* **255**, 37–45 (1997).

112. S. Álvarez-Carretero, A. U. Tamuri, M. Battini, F. F. Nascimento, E. Carlisle, R. J. Asher, Z. Yang, P. C. J. Donoghue, M. dos Reis, A species-level timeline of mammal evolution integrating phylogenomic data. *Nature* **602**, 263–267 (2022).

113. D. L. Rabosky, M. Grundler, C. Anderson, P. Title, J. J. Shi, J. W. Brown, H. Huang, J. G. Larson, BAMMtools: An R package for the analysis of evolutionary dynamics on phylogenetic trees. *Methods Ecol. Evol* **5**, 701–707 (2014).

114. J. J. Matthews, A. G. Liu, C. Yang, D. M. Ilroy, B. Levell, D. J. Condon, A chronostratigraphic framework for the rise of the Ediacaran macrobiota: New constraints from Mistaken Point Ecological Reserve, Newfoundland. *Geol. Soc. Am. Bull.* **133**, 612–624 (2021).

115. F. S. Dunn, C. G. Kenchington, L. A. Parry, J. W. Clark, R. S. Kendall, P. R. Wilby, A crown-group cnidarian from the Ediacaran of Charnwood Forest, UK. *Nat. Ecol. Evol.* **6**, 1095–1104 (2022).

116. Z. Yin, W. Sun, P. Liu, M. Zhu, P. C. J. Donoghue, Developmental biology of Helicoforamina reveals holozoan affinity, cryptic diversity, and adaptation to heterogeneous environments in the early Ediacaran Weng'an biota (Doushantuo Formation, South China). *Sci. Adv.* **6**, eabb0083 (2020).

117. Z. Yin, K. Vargas, J. Cunningham, S. Bengtson, M. Zhu, F. Marone, P. Donoghue, The early Ediacaran Caveasphaera foreshadows the evolutionary origin of animal-like embryology. *Curr. Biol.* **29**, 4307–4314.e2 (2019).

118. C. Yang, A. D. Rooney, D. J. Condon, X.-H. Li, D. V. Grazhdankin, F. T. Bowyer, C. Hu, F. A. Macdonald, M. Zhu, The tempo of Ediacaran evolution. *Sci. Adv.* **7**, eabi9643 (2021).

119. M. J. Benton, P. C. J. Donoghue, R. J. Asher, M. Friedman, T. J. Near, J. Vinther, Constraints on the timescale of animal evolutionary history. *Palaeont. Electr.* **18**, 1–106 (2015).

120. X.-P. Dong, J. A. Cunningham, S. Bengtson, C.-W. Thomas, J. Liu, M. Stampanoni, P. C. J. Donoghue, Embryos, polyps and medusae of the Early Cambrian scyphozoan Olivooides. *Proc. Biol. Sci.* **280**, 20130071 (2013).

121. F. T. Bowyer, A. Y. Zhuravlev, R. Wood, G. A. Shields, Y. Zhou, A. Curtis, S. W. Poulton, D. J. Condon, C. Yang, M. Zhu, Calibrating the temporal and spatial dynamics of the Ediacaran-Cambrian radiation of animals. *Earth Sci. Rev.* **225**, 103913 (2022).

122. M. Steiner, G. Li, Y. Qian, M. Zhu, B.-D. Erdtmann, Neoproterozoic to early Cambrian small shelly fossil assemblages and a revised biostratigraphic correlation of the Yangtze Platform (China). *Palaeogeogr. Palaeoclimatol. Palaeoecol.* **254**, 67–99 (2007).

123. B. Runnegar, Muscle scars, shell form and torsion in Cambrian and Ordovician univalved molluscs. *Lethaia* **14**, 311–322 (1981).

124. M. Steiner, G. Li, Y. Qian, M. Zhu, Lower Cambrian Small Shelly Fossils of northern Sichuan and southern Shaanxi (China), and their biostratigraphic importance (Small Shelly Fossils du Cambrien inférieur du nord du Sichuan et du sud du Shaanxi (Chine) et leur importance biostratigraphique). *Geobios* **37**, 259–275 (2004).

125. H. Mostler, Mikroskleren von demospongien (Porifera) aus dem basalen Jura der nördlichen Kalkalpen. *Geo.-Paläontol. Mitt. Innsbruck* **17**, 119–142 (1990).

126. M. Łukowiak, Fossil and modern sponge fauna of southern Australia and adjacent regions compared: Interpretation, evolutionary and biogeographic significance of the late Eocene ‘soft’ sponges. *Smithson. Contrib. Zool.* **85**, 13–35 (2016).

127. N. P. James, Y. Bone, Eocene cool-water carbonate and biosiliceous sedimentation dynamics, St Vincent Basin, South Australia. *Sedimentology* **47**, 761–786 (2000).

128. K. A. Ritterbush, S. Rosas, F. A. Corsetti, D. J. Bottjer, A. J. West, Andean sponges reveal long-term benthic ecosystem shifts following the end-Triassic mass extinction. *Palaeogeogr. Palaeoclimatol. Palaeoecol.* **420**, 193–209 (2015).

129. D. Ungureanu, F. Ahmad, S. Farouk, A Callovian (Middle Jurassic) poriferan fauna from northwestern Jordan: Taxonomy, palaeoecology and palaeobiogeography. *Hist. Biol.* **30**, 577–592 (2018).

130. T. Schindler, M. Wuttke, M. Poschmann, Oldest record of freshwater sponges (Porifera: Spongillina)—Spiculite finds in the Permo-Carboniferous of Europe. *Paläontol. Z.* **82**, 373–384 (2008).

131. S. Fortunato, M. Adamski, B. Bergum, C. Guder, S. Jordal, S. Leininger, C. Zwafink, H. T. Rapp, M. Adamska, Genome-wide analysis of the sox family in the calcareous sponge Sycon ciliatum: Multiple genes with unique expression patterns. *Evodevo* **3**, 14 (2012).
